# Pollinators support the nutrition and income of vulnerable communities

**DOI:** 10.1038/s41586-026-10421-x

**Published:** 2026-05-06

**Authors:** T. P. Timberlake, S. Sapkota, N. M. Saville, A. R. Cirtwill, S. C. Baral, D. R. Bhusal, K. Devkota, S. Giri, H. A. Harris-Fry, D. Joshi, S. Kortsch, S. S. Myers, T. Roslin, M. R. Smith, J. Memmott

**Affiliations:** 1 School of Biological Sciences, University of Bristol, Bristol, UK; 2 Department of Environment and Geography, University of York, York, UK; 3 HERD International, Kathmandu, Nepal; 4 Institute for Global Health, University College London, London, UK; 5 Department of Agricultural Sciences, University of Helsinki, Helsinki, Finland; 6 Carex EcoLogics, Bracebridge, Ontario Canada; 7 Central Department of Zoology, Tribhuvan University, Kathmandu, Nepal; 8 Faculty of Agriculture, Agriculture and Forestry University, Chitwan, Nepal; 9 Department of Population Health, London School of Hygiene and Tropical Medicine, London, UK; 10 Tvärminne Zoological Station, Faculty of Biological and Environmental Sciences, University of Helsinki, Hanko, Finland; 11 Department of Environmental Health and Engineering, Johns Hopkins Bloomberg School of Public Health, Baltimore, MD USA; 12 Johns Hopkins Institute for Planetary Health, Baltimore, MD USA; 13 Department of Environmental Health, Harvard T. H. Chan School of Public Health, Boston, MA USA; 14 Department of Environmental and Occupational Health Sciences, School of Public Health, University of Washington, Seattle, WA USA

**Keywords:** Agroecology, Nutrition, Ecosystem services, Developing world, Ecological networks

## Abstract

Biodiversity loss threatens human health and welfare through the degradation of ecosystem services like pollination^1–3^. However, without clear mechanistic links between ecosystems and people, these services can remain abstract and intangible. Consequently, it is challenging to predict the effects of environmental degradation on human welfare or to identify effective ecological interventions that improve human lives. Here we record individual-level diets, crop yields, farming income and crop–pollinator interactions in replicate smallholder communities in Nepal to quantify the links among insect pollinators, crop plants and nutrient intake and income of individual families. Insect pollinators were directly responsible for 44% of people’s farming income and more than 20% of their vitamin A, folate and vitamin E intake. We show how declines in local pollinator species are anticipated to exacerbate rates of poverty and micronutrient deficiency in vulnerable communities such as the ones studied here. However, our results demonstrate that management of local pollination services can improve human nutrition and household income. Indeed, abundant pollinators like native honeybees, bumblebees and hoverflies are the most important for sustaining and enhancing nutrient flows. Applied more widely, this approach of linking biodiversity to human health and livelihoods could reveal sustainable new pathways for improving the lives of millions of smallholders worldwide.

## Abstract (web summary)

Quantification of links among insect pollinators, crop plants, nutrient intake and income of smallholder communities in Nepal show that pollinators substantially contribute to their income and micronutrient intake.

## Main

Biodiversity underpins almost every aspect of human health. Clean air, fresh water, disease regulation and food production all rely on the functioning of healthy ecosystems^3,4^. Rapid habitat and species loss and the disruption of ecological interactions therefore jeopardize the health and wellbeing of people, particularly in low-income contexts in which rates of ill health and poverty are already high^2,5,6^. To address this risk, we must understand and harness the pathways that link biodiversity to human health^7^.

Pollination is an example of a key relationship between ecosystems and human health that is being degraded through shifting environmental and anthropogenic pressures^8–10^. Pollination supports the production of 75% of the world’s crop species, including many of the most nutritious crops that provide a large proportion of our key micronutrients, such as vitamin A, vitamin C and folate^11,12^. Inadequate intake of these micronutrients leads to increased mortality from infectious disease and birth defects, as well as impaired physical and cognitive development. Together, such outcomes contribute to intergenerational cycles of poverty and ill health^13,14^. One-quarter of the global population currently suffer from this ‘hidden hunger’^15^. Ongoing declines in pollinator species are anticipated to further degrade global health by reducing the yields and consumption of pollinator-dependent micronutrient-rich crops, which in turn may lead to increased rates of mortality and illness^1,16–18^. Reductions in pollination services will also result in substantial economic losses^19^, which are expected to further exacerbate rates of malnutrition^20^. These pressures will be felt most acutely by the 2 billion smallholders in low-income countries, where rates of poverty and malnutrition and reliance on pollinator-dependent crops are highest^21–24^.

Ecosystem services result from the actions of individual organisms (for example, the transport of crop pollen by an insect), and the beneficiaries of these services are individual people^25,26^. However, without studying the sequence of interactions that lead from organisms to people, the concept of ecosystem services can remain abstract and intangible.Consequently, it is challenging to predict the real effects of environmental degradation on human health or to identify practical interventions that enhance ecosystem services in a way that optimizes human health.

In this study, we characterize the plant–pollinator community that underpins human nutrient acquisition in a real-world system and evaluate changes in individual-level diets and income under future scenarios of pollinator population change. We identify features of pollinator taxa that best predict their nutritional importance and identify pathways for enhancing human nutrition through the management of pollination services. Our study takes place in Nepal, where the majority of the population (70%) rely directly on smallholder farming for their nutrition and income, which makes them heavily dependent on local ecosystem services such as pollination^27^. The smallholder communities in our study region of Jumla District exemplify many of the agricultural, demographic and economic characteristics of smallholder farmers globally and fall within the range of variation reported in a global data portrait of 19 representative smallholder populations^28^ (Supplementary Fig. 1). Moreover, on the basis of local data from native honeybee populations, Jumla District seems to be facing similar levels of decline in pollinator populations as smallholder farming regions elsewhere^24,29^. Therefore, this region represents a useful example to demonstrate generalizable patterns and processes.

**Fig. 1:**
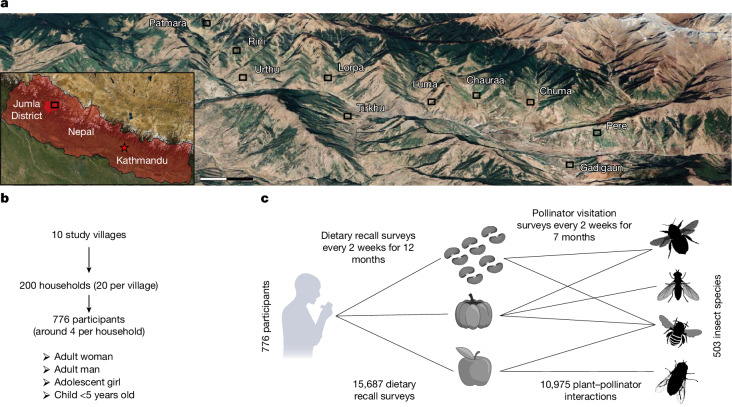
Study sites, study population and sampling regime. **a**, Map showing the villages in Jumla District, Nepal, that were included in the study. Village locations are indicated by black boxes; inset shows the study area in Nepal. Scale bar, 2 km. **b**, Overview of the study population and participant recruitment. **c**, Schematic of the dietary and ecological sampling design. Base map in **a** from ESRI. Insect icons in panel **c** reproduced from PhyloPic: CC BY 4.0 (*Melangyna novaezelandiae* and *Amegilla*), CC BY 3.0 (*Bombus terrestris*) and CC0 1.0 (*Syrphidae*).

Working in replicate smallholder communities, we record individual-level diets, crop yields, farming income and plant–pollinator visitation to quantify the connections between pollinator species, crop plants and nutrient intake and income of individual families (Fig. 1). We then predict how changes in the pollinator community (both declines and increases) could affect the nutrition and livelihoods of people. Specifically, we address four questions: (1) which plant–pollinator interactions underpin human nutrition; (2) how changes in the pollinator community affect human nutrition and livelihoods; (3) which pollinator characteristics best predict their contribution to human nutrition; and (4) how pollinators can be managed to enhance human nutrition. Our findings provide a crucial test case and offer an approach and insights relevant for improving human health and livelihoods on a global scale.

## Connecting pollinators to human diets

**Fig. 2:**
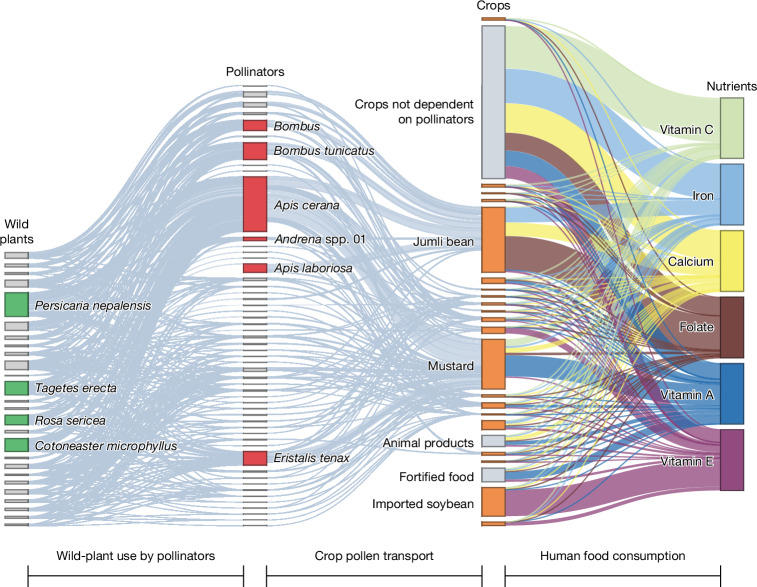
Ecological interactions that underpin human nutrient intake. The micronutrient intake of smallholder families relies on a range of pollinator-dependent crops (orange), a community of insects that pollinate them (red) and a range of supporting wild plants that sustain the insect populations with food (green). Non-pollinator-dependent foods are shown in grey. The coloured flows show the proportion of total micronutrient intake provided by each crop, and the width of each crop bar shows its summed contribution to all six micronutrients. The width of pollinator bars (red) and wild-plant bars (green) represent their relative contribution to human nutrition via their pollination of key crops and their support of key pollinators, respectively.

Our study was conducted in ten smallholder farming villages in Jumla District of Nepal (Supplementary Figs. 2–4). Within these villages, we recorded the diets, nutritional status, farming practices and socioeconomic status of 776 individuals throughout a 12-month period using replicate high-resolution longitudinal surveys (Fig. 1). This approach enabled us to determine the source of each nutrient in people’s diet (for example, the crops or animals each nutrient is derived from) and to estimate levels of poverty and malnutrition throughout the year. In parallel, we conducted plant–pollinator visitation surveys every 2 weeks throughout the flowering season to identify the insects that visit locally consumed crops (Fig. 1 and Supplementary Tables 1 and 2). We used this visitation data, in combination with analyses of the pollen loads of the insects, to estimate the contribution of each insect species to the pollination of each crop that was consumed (accounting for differences in pollinator dependence between crops; Methods). By linking this crop–pollinator network to data on crop–nutrient provision, we quantified the sequence of interactions leading from plant and insect species to human nutrient acquisition (Fig. 2). These are, to our knowledge, the first empirical links established between insect pollinator species and human diets, and they enabled us to estimate the role of individual species in supporting human health. Wild pollinators (primarily bumblebee and hoverfly species; Supplementary Table 3) collectively provided most of the pollen transport services that underpin human nutrition. However, the native semi-domesticated honeybee *Apis cerana* was the most important single species. An estimated 7% of vitamin A intake, 5% of folate and 5% of calcium was directly attributable to this species (Fig. 2 and Supplementary Table 3). The most important pollinator taxa (including *A. cerana*, *Bombus tunicatus*, *Apis laboriosa* and *Eristalis tenax*) were similar across the ten villages studied (Supplementary Fig. 5).

**Fig. 3:**
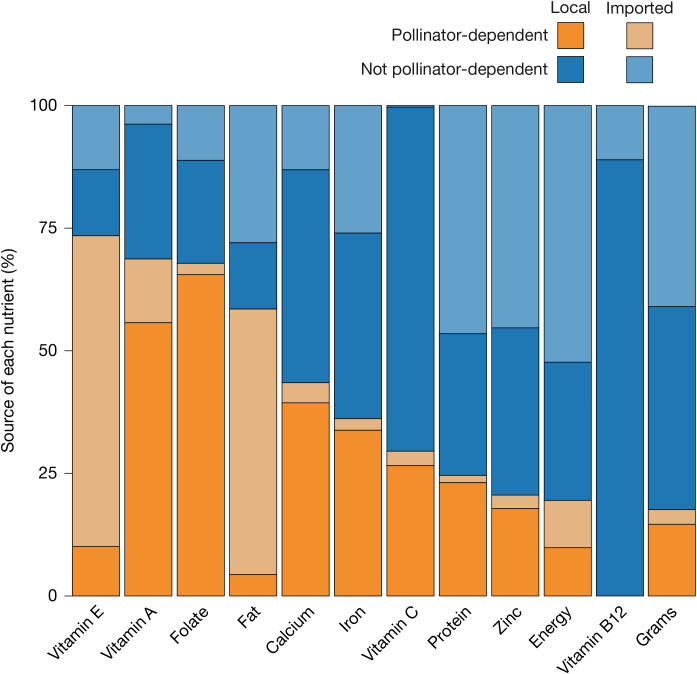
Source of key nutrients in the diet. For each key nutrient, stacked bars show the share of intake supplied by pollinator-dependent versus non-pollinator-dependent foods, which is further split into local (grown or raised within Jumla District; dark shades) and imported (light shades). Percentages reflect total intake pooled across all study participants (*n* = 776 individuals) over the study year. The rightmost bar shows the same breakdown for total grams of food consumed. Apart from vitamin E and fat (mostly sourced from imported vegetable oils), highly pollinator-dependent nutrients (vitamin A, folate, calcium, iron and vitamin C) are almost exclusively sourced from local foods.

For most smallholder communities around the world, a substantial proportion of their diet comes from local agricultural produce^28^, which creates a direct trophic link with their local agroecosystem^27^. In our study region, more than 80% of people’s intake of key micronutrients (vitamin C, folate, vitamin A, calcium and vitamin B12) came from locally grown crops and animal products. This result highlights that the nutrition of these smallholder communities is heavily reliant on local agricultural production and the ecosystem services that support it (Fig. 3 and Supplementary Fig. 6). By contrast, a large proportion of people’s macronutrients (energy, fat and protein) were derived from foods that are mostly imported, including rice and vegetable oil. Access to macronutrients therefore remained relatively constant throughout the year, whereas access to micronutrients (from local fruits and vegetables) showed a strong seasonal pattern. Indeed, fruit and vegetable consumption changed by more than twofold throughout the year in response to seasonal agricultural patterns (Supplementary Fig. 7).

Only 18% of people’s diets (by weight) came from pollinator-dependent crops, but these crops (mostly vegetables, pulses and fruits; Supplementary Table 4) contributed to a large proportion of their key dietary micronutrients, including 73% of total vitamin E intake, 68% of folate, 67% of vitamin A, 44% of calcium and 36% of iron (Fig. 3 and Supplementary Figs. 8 and 9). Our estimates are probably conservative, as they only consider the contribution of pollinators to crop yields and not the additional benefits to crop quality (including nutritional quality)^30^. Nutritional reliance on pollination was similar to estimates from a previous study^16^ that analysed the share of diets provided by pollinator-dependent crops in Zambia, Uganda, Mozambique and Bangladesh. Smallholder families in these countries relied to a similar degree on pollinator-dependent vegetables, pulses and fruits for their vitamin A, folate, calcium and iron intake, which suggests that these nutrients may be relatively consistent in their pollinator dependence (Supplementary Fig. 10). However, by going a step further and characterizing the plant–pollinator community that underpins the production of these nutrients, we can explore the ecological pathways to support and improve human nutrition.

## Pollinator loss affects diets and income

Severe declines in pollinator populations are predicted for many smallholder farming regions across the world^24^. Moreover, evidence of local declines in native honeybee populations^29^ suggests that similar pressures are already occurring in our study region. Given the heavy reliance of smallholder families on micronutrient-rich pollinator-dependent crops, we wanted to estimate the impact of declines in local pollinator species on people’s nutrition and livelihoods. Our measurements of people’s diets, nutritional status and household income showed that levels of malnutrition and poverty are extremely high in our study population. In detail, 51% of children aged 6–59 months were stunted (low height-for-age), 17% were severely stunted, 24% were underweight (low weight-for-age) and 5% were wasted (low weight-for-height; Supplementary Table 5). The probability of individuals in the study population achieving dietary adequacy (that is, meeting their requirements) was only 2% for calcium, 3% for vitamin A, 12% for iron and 59% for folate (see Supplementary Table 6 for other nutrients). These values are among the lowest recorded anywhere^31^, and adequacy was even lower for children and adolescent girls (Supplementary Table 6). Nutritional challenges in this region stem from both food-system and health-related factors. Low agricultural productivity, poor access to inputs and services, reliance on local production without adequate markets or storage and the rise of nutrient-poor processed foods all combine to limit diet quality. These issues are compounded by suboptimal child feeding, recurrent infections, high energy demands from labour-intensive livelihoods and uneven access to health and nutrition services^32^.

Against this backdrop, declines in pollination services represent an additional challenge that we aimed to quantify. To do so, we simulated incremental declines in local pollinator abundance (0–100%) and estimated the resulting changes in individual nutrient intake, probability of nutrient adequacy and household farming income. Pollinator taxa were assigned different decline rates in each replicate simulation to account for species-specific variation in sensitivity to environmental change (Methods). Although we modelled the full gradient of decline, we highlight two illustrative scenarios: (1) complete loss of local pollinators, which represents an extreme outcome; and (2) a ‘business as usual’ trajectory, for which we make a conservative best-guess estimate of declines in the pollinator community by 2030 based on local data showing declines in native honeybee populations. We also modelled a third condition: a recovery scenario, in which farmers actively manage pollination services so that pollination is no longer a yield-limiting factor for their crops. In all cases, only the pollinator-dependent share of the yield for each crop was adjusted. This modification reflects the fact that most crops are partially rather than fully pollinator dependent^11^ and that many other factors (beyond the scope of this study) can limit crop yield. Our simulations made several simplifying but necessary assumptions (Methods); therefore, they do not provide precise forecasts but rather boundary estimates of potential outcomes in the absence of mitigating actions. Sensitivity analyses of key assumptions are provided in Supplementary Method 1.

**Fig. 4:**
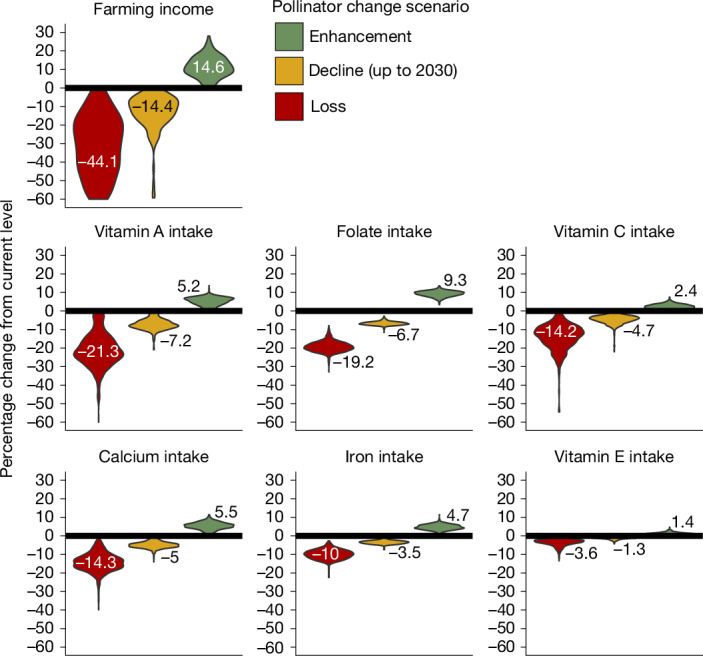
Predicted impacts of local pollinator loss, decline and enhancement on human welfare. Simulated changes in the local pollinator community alter the micronutrient intake and farming income of smallholder communities, with each panel showing a different welfare outcome. Each violin shows the distribution of predicted outcomes across all study participants (*n* = 776 individuals), with colours indicating the pollinator change scenario and mean values shown in the text. Pollinator-driven yield changes were applied only to local crops. Therefore, vitamin E (mostly from imported vegetable oils; Fig. 2) shows little change despite high pollinator dependence.

**Fig. 5:**
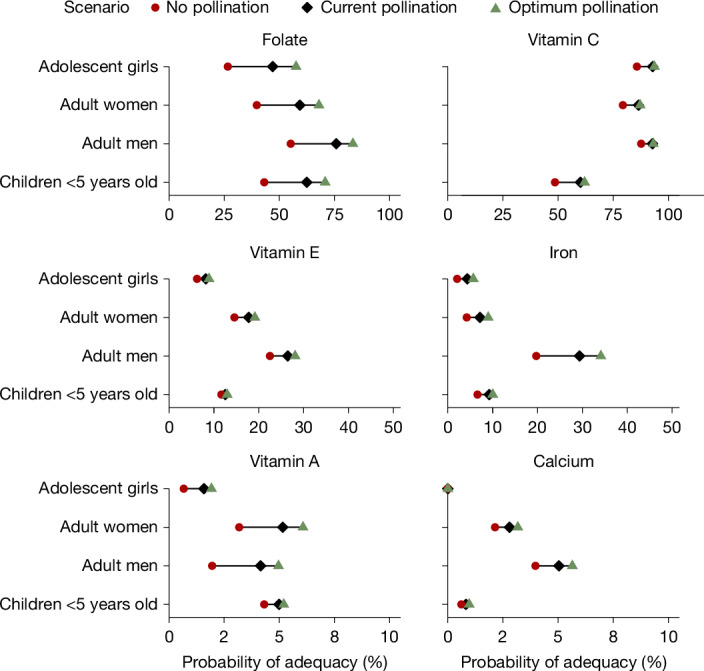
Range of dietary adequacy attributable to pollination. Plots show the probability of dietary adequacy for different micronutrients (shown in separate panels) and population subgroups (separate rows) across the simulated pollination gradient, from no pollination (red circles) to optimum pollination (green triangles). Analyses were based on *n* = 776 individuals, comprising *n* = 215 adult women, *n* = 186 adult men, *n* = 190 adolescent girls and *n* = 185 children under 5 years of age. Black horizontal lines connect the minimum and maximum values, which indicate the range attributable to pollination. Standard deviations for each estimate are provided in Supplementary Table 8. The results highlight that although many factors shape nutrition, changes in pollination can meaningfully shift adequacy for some key nutrients. Note that the *x* axis scales differ by panel row.

Across our simulations, declines in local pollinator populations caused substantial reductions in nutrient intake and household income. The magnitude of impact depended on the severity of pollinator decline and the nutrient in question (Supplementary Fig. 11). Vitamin A, folate, vitamin C and calcium were the nutrients most affected by declines in pollinator populations. Under an extreme scenario of complete loss of local pollinators, we predicted reductions of 21% (±9 s.d.) in vitamin A intake, 19% (±3 s.d.) for folate, 14% (±8 s.d.) for vitamin C and 14% (±5 s.d.) for calcium (Fig. 4 and Supplementary Table 7). Under a business-as-usual trajectory, for which we made a best-guess estimate of declines in pollinator populations by 2030, we estimated smaller but still significant reductions of 7% (±3 s.d.) in vitamin A intake and 7% (±1 s.d.) in folate intake (Fig. 4 and Supplementary Table 7). Trends were broadly similar across villages (Supplementary Fig. 12) and population subgroups (Supplementary Fig. 13), although impacts were most severe for adolescent girls, who obtain a slightly higher proportion of their key nutrients from pollinator-dependent crops (Supplementary Fig. 8). These estimates reflected declines in locally produced foods only, and impacts were even more severe when imported pollinator-dependent foods were included (Supplementary Table 7). These changes would further diminish the probability of individuals meeting their nutrient requirements (Fig. 5 and Supplementary Table 8), deepen existing deficiencies and—under the extreme scenario—put an additional 17% of the population at risk of deficiency in at least one micronutrient. For vitamin A, worsened nutrient status below the deficiency threshold is associated with progressively more severe consequences for eyesight, ranging from night blindness to complete blindness in extreme cases^33^. Similarly, for folate, although many women in our study population already consume less than the estimated requirement, further reductions in intake are anticipated to increase the risk of neural tube defects in children in utero^34^.

Adding to these negative impacts, a complete loss of local pollinators was predicted to cause a 44% (±40 s.d.) reduction in household farming income, whereas the business-as-usual trajectory predicted a 14% (±13 s.d.) reduction by 2030 (Fig. 4). These predicted economic impacts are primarily driven by reductions in the yields of apples and beans, which are the two major cash crops in this region. Because the market value of these crops is shaped by larger regional markets rather than local supply, reduced yields are not anticipated to translate into higher local prices (Supplementary Method 1, assumption 6).

Given that nutrient intake and household income both affect growth rates and disease prevalence^35^, the changes resulting from pollinator decline are likely to limit children’s growth and to increase rates of ill health and mortality in this already nutritionally compromised population. Our results are consistent with coarser global modelling studies^1,24^, which indicated widespread economic and nutritional vulnerability to declines in pollinator populations. Such vulnerabilities are particularly stark in smallholder farming regions where populations lack the economic flexibility and market access to change their farming practices or diets^2,27^. However, unlike global-scale analyses, our approach links individual-level dietary and livelihood data directly to observed ecological networks. This approach enabled us to quantify nutritional and economic risks with greater confidence and to investigate the underlying drivers.

Notably, our simulations also suggested that these risks are not inevitable. If farmers manage pollination services to ensure that local crops are no longer pollen-limited (therefore reducing the proportion of the yield gap of a crop attributed to insufficient pollination^36^), we predicted a 15% (±16 s.d.) increase in household income (up to 30% in some cases; Supplementary Fig. 12). Moreover, we predicted a 9% (±2 s.d.) increase in folate intake and a 5% (±2 s.d.) increase in vitamin A intake (Fig. 4 and Supplementary Table 7). We estimate that this strategy would bring 9% of the population into sufficiency for at least one micronutrient and reduce the depth of deficiency for many others.

Achieving favourable economic and nutritional status in smallholder farming communities will naturally require more than optimal pollination services. Direct interventions such as micronutrient supplementation and improved child feeding practices are likely to be required^37^ alongside multisectoral approaches such as agricultural extension, crop biofortification, social and behaviour change interventions and market-based strategies^38^. These can all work to increase yields, reduce post-harvest losses, increase budgets and create demand and consumption of nutrient-dense foods. However, most of these interventions still depend on the underlying pollination service to maintain crop yields.

## Drivers of pollinator importance

To extend the generality of our findings beyond the specific pollinator taxa found in this region, we used our plant–pollinator network to investigate whether the role of the pollinator in that network can predict its contribution to human nutrition (Methods). Pollinator abundance was a strong predictor of nutritional importance (*R*^2^ = 0.42, *t*_183_ = 12.63, *P* < 2 × 10^–16^; Supplementary Fig. 14), and the most important pollinators (for example, *Apis* spp., *Bombus* spp., *Eristalis* spp. and *Lucilia* spp.) tended to occupy more central positions in the network and to direct a greater proportion of their visits to crop species as opposed to wild plants (Supplementary Fig. 15). Although these taxa also interacted with many plant species, abundance-standardized measures of interaction breadth, evenness and selectivity did not explain additional variation in nutritional importance beyond the abundance-only model (Supplementary Table 9). These results suggest that nutritional benefits are largely explained by the abundance of the pollinator, a result consistent with work in other regions where abundant taxa perform the bulk of crop pollination^39,40^. Our analysis, however, reflected only a single year, and reliance on a few dominant species may provide a fragile foundation for long-term food security^41^. Functional diversity and redundancy offer ecological insurance by buffering services against environmental fluctuations and long-term change^42^. Such a strategy is an important consideration in our study system, for which even the most abundant species (*A. cerana*) is showing signs of decline^29^.

## Improving nutrition through pollination

An ecosystem service such as pollination does not exist in isolation; its provision is influenced by a wide range of other species and ecosystem conditions. For example, the abundance of any given crop pollinator may be influenced by the availability of its preferred food plants, the availability of suitable nesting sites and its interactions with harmful agents such as predators or pesticides^9,43–45^. By extending the network of links among nutrients, crops and pollinators to include features of the ecosystem that influence these pollinators, we can begin to understand the role of broader ecosystem dynamics that underpin human nutrition. For example, it may become clear that the removal of particular wild plant species is likely to have cascading negative effects on human nutrition by affecting the pollinators that feed on it. Conversely, we can identify plant species or habitat features with the greatest likelihood of benefitting human nutrition. To illustrate this point, we extended the nutrient–crop–pollinator network to include the wild plant species that pollinators were recorded to feed on (Fig. 2). Links between pollinators and wild plants were weighted by the frequency of their interaction and the estimated importance of each pollinator to micronutrient provision, which enabled us to identify plants with the greatest leverage on human nutrition.

In our study region, the following plants had the greatest predicted influence on human nutrition: the agricultural weeds *Persicaria nepalensis*, *Cirsium wallichii* and *Galinsoga ciliata*; the wild shrubs *Cotoneaster microphyllus* and *Rosa sericea*; and the ornamental plants *Tagetes erecta* and *Cosmos bipinnatus* (Fig. 2 and Supplementary Table 10). All of these plants can be readily grown in field margins, hedgerows and gardens to support crop pollinators. Wild plants like these support crop pollinators with floral resources outside the main periods of crop flowering, thereby facilitating crop pollination (Supplementary Fig. 16). These plants were consistently important across the ten study villages (Supplementary Fig. 5). They offer a locally targeted management option to be applied by farmers alongside more generic interventions such as pesticide reduction, small-scale native beekeeping and the provision of nesting habitats^41^. All these management strategies, including the keeping of native honeybees (*A. cerana*) and the selection and placement of plants, must of course be assessed by farmers themselves to avoid negative ecological impacts (for example, disease transmission by honeybees), minimize ecosystem disservices (for example, introduced plants competing with crops) and ensure that they are appropriate to the local context. Evidence from our study region showed that farmers were able to make such assessments and to apply pollination management practices at minimal cost once they understood the value of pollination services^41^.

Although many other agricultural inputs and ecosystem services need to be managed to improve crop yields and nutrition, pollination management offers a complementary intervention that is widely effective, affordable and accessible to resource-constrained farmers. Moreover, because insect-pollinated crops are rich in micronutrients^16^, pollination management is likely to deliver particularly strong benefits for human nutrition.

Our study provides an empirical demonstration of the reliance of human populations on a broad suite of wild species and ecosystem processes beyond those that are most obvious. Although this reliance is most direct and tractable in subsistence farming communities, the same chain of reliance between people and pollinators is present for populations around the world, albeit through substantially more complex and diffuse food system pathways^46^.

## Conclusion

Our results emphasize the fact that all species are embedded in a broader web of trophic and non-trophic interactions. This concept applies as much to humans as it does to any other species. By mapping the sequence of interactions—from pollinators to people—we provided a clear example of the crucial but under-appreciated role of the many different ecosystem components that support human health and welfare. This framework shows how the concept of ecosystem services can be made tangible, measurable and ultimately predictable to provide a practical pathway for simultaneously enhancing both human health and biodiversity.

## Methods

### Study sites

Field work took place in ten smallholder farming villages (2,400–3,000 metres above sea level, temperate climate) in Jumla District, Nepal (Fig. 1, Supplementary Fig. 2 and Supplementary Method 2). Jumla is a remote mountainous district, situated in the Karnali Province of western Nepal. Rates of poverty, food insecurity and malnutrition are very high in this region, and 80% of the population directly depends on smallholder agriculture^47^. The study populations are typical of many smallholder communities around the world (Supplementary Fig. 1) and are characterized by a heavy reliance on small (<2 ha) family-run farms for both their income and nutrition, which makes them heavily dependent on local ecosystem services^27^. Each study village comprised a cluster of 100–400 closely spaced households interspersed with small vegetable gardens and livestock enclosures. Villages were surrounded by many small (0.01–0.3 ha) arable fields and apple orchards as well as large areas of steep, heavily grazed pasture and native forest (Supplementary Figs. 3 and 4). More than 50 crops are grown in this region, including many pollinator-dependent species such as apples, beans, pumpkins, mustard and buckwheat (Supplementary Tables 2–4).

### Study population

Households were considered eligible for inclusion in the study if they were permanent residents in the community (spent at least 10 months of the year in the village) and had at least three out of the four respondent categories in the household (adult man, adult woman, adolescent girl and child under 5 years of age). On the basis of a full census of each village, we identified eligible households, randomly selected 20 from each village and obtained consent from the head of the household. From each household, we aimed to enrol one adult woman, one adult man, one adolescent girl and one child under the age of 5 years as participants in the study. This selection provided a diverse picture of diets in each household, including two particularly vulnerable subgroups: young children (owing to their rapid growth and high nutrient needs) and adolescent girls (who may soon have their first child and whose pre-conception nutrition strongly influences maternal and child health outcomes). Adolescent boys were not included as a separate respondent category as their diets are assumed to be more similar to adult men and they are considered less nutritionally vulnerable than adolescent girls. Participants were considered eligible for inclusion in the study if they were permanent residents of the household and not suffering with an illness or disability that prevented them eating a usual diet or responding to questions (Supplementary Method 3, selection of study participants). Our final study population (Supplementary Table 11) consisted of 776 individuals (215 adult women, 186 adult men, 190 adolescent girls and 185 children under 5 years of age).

### Ethics statement

Ethical approval for this study was obtained from the Ethical Review Board of the Nepal Health Research Council (reference 1709) and the Faculties of Life Sciences and Science Research Ethics Committee at the University of Bristol (reference 102982). All procedures involving human participants were conducted in accordance with the relevant institutional and national ethics guidelines. For all participants over 18 years of age, informed consent (signature or thumb print) was obtained; for all participants under 18 years, consent was provided by their parent or guardian and the adolescent girls also provided assent. Participants’ time was remunerated with mobile phone credit vouchers, a widely used and valued resource in the study region.

### Dietary-recall surveys and recipe collection

Dietary-recall surveys were conducted for each participant every 2 weeks for an entire year (November 2021 to November 2022). During each recall event (24 in total), we recorded the identity and quantity of every food item consumed by respondents during the previous 24 h. To minimize recall bias, we used a five-stage multipass food-recall method^48^ (Supplementary Method 3, dietary-recall surveys). Food models were used to estimate portion sizes: cooked rice as a model for rice and other irregular-sized solid foods such as vegetable curries and cooked green leaves; water for portions of dal (soup of beans or pulses), milk or other liquids; playdough for chunks of meat or pieces of fruit; wheat flour for powdered foods like roasted grain flour, salt and chili powder; and dried corn for dried or roasted nuts and grains. Participants were instructed to only report the food that was consumed and not any that was leftover on the plate. Food models were weighed using Salter kitchen scales and then back-converted to estimate the mass of each food item consumed, accounting for differences in food density (Supplementary Method 3, dietary-recall surveys). For foods in packets, or of a standard size, the number of items consumed was recorded. Interviews were conducted by trained data collectors in the Nepali language and information was entered into a customized data-collection form using the cloud-based data-collection platform CommCare (v.2.49; http://www.commcarehq.org/home/) on an Android tablet. All data collection had range checks and internal validity checks built in to help maintain quality control.

To identify the composition of each mixed dish (that is, the quantity of each ingredient in it), we asked the lead cook in replicate local households to prepare the food following their normal recipe (Supplementary Method 4). Each ingredient was weighed as it was added to the cooking dish and then the final weight of the prepared meal was recorded and the proportional composition of each ingredient (by weight) was calculated. For each ingredient in a recipe, we calculated its mean proportional contribution to the recipe (food item) based on the raw weight of edible ingredients across all recipe replicates collected (ten in most cases). For each food item consumed by a participant, we multiplied the grams of food item consumed by the proportional content of each ingredient to determine the grams of each individual ingredient consumed on a given date. We complied a Nepal-specific food composition table from a range of published sources (Supplementary Method 4, food composition data sources), which was used to assign a nutritional composition to each ingredient. Nutrient values for each ingredient were adjusted for cooking losses using the USDA Table of Nutrient Retention Factors (release 6, 2007), applying the appropriate factor for each cooking method (for example, boiled versus fried potato) to ensure that nutrient values reflected the ingredients in the form they were eaten (Supplementary Method 4, nutrient retention factors).

### Height and weight measurements

For each study participant, measurements of their height and weight were taken once per month throughout the survey period to assess their nutritional status. Standing height was recorded using Shorr Board Stadiometers for adults, adolescents and children over 2 years old (or >87 cm in height), whereas length (that is, lying down) was recorded for children under 2 years old or <87 cm. Weight was measured using Seca 877 scales. All measurements were taken in duplicate, with a third reading taken if the difference between the first two readings exceeded a set cutoff. The midpoint of duplicate measures was used. Further details are provided in Supplementary Method 5.

### Probability of adequacy calculations

To assess dietary adequacy, we calculated probability of adequacy (PA) values for 11 micronutrients of public health concern (calcium, zinc, vitamin C, thiamin (B1), riboflavin (B2), niacin (B3), vitamin B6, folate (B9), vitamin A, vitamin B12 and iron). Usual intakes were estimated as the mean of all available 24-h recalls for each individual. Participants were surveyed fortnightly over a full year, which provided up to 24 recalls for each participant. With this number of repeated measures, the influence of day-to-day variation on mean intake estimates is likely to be small, and the mean was therefore considered an acceptable approximation of usual intake^49^. PA values were derived using the standard probability approach^50^, with estimated average requirement values applied according to age, sex and physiological status (pregnancy, lactation, pre-menarche and post-menarche). Iron was treated separately owing to its non-normal requirement distribution, and low bioavailability assumptions (low absorption by the body) were applied for iron (5%) and zinc (15%) following previously described methods^51^. Further details are provided in Supplementary Method 6.

### Plant–pollinator visitation surveys

To characterize the plant–pollinator interaction network that underpins the delivery of the pollination service, we conducted plant–pollinator visitation surveys every 2 weeks from 18 April to 4 November 2021 (spring to autumn). These surveys immediately preceded the dietary-recall surveys but were not synchronous owing to COVID-19 restrictions, which prevented in-person interviews at the time of the ecological surveys. However, most of the crop produce that was grown and pollinated during the plant–pollinator surveys was consumed throughout the period of dietary-recall surveys. Moreover, agricultural production and diets remain similar from year to year in this region (Supplementary Table 12).

Plant–pollinator surveys were conducted in a 600 × 600 m (36 ha) sampling area centred on the midpoint of each study village. This area was divided into three habitat categories: village, crop and semi-natural vegetation. In each of these habitats, we randomly located three replicate fixed survey plots of 60 × 60 m (9 plots per village). Every 2 weeks, a timed 40-min survey was conducted for each plot to record the interactions between plants (both crop and non-crop species) and flower-visiting insects (those observed collecting resources from the flower). Surveys were conducted by a team of ten trained data collectors (recruited from within each village) using the cloud-based data-collection platform CommCare (v.2.49; http://www.commcarehq.org/home/). Plants were identified by data collectors using a custom-made Plant Atlas for Jumla District (with scannable QR codes to minimize transcription error), and species identifications were checked for accuracy by a botanist from the national herbarium of Nepal. All insect specimens were identified by insect taxonomists (see Acknowledgements for details) and are stored in the Central Department of Zoology, Tribhuvan University, Nepal. Insect identifications were checked for accuracy by an independent taxonomist from the University of Agricultural Sciences, Bangalore, India. Further details are provided in Supplementary Method 7. Sampling permission was provided by the Ministry of Forest and Environment, Nepal (reference 258).

### Estimating pollinator importance

Without detailed studies of the behaviour and morphology of each insect taxon, and on the seed set of plants exposed to different pollinators, we cannot know which flower visitors are truly pollinators^52^. Here we assumed that any insect that visits a flower is a potential pollinator and we estimated pollinator importance on the basis of its visitation frequency, which has been shown by both empirical^39^ and theoretical^53^ work to provide a good indication of ecosystem service delivery. However, visitation information alone can overstate the importance of abundant but less effective pollinator taxa such as butterflies^54^. Thus, to estimate the pollination service delivery of a pollinator taxon, we used a modified version of visitation frequency and weighted each visitation event by the pollen transport capacity of the insect (mean number of pollen grains from all plant species carried on its body, as per a previously described method^41^). This metric is based on the assumption that insects that frequently visit a focal crop and carry large amounts of pollen on their bodies are likely to be better pollinators than insects that visit less frequently and carry little pollen, consistent with previous studies^55,56^. This estimate of pollination service provision, although imperfect, is a necessary simplification for a large-scale study involving multiple plant and pollinator species across multiple sites. It will help identify dominant crop pollinators, even if the values are more relative than absolute. Pollen transport capacity was estimated by swabbing individuals of each insect taxon and calculating the mean number of pollen grains from all plant species carried on their body and available for pollination. Pollen grains were not identified to species (which is impractical for a study of this scale), so our measure of pollen-carrying capacity is limited to one value per insect taxon rather than specific values for each plant–pollinator combination (more details provided in Supplementary Method 8). For each insect, our pollinator importance (PI) metric estimates its proportional contribution to the total pollen transport for a given crop species based on the following equation:

$${{\rm{PI}}}_{i,c}=({V}_{i,c}\times {P}_{i})/\sum _{i}({V}_{i,c}\times {P}_{i})$$

where *V*_*i*,*c*_ is the visitation frequency of insect *i* to crop *c* and *P*_*i*_ is the pollen transport capacity of insect *i*. Thus, insects that carry a lot of pollen and make up a high proportion of all visits to a crop will receive a high importance score.

### Plant–pollinator interactions that underpin human nutrition

By combining participants’ dietary data with published nutrient composition values for each ingredient, we were able to determine the origin of each nutrient in participants’ diets (that is, the plants and animals it was sourced from). We then calculated the proportion of each nutrient obtained from local versus imported ingredients, as well as pollinator-dependent versus pollinator-independent ingredients (see the categorization process below). Once the source of each nutrient was established, we identified the insects that supported its production via their pollination of the nutrient-provisioning crops.

#### Categorizing foods and calculating their contribution to diets

Each individual food item or ingredient was classified as either locally derived (that is, grown or raised within the isolated district of Jumla) or imported (from outside Jumla) based on responses to the dietary-recall survey, which included a question on food provenance. The isolation of the study villages from regional markets made it relatively straightforward to determine which ingredients were imported; these were typically non-perishable packaged items such as polished white rice, vegetable oil and spices. Ingredients were also classified as either pollinator-dependent (production increased by animal pollination) or pollinator-independent (production not affected by animal pollinators) based on previous studies^11,46^. In our study system, farmers are rarely able to access or afford external seed sources; therefore, all households sow their crops with local seeds saved from the previous year (Supplementary Table 10). Thus, we consider crops as pollinator-dependent if their seed production relies on animal pollination, even if the edible portion of the crop does not (for example, carrots and mustard greens).

#### Calculating the contribution of insect taxa to diets

To provide an aggregate estimate of the contribution of each insect to human nutrition, we multiplied its PI score for a given crop (see the section above) by the proportional contribution of the crop to the supply of a given nutrient. To account for differences in pollinator dependence among crops, the PI scores for each crop were multiplied by the percentage pollinator dependence of the crop defined as the reduction in yield recorded when flower visitors are excluded (values were taken from the literature and our own pollinator exclusion experiments; Supplementary Table 4 and Supplementary Method 9). Thus, for a given insect (*i*), its pollinator contribution (PC) to the supply of a given nutrient (*n*) is calculated as:

$${{\rm{PC}}}_{i,n}=\sum _{c}({{\rm{PC}}}_{i,c}\times {D}_{c}\times {N}_{c,n})$$

where PI_*i*,*c*_ is the importance score of pollinator *i* for crop *c* (see the equation for PI_*i*,*c*_ above), *D*_*c*_ is the pollinator dependence of crop *c* and *N*_*c*,*n*_ is the proportional contribution of crop *c* to the nutrient of interest (*n*). Thus, an insect that carries lots of pollen and frequently visits pollinator-dependent crops with a high nutritional value will receive a high score. The score represents the proportion of nutrient *n* that is directly attributable to pollination by insect *i*.

### Pollination changes and how they affect human welfare

Understanding how declines in pollinator populations affect human health and livelihoods is challenging because direct long-term data are lacking. To address this issue, we used simulations to explore a range of plausible scenarios of pollinator population change and their consequences for diets and incomes. Although such simulations inevitably involve assumptions, their purpose is not to provide precise forecasts but to generate boundary estimates that illustrate the scale of potential impacts without any adaptation or mitigation responses. We combined crop–pollinator interaction data with dietary-recall data and household income data (recorded in questionnaires; Supplementary Method 10) to estimate the consequences of changes in the pollinator community. For each crop consumed by a participant, or sold by a household, we first calculated the contribution of individual pollinator taxa to its yield, based on the crop’s pollinator dependence and the proportion of pollen transport provided by that taxon (see above). This approach enabled us to estimate the portion of each participant’s nutrient intake and the proportion of each household’s farming income attributable to each pollinator species and to alter it according to the simulations described below.

#### Simulating declines in pollinators

We modelled declines in pollinator populations by simulating stepwise reductions in pollinator abundance from 0 to 100% loss in 10% increments. For each decline level, every pollinator taxon was assigned a species-specific decline rate drawn from a normal distribution, with the target mean decline and a standard deviation of 0.2, to reflect interspecific variation in sensitivity to environmental change. Rates were bounded between 0 and 100%. We then reduced each participant’s nutrient intake and each household’s farming income based on the share of their diet or income that was attributable to the pollinator in question. For each decline level, we repeated simulations 100 times to generate distributions of possible outcomes.

Although our analysis produced results across the full gradient of pollinator decline, in the main text, we focused on two scenarios that provide useful boundary estimates for clarity and policy relevance. Scenario 1 represents a complete loss of local pollinators, an extreme outcome that illustrates the maximum potential impact on nutrition and livelihoods. Scenario 2 represents a business-as-usual trajectory, derived from projected trends in the native honeybee *A. cerana* reported by beekeepers in our ten study villages^29^. In the absence of long-term pollinator monitoring data, we used this projection (33% decline from 2021 to 2030) as a proxy for all pollinator taxa, with the notion that locally reported drivers (climate change and floral resource loss) are equally likely to affect the abundance of wild pollinators^57,58^ as for the abundance of *A. cerana* (details in Supplementary Method 11). Although necessarily a simplification, the rate is consistent with modelled estimates for declines in wild insect elsewhere^24^ and may even be conservative given that wild pollinators typically decline faster than managed honeybees^59,60^.

#### Simulating pollination recovery

Our third modelled scenario aimed to predict the potential nutritional and economic gains if pollination services were actively managed to close the proportion of each crop’s yield gap that is attributed to insufficient pollination (that is, achieving optimal pollination). We first calculated current yield gaps for four key pollinator-dependent crops (Jumli beans (*Phaseolus vulgaris*), mustard (*Sinapis alba*), pumpkin (*Cucurbita maxima*) and apple (*Malus domestica*)) that together account for >75% of pollinator-dependent food consumption and >90% of farming income. Yield gaps were defined as the difference between the upper-95 percentile yield and the median yield across farms in our study region. We then assumed that a part of this yield gap (median of 24%) could be closed, drawing on data from a global study of comparable smallholder farms in Asia (including Nepal), Africa and Latin America that quantified the fraction of yield gaps attributable to insufficient pollination^36^. Notably, we only closed this pollination-related share of the yield gap, whereas the larger proportion caused by other agronomic constraints (for example, fertilizer, water and soil quality) was left unchanged. Following a previously described approach^17^, we applied this pollination-related yield increase to each crop, recalculated participants’ nutrient intakes and household incomes and compared these to current values (further details in Supplementary Method 12). Our pollination deficit estimates aligned closely with those reported in regional^61^ and global^30^ assessments, which provided confidence in the validity of this scenario.

#### Key model assumptions

All simulations were based on the following simplifying but evidence-informed assumptions: (1) the pollinator-dependent yield of each crop declines in proportion to the decline in pollinator abundance, as per a previous study^24^; (2) only the yields of locally produced pollinator-dependent crops are affected (representing the part of the food system under farmers’ control), whereas imported foods are assumed unchanged; (3) changes in food production lead to equivalent changes in food consumption, as this is a strongly food-limited population for which production and consumption are tightly linked (indicated by the strong seasonal synchrony between production and consumption of micronutrient-rich foods; Supplementary Fig. 7); (4) food lost from the diet is replaced by the main staple crop rice (of an equal number of calories), as this is one of the few staple foods routinely imported and affordable year-round and typically substitutes for local ingredients when unavailable; (5) declines in locally produced pollinator-dependent foods are not offset by imports, as market failures and geographical isolation strongly constrain their availability and affordability^62^ (Supplementary Fig. 17); (6) the market price for exported cash crops (for example, apples and beans) remains fixed, as prices are mostly determined by supply and demand in larger regional markets rather than local production changes; and (7) farmers do not alter cropping patterns in response to declines in pollinator-driven yields because high investment costs and low financial capital limit their ability to adapt. Further details and sensitivity analyses are provided in Supplementary Method 1. Although these assumptions do not capture the full complexity of food systems, they are not improbable in the context of Jumla District. By making the assumptions explicit, we highlight where interventions could mitigate these effects, for example, by strengthening market access to improve the availability of micronutrient-rich foods or by providing economic safety nets that enable farmers to adapt to changing conditions.

### Pollinator characteristics that make them nutritionally important

To extend the generality of our findings beyond the specific pollinator taxa present in our study system, we investigated the network characteristics of pollinators that could predict their nutritional importance. We quantified the nutritional importance of a pollinator as the proportional reduction in the intake of six key pollinator-dependent nutrients (vitamin A, folate, vitamin E, calcium, iron and vitamin C) across the entire study population when the pollinator is removed from the crop–pollinator network (same modelling framework as described above). We then tested whether variation in nutritional importance among pollinators could be explained by their abundance and a limited set of species-level network descriptors calculated on the full plant–pollinator network (all villages combined) using the R packages bipartite^63^ and vegan^64^. Metric selection was based on a previous study^65^ and aimed to represent a small number of complementary dimensions of pollinator behaviour and network position that could plausibly influence crop pollination and nutrient provision while remaining relatively straightforward to interpret for agricultural and nutrition specialists and network ecologists.

#### Network metrics tested

Pollinator abundance was quantified as the total number of flower visits recorded for a species across all plants, which provided a proxy for its local pollination activity. Abundance was included as a baseline predictor in all models as it is a fundamental ecological property that strongly influences both network structure and function^65,66^.

To capture how pollinator activity is allocated between crops and non-crops, we calculated a metric we term ‘crop focus’, which was defined as the proportion of total visits by a pollinator (across all plants) that were directed to crop species. This metric distinguishes pollinators that concentrate their activity on crops from those that primarily visit wild plants, independent of overall activity level.

In addition to pollinator abundance, we characterized the interaction breadth and selectivity of each insect using three complementary metrics: (1) degree, which captures partner range (the number of plant species visited) and is interpreted strictly as a measure of interaction breadth rather than interaction strength^67^; (2) Shannon diversity of interactions, which captures how evenly a pollinator distributes its visits across its plant partners, weighting partners by visitation frequency^68^; and (3) Blüthgen’s *d*′, a specialization metric that quantifies how selectively a pollinator uses its plant partners relative to their availability in the network^69^. Together, these metrics describe how many plant species an insect visits, how evenly it distributes its visits among those plants and whether its interaction pattern reflects opportunistic use of available resources or clear preferences for particular plant species^70^. We expected that insects with high degree and interaction diversity are nutritionally important pollinators because they interact with a wide range of crops. By contrast, insects with higher *d*′ values may be particularly important for maintaining pollination of key crops owing to their consistent and selective visitation patterns.

To describe the position of each insect in the network as a whole, we calculated weighted closeness centrality, which was defined as the average distance of a pollinator to all other species in the network^71^. Closeness centrality is commonly interpreted as a proxy for the potential of a species to influence network-wide dynamics, which is based on the assumption that direct interactions exert stronger effects than indirect ones and that the strength of indirect effects declines as the number interaction steps increases^71^. In this context, centrally positioned pollinators have the potential to affect pollination across multiple crops in the network, although high centrality does not necessarily imply strong or frequent interactions with crop plants.

Because network metrics are influenced by abundance and sampling effects^65^, we converted each observed network metric to a *Z* score based on 99 null-model simulations that preserved the total number of visits per pollinator while randomizing their distribution across plant species. Null models were generated using the nullmodel function in the R package vegan with the c0_ind method^64^. These *Z* scores indicated whether a pollinator exhibits higher or lower values of a given network property than expected based on its abundance alone. We then used linear models to test whether pollinator characteristics predict nutritional importance, including abundance as a baseline predictor and testing each *Z*-standardized network metric for additional explanatory power. Model performance was assessed using changes in Akaike information criterion, adjusted *R*^2^ and the significance of regression coefficients.

### Management of pollinators to enhance human nutrition

One of the most successful and widely practised pollination management strategies is the addition of wild flowering plants to farmland to provide food for pollinators^43,44^. Focusing our attention on this common pollination-management approach, we predicted the most important non-crop plants for supporting the key crop pollinators at our field sites. We estimated this ‘indirect contribution’ of each wild plant to the pollination service by calculating its visitation by insects and weighting the visitation of each insect by its PC score (see section above). Because PC is calculated separately for each nutrient, we sum values across all nutrients to obtain an overall contribution for insect (PC_*i*_). Thus, for a wild plant *p*, our estimate of its indirect contribution (IC) to pollination services is calculated as follows:

$${{\rm{IC}}}_{p}=\times \sum _{i}({{\rm{PC}}}_{i}\times {V}_{i,p})$$

where PC_*i*_ is derived from the equation in the section described above and *V*_*i*,*p*_ is the visitation frequency of each insect *i* to plant *p*.

Thus, a wild plant that is frequently visited by pollinators of crops that provide a large proportion of peoples’ micronutrient supply will receive a high IC score. This approach assumes that the value of a plant to pollinators is proportional to how often it is visited, without accounting for the nutritional quality of floral resources or how much is consumed during visits. Our estimates should therefore be interpreted as indicative rather than precise. To validate this approach, we investigated the phenological pattern of crop and wild plant visitation to check that wild plants are supporting crop pollinators with floral resources outside the main periods of crop flowering and therefore are facilitating crop pollination rather than simply competing with crops for pollinators (Supplementary Fig. 16).

It should be noted that in addition to the high-scoring plants identified through this metric, there may be some less widely used (and therefore lower scoring) plants that fill temporal or nutritional gaps in food supply and are therefore more important than their score would suggest. For the purposes of this analysis, however, we focus on the most widely used plants that are assumed to provide insects with most of their key resources.

### Data analysis

Data cleaning and processing were conducted using STATA (v.18), whereas all data analyses, modelling and figure plotting were performed in R (v.4.4.2, R Core Team).

### Reporting summary

Further information on research design is available in the Nature Portfolio Reporting Summary linked to this article.

## Online content

Any methods, additional references, Nature Portfolio reporting summaries, source data, extended data, supplementary information, acknowledgements, peer review information; details of author contributions and competing interests; and statements of data and code availability are available at 10.1038/s41586-026-10421-x.

## Supplementary information

Supplementary Information This Supplementary Information file contains 12 Supplementary Method sections detailing the study design, dietary and pollinator methods, modelling assumptions and economic analyses, alongside 12 Supplementary Tables and 17 Supplementary Figures presenting additional results such as model outputs, and additional analyses supporting relationships between pollination services, nutrition and livelihoods.

Reporting Summary

Peer Review file

## Data Availability

The datasets generated and analysed in this study are publicly available in the NERC Environmental Information Data Centre (EIDC) (10.5285/d7434d83-c30d-4186-aab0-9764821cd807)^72^. This repository contains the processed datasets supporting the analyses and figures presented in this study, together with associated metadata. Plant identification during field data collection was supported using the custom-made Plant Atlas for Jumla District, which is publicly available (https://herdint.com/resources/jumla-plant-atlas/).

## Code availability

All R scripts used for data processing, statistical analyses, simulations, network analyses and figure generation in this study, together with documentation and source data required to reproduce the results, are publicly available at Zenodo (10.5281/zenodo.18838606)^73^.
