# Supplementary material for: Pollinators support the nutrition and income of vulnerable communities
Source: Nature. 2026 May 6;654(8119):683–8. doi: 10.1038/s41586-026-10421-x (PMC13275292; doi:10.1038/s41586-026-10421-x)
Supplement: Supplementary file 1 — This Supplementary Information file contains 12 Supplementary Method sections detailing the study design, dietary and pollinator methods, modelling assumptions and economic analyses, alongside 12 Supplementary Tables and 17 Supplementary Figures presenting additional results such as model outputs, and additional analyses supporting relationships between pollination services, nutrition and livelihoods. [file 41586_2026_10421_MOESM1_ESM.pdf]

nature portfolio

https://doi.org/10.1038/s41586-026-10421-x

Supplementary information

Pollinators support the nutrition and income of vulnerable communities

In the format provided by the authors and unedited

Pollinators support the nutrition and income of vulnerable communities

Authors:

T.P. Timberlake [1, 2 *], S. Sapkota [3], N.M. Saville [4], A.R. Cirtwill [5, 6], S.C. Baral [3], D.R. Bhusal [7], K. Devkota [8], S. Giri [3], H.A. Harris-Fry [9], D. Joshi [3], S. Kortsch [5, 10], S.S. Myers [11, 12, 13], T. Roslin [5], M.R. Smith [13, 14], J. Memmott [1 *]

Affiliations:

[1] School of Biological Sciences, University of Bristol; Bristol, UK.

[2] Department of Environment and Geography, University of York; York, UK

[3] HERD International; Kathmandu, Nepal.

[4] Institute for Global Health, University College London; London, UK.

[5] Department of Agricultural Sciences, University of Helsinki; Helsinki, Finland.

[6] Carex EcoLogics; Bracebridge, Canada.

[7] Central Department of Zoology, Tribhuvan University; Kathmandu, Nepal.

[8] Faculty of Agriculture, Agriculture and Forestry University; Chitwan, Nepal.

[9] Department of Population Health, London School of Hygiene & Tropical Medicine, London, UK.

[10] Tvärminne Zoological Station, Faculty of Biological and Environmental Sciences, University of Helsinki; Hanko, Finland.

[11] Department of Environmental Health and Engineering, Johns Hopkins Bloomberg School of Public Health; Baltimore, USA.

[12] Johns Hopkins Institute for Planetary Health; Baltimore, USA.

[13] Department of Environmental Health, Harvard T.H. Chan School of Public Health, Boston, USA.

[14] Department of Environmental and Occupational Health Sciences, School of Public Health, University of Washington, Seattle, WA, USA

* Correspondence authors: thomas.timberlake@bristol.ac.uk; jane.memmott@bristol.ac.uk

Contents

Supplementary Methods 4

Supplementary Method S1. Justification and sensitivity analysis for model assumptions 4

Supplementary Method S2. Additional background on the study region (Jumla Nepal) 9

Supplementary Method S3. Study population and dietary recall survey methods 10

Supplementary Method S4. Recipe collection methods 13

Supplementary Method S5. Height and weight recording methods 15

Supplementary Method S6. Calculation of Probability of Adequacy 16

Supplementary Method S7. Plant-pollinator visitation survey methods 18

Supplementary Method S8. Pollen quantification: methods and caveats 19

Supplementary Method S9. Pollinator exclusion experiments 21

Supplementary Method S10. Farmer questionnaire surveys for economic assessments 23

Supplementary Method S11. Estimating local pollinator declines: rationale and caveats 24

Supplementary Method S12. Predicting yield increases from optimum pollination 26

Supporting Tables 28

Table S1. Crops recorded in Jumla study sites. 28

Table S2. List of key pollinator-dependent crops cultivated in Jumla 30

Table S3. Pollinator Contribution (PC) scores of each insect for each key micronutrient 31

Table S4. Food item contributions to key micronutrients 32

Table S5. Nutritional status of study population 33

Table S6. Nutrient intake, requirements and probability of adequacy scores 34

Table S7. Predicted changes in nutrient intake resulting from changes in pollination service 35

Table S8. Probability of adequacy by population subgroup and pollination scenario. 36

Table S9. Summary of models relating species network metrics to nutritional importance 37

Table S10. Non-crop plant species Indirect Contributions (IC) to each key micronutrient 38

Table S11. Characteristics of the study population in Jumla, Nepal. 39

Table S12. Annual production of key crops and crop groups in Jumla District 40

Supporting figures 41

Figure S1. Comparing smallholder farmers in Jumla with other regions of the world 41

Figure S2. Map of study village locations in Patarasi Rural Municipality, Jumla 42

Figure S3. Habitat composition of study villages 43

Figure S4: Photographs of study sites 44

Figure S5. Village-level importance of crops, pollinators and wild plants 46

Figure S6. Source of key food groups 47

Figure S7. Seasonal patterns in mean daily intake of major food groups 48

Figure S8. Pollinator dependence of key nutrients by population subgroup 49

Figure S9. Contribution of different foods to intake of key micronutrients 51

Figure S10. Comparing reliance on pollinator-dependent crops with other countries 52
Figure S11. Simulated impacts of pollination decline on nutrient intake 53
Figure S12. Village-level changes in micronutrient intake and farming income 54
Figure S13. Predicted declines in nutrient intake by population subgroup 55
Figure S14. Relationship between pollinator abundance and nutritional importance 56
Figure S15. Correlations amongst species-level network metrics 57
Figure S16. Seasonal patterns of crop and wild plant visitation 58
Figure S17. Proportion of household crop production consumed by household members 59
References 60

Supplementary Methods

Supplementary Method S1. Justification and sensitivity analysis for model assumptions

In our analysis of the impacts of pollination changes on human nutrition and livelihoods, we make several simplifying but evidence-informed assumptions. We outline each of these assumptions below and provide justification (and potential caveats) for each one, as well as testing the sensitivity of key results to the assumption, where relevant.

<u>Assumption 1: Crop yields decline in proportion to pollinator decline</u>

The pollinator-dependent yield of each crop is assumed to decline in proportion to the decline in pollinator abundance, following (Millard *et al.* 2023). This assumes a linear relationship between pollinator abundance and pollination service provision, which is feasible in a pollination-limited system such as ours and supported by modelled and empirical evidence from other studies (Lonsdorf *et al.* 2009; Dainese *et al.* 2019; Woodcock *et al.* 2019).

<u>Assumption 2: only local pollinator-dependent foods are impacted</u>

Here, we make the assumption that only the yields of locally-grown pollinator-dependent crops are impacted by pollinator declines, whilst any imported pollinator-dependent foods (most notably soybean oil and sunflower oil) remain unaffected by our model scenarios. We make this assumption for the following key reasons: a) we can measure or estimate local trends in pollinator populations and pollination services with some level of accuracy, whereas global pollinator trends are much less clear and likely to vary substantially between regions (Millard *et al.* 2023); b) the availability of imported foods is heavily dependent on a range of factors beyond the scope of this study including global supply chains and levels of demand in other regions of the world; c) we were interested in the direct links between local insect pollinators, crops and human nutrition, over which farmers have some level of control, rather than the more intangible reliance on pollinators in other regions of the world which local farmers cannot influence.

To test the influence of this assumption on our results, we repeated all analyses making the alternate assumption that the availability of imported pollinator-dependent foods changes in the same way as local pollinator-dependent foods. This assumes that pollinator declines are occurring across the world at approximately the same rate as in our local region and no other market factors change their availability. The results of this sensitivity analysis (Table S7) demonstrate that changes in the availability of imported pollinator-dependent foods have very little impact on people's diets, except in the case of vitamin E which is primarily obtained from imported pollinator-dependent soybean and

sunflower oil. This underscores the immense importance of local pollination services which farmers have at least some level of control over.

Assumption 3: Changes in food availability lead to equivalent changes in food consumption

Here, we make the assumption that when the availability of a given crop changes, people's consumption of that crop changes by an equivalent percentage. For example, if the yield of beans declines by 10%, we assume that people's consumption of beans also declines by 10%. While this proportionality assumption may not hold in well-integrated food systems with strong market mechanisms, we believe it is justified in Jumla for several reasons: a) This is a strongly food-limited population with low nutrient adequacy (Table S5), so households are likely to consume as much as is available to them; b) Market channels are extremely limited in Jumla, meaning most production is consumed directly within households. In many settings, production surpluses can be absorbed through exports, processing, or sale, weakening the link between production and consumption. By contrast, Jumla's geographic isolation, poor infrastructure (e.g. lack of storage, transport, and processing facilities), and low purchasing power create a highly constrained food system. Our household survey data show that over 85% of crop production is consumed directly by the producing household, with very little sold, traded, gifted, or fed to animals (Fig. S17). This reliance on own production also insulates households from price shocks in perishable goods. Under these circumstances, changes in production are much more likely to translate directly into changes in consumption, making the proportionality assumption more realistic than in more market-integrated contexts; c) Jumla's geographic isolation and weak market integration also limits opportunities for consumer substitution: more than 90% of perishable, micronutrient-rich foods (fruits, vegetables, leafy greens, pulses, and animal-source foods) are locally produced, while imported staples such as rice and oil are nutritionally distinct and cannot replace them (Fig. S6); d) Seasonal analyses show strong synchrony between production and consumption: intakes of key food groups and associated micronutrients rise sharply after harvest and decline as local availability falls, often varying several-fold across the year (Fig. S7). This pattern strongly suggests unmet demand and reinforces the idea that consumption is constrained by local production; e) Wealth disparities within the study population are minimal, reducing the likelihood of divergent consumption responses across households. Together, these factors support the assumption that, in this context, proportional changes in production provide a reasonable approximation of proportional changes in consumption.

Assumption 4: Food lost from the diet is replaced by rice of an equal number of calories

Here, we make the assumption that when the yield of a pollinator-dependent crop decreases, participants reduce their intake of that crop accordingly but maintain their caloric intake by increasing

their intake of imported white rice. We make this assumption for the following key reasons: a) participants are expected to satiate their hunger (resulting from the loss of calories) with alternative foods that are readily available, following (Smith *et al.* 2015); b) imported white rice is the main staple food in this region (making up >35% of people's total food intake, by mass) and is relatively affordable, non-perishable and available to purchase year-round, so is the most likely candidate for replacing lost calories; c) participants are unlikely to replace pollinator-dependent fruits and vegetables with directly equivalent foods (i.e. other fruits and vegetables) because there are very few non-pollinator dependent fruits and vegetables grown in Jumla and almost zero intake of imported fruits and vegetables (Fig. S6); this being due to geographic isolation which makes the purchase of perishable imported foods unaffordable.

To test the influence of this assumption on our results, we repeated all analyses making the alternate assumption that instead of replacing lost food intake with rice of an equal number of calories, we instead replace it with potatoes of an equal number of calories. Whilst not as prevalent as rice, potatoes are nevertheless widely grown, non-pollinator-dependent and available much of the year. The results of the sensitivity test (Table S7) reveal slightly less severe impacts on people's nutrition when lost calories are replaced with potatoes instead of rice; this is because potatoes have a higher density of many micronutrients than white rice. This was particularly true for vitamin C (which potatoes are rich in), resulting in a net gain in this nutrient when it substitutes for other foods.

Assumption 5: Local pollinator-dependent fruits and vegetables are not substituted by imports

Here, we make the assumption that when the availability of local pollinator-dependent fruits and vegetables decreases, people do not substitute them with equivalent imported foods. We make this assumption for the following key reasons: a) like many other smallholder communities across the world, Jumla District is remote and suffers from poor transport links resulting in very high additional costs on the import or export of perishable items such as fruits and vegetables; b) smallholder farmers (including those in Jumla) have very limited financial resources and thus cannot afford the high costs of imported fruits and vegetables; c) as a result of these two factors we see extremely low intakes of imported fruits and vegetables by participants in our study (Fig. S6 showing <2% of fruits and vegetables consumed are imported); d) analysis of food intakes through the year shows that intake of fruits and vegetables decreases markedly during the off-season, demonstrating that trade and imports are not substituting for local production (Fig. S7).

Under an alternative economic and geographic scenario in which imported fruits and vegetables becomes entirely accessible and affordable for smallholder farmers, we would expect to see a much lower impact of local pollinator declines on people's diets. However, whilst the region remains poor and isolated this scenario is extremely unlikely.

Assumption 6: Market prices for exported cash crops remain unchanged

Here, we make the assumption that market prices obtained by farmers for the sale of their cash crops (primarily apples and beans) are unaffected by changes in local production. For example, if local apple production declines due to reduced pollination services, we do not expect the market price of exported apples to increase substantially and buffer the impact on farming income. The primary reason for this assumption is that Jumla district is a very minor supplier in a much wider regional and global market. For example, most apples are exported to regional markets in the capital Kathmandu, as well as in India where they make up a small percentage of the total market share (Thapa *et al.* 2024). Thus, the market price of cash crops will be largely determined by factors outside of the region such as consumer demand and the total supply of this crop across the region, including from much larger suppliers such as China. Whilst there are known to be fluctuations in market prices of cash crops from year to year (Thapa *et al.* 2024), these are not simply driven by changes in local supply and it is therefore not possible to accurately predict how market prices will change in response to the different scenarios of cash crop yield in our model.

Assumption 7: Cash crop farmers do not switch crops in response to yield declines

Here, we make the assumption that if the yields of pollinator-dependent cash crops fall (in response to pollinator declines), farmers will not simply switch to alternative non-pollinator dependent crops to maintain their farming income. We make this assumption for the following reasons: a) almost all of the cash crops currently grown in this region are pollinator-dependent (pollinator-dependent crops make up 93% of farming income), so there are limited alternative options available to farmers; b) as in many other regions of the world, switching to new crops often entails high investment costs such as the purchase of new seeds, rootstock or equipment which is likely to be prohibitively expensive for resource-constrained smallholder farmers, especially given that many cash crops are slow-growing trees or shrubs; c) most farming income in this region is derived from apple orchards which have high investment costs including the purchase of the seedlings/rootstock and long payback periods before they start to bear fruit, thus switching crops entails a large financial sacrifice; d) yield declines are predicted to occur gradually and incrementally rather than suddenly, so there is unlikely to be a sudden event which precipitates farmers to switch crops; e) farming traditions are strong in many smallholder communities and local knowledge is often linked to specific crops that have been grown by the community for decades, thus switching crops will often require the accumulation of new knowledge and traditions.

An alternative scenario is that smallholders abandon farming completely in the face of yield declines, instead switching to new occupations such as manual labour to maintain their household income. This shift has occurred in many smallholder communities across South Asia and Africa, however it often requires a large financial outlay (for example the cost of re-training or migrating to where work is available) and is therefore not an option for the poorest and most vulnerable populations. Moreover, these alternative livelihoods (particularly migrant labour) have been associated with various negative outcomes including worker exploitation and the disruption of families and communities, as well as reduced local food security and sovereignty.

Supplementary Method S2. Additional background on the study region (Jumla Nepal)

The study took place in the remote mountainous district of Jumla, situated in the Karnali Province of western Nepal (Fig. S2). Rates of poverty, food insecurity and malnutrition are particularly high in this region, and 80% of the population are directly dependent on smallholder agriculture (Ministry of Health and Population 2018). More than 50 crops are grown in this region, including many pollinator-dependent species such as apples, beans, pumpkins, mustard, and buckwheat (Table S1 & S2). Jumla was historically isolated from national markets, so farming was primarily for subsistence purposes and the food and nutrition security of households were entirely dependent on their agriculture (Gaire *et al.* 2015). However, in the last 10-20 years, improved infrastructure has provided access to national and international markets, providing new commercial farming opportunities. Although feeding their families remains the primary agricultural goal for most farmers in the region, some have transitioned fully or partially towards commercial cash crop farming. This transition is ongoing, incentivised by the national government (Gaire *et al.* 2015). The high diversity of pollinator-dependent crops grown in Jumla (Table S2), combined with changing farming priorities and a dearth of information on local pollinator communities, has made it challenging to design pollination management strategies for farmers. Within Jumla District, our study took place in Patarasi Rural Municipality – one of eight municipalities in Jumla, with a population of approximately 15,000 people. Patarasi is typical of the rest of Jumla, though is more rugged and isolated than many other municipalities, with higher rates of poverty, malnutrition, and food insecurity.

Supplementary Method S3. Study population and dietary recall survey methods

Selection of study villages and households

The ten study villages were purposively selected using satellite imagery and discussion with a local key informant who has worked for many years in Patarasi rural municipality, Jumla. Selection criteria were that the villages should be: accessible (within 1 day's walking distance from Jumla Bazar); medium sized villages (30-200 households); located in the mid-altitude zone between 2300 and 2800 meters of Jumla district where fruit and vegetable production is common; and be typical of the wider district in terms of caste, ethnicity, livelihoods and farming practices. Within each of these study villages, we randomly selected 20 study households from within a pool of eligible households (determined through a census of each household within each village). Households were considered eligible for study participation if they: a) consumed at least some food grown by themselves or by others in the village; b) had at least three of the four target population subgroups present (see groups below) and c) were permanent residents in the community (spent at least 10 months/year in the village). Ethical approval was provided by the Nepal Health Research Council [Ref: 1709].

Selection of study participants

Individuals within a household were excluded from participating in the study if they: a) Had some disability which prevented them from responding clearly to the dietary recall survey or from understanding the questions. b) Had an ongoing medical condition which caused them to change or restrict their consumption of certain foods. This included diabetes (type 1 and 2), gout, cancer, or any liver or kidney disorder. c) Are only a temporary resident of the village (spend more than two months of the year outside the village, for example on migrant labour).

From each of the 200 study households, we surveyed the following eligible people: 1) adult woman of child-bearing age (20-48 years at enrolment, 21 to 49 by the end of the study), 2) adult male of 20-48 years (the husband of the adult woman, or the household head if they are not available), 3) unmarried or married adolescent girl (10-18 years at enrolment, 11 to 19 years by study end) 4) young child (6-47 months at enrolment, 18 to 59 months at end of the data collection). We selected these population subgroups as groups 1, 3 and 4 are the most vulnerable to micronutrient deficiency whilst group 2 enabled us to investigate differences between male and female adults. If more than one individual from within each subgroup was present in the household, a random selection process was used to determine which individual was surveyed. If a study household contained only three of the above subgroups, an extra person (where possible) was sampled from a study household in the same village with additional members within this same subgroup to ensure a balanced design. In most cases there was only one individual per respondent category per household. However, due to a shortage of

adolescent girls and children in the right age categories we enrolled more than one per household of these categories where available (of the enrolled households 33 had two adolescent girls, five had three adolescent girls, and 25 households had two children). Also due to the need for replacement of family members that migrated out, in seven of the households we enrolled two women, in four households we enrolled two men, and in one household we enrolled three women.

Dietary recall surveys

Each participant was visited twice per month (every 2 weeks) for a 24-hour dietary recall survey describing all the foods consumed from when they got up in the morning the previous day until the same time on the day of recall. If a household member was away from home all the previous day and night their intakes were not recorded. Where available and with the consent of parents/guardians, participants over 13 years (considered old enough to understand questions and remember what they have eaten) were asked to respond on their own behalf, but for young children, the caregiver and/or cook was asked to recall intakes on their behalf. For adolescent girls younger than 13 years, the household cook provided responses in their presence. If the adolescent was absent, the dietary recall was not administered. In this region, occupants of a household typically eat food that is prepared in the same cooking vessel and therefore comprised of exactly the same ingredients.

To capture accurate recall of dietary intake, we adopted a 5-stage multi-pass survey method (Harris-Fry *et al.* 2018), as follows: (1) collect a chronological free recall of all foods consumed in the last 24 hours, (2) ask for the time and place of consumption, (3) ask about commonly forgotten foods like tea and fruit, (4) review information so far and probe for anything missing, and (5) collect detail on specific food names and measure portion sizes. Interviewers entered information from the first four stages onto a simple paper form to enable fluid interviewer-interviewee interactions, then for the fifth stage (food names and portion sizes) plus the time and place of consumption, they entered the information into the electronic survey form using a tablet. Interviews were conducted by trained data collectors in the Nepali language and information was entered into a customised data collection form using the cloud-based data collection platform CommCare (Version 2.49, http://www.commcarehq.org/ home/) on an Android tablet.

Food models were adopted to estimate portion sizes using *cooked rice* (with green food colour added to prevent consumption) as a model for rice and other irregular-sized solid foods such as vegetable curries and cooked green leaves; *water* for portions of dal, milk, or other liquids; *play-dough (modeling clay)* for chunks of meat or pieces of fruit; *wheat flour* for powdered foods like 'sattu' (roasted ground grains), powdered mixed salt chili and spices; and *dried corn* for dried or roasted nuts and grains. Where necessary, a combination of two models were used to show the liquid and solid parts of the dish, (e.g. for meat dishes with gravy or soup). Food models were weighed using Salter

kitchen scales and then back-converted to estimate the mass of each food item consumed, accounting for differences in food density. For foods in packets, or of a standard size, the number of items consumed was recorded. For each eating occasion and each food item consumed, the data collector measured out and weighed the portion size as instructed by the respondent to estimate the amount consumed. Participants were reminded to only report the food that was consumed, not any that was leftover on the plate. The process was repeated for each eating occasion to ensure that no foods were missed. After the completion of one household member the process was repeated on the other enrolled members of the household in turn.

Quality control

The CommCare data collection forms had range checks and internal validity checks built in to help maintain quality control. To ensure standard methods were employed and quality maintained, three supervisors made visits to the data collectors to observe data collection and advise where adjustments were needed. Monthly meetings were held for data collectors to discuss any issues arising and to enable the supervisor to brief and retrain as needed. Data collectors made notes in their diaries and logged any mistakes that needed correcting in the data as they were not able to amend electronic forms. Amendment lists and notes were shared with the supervisor at monthly meetings and problems addressed by telephone as and when needed. The supervisor checked these logs as well as summaries of data coming in on the CommCare platform and discussed corrections needed with the data collectors. These data corrections were handed on to the HERD Data Management team who made corrections in R script and/or STATA ‘do files’.

Supplementary Method S4. Recipe collection methods

To identify the composition of each mixed dish consumed (i.e. the quantity of each ingredient present in it), we asked the lead cook in replicate local households to prepare the food following their normal recipe. As it was not possible to collect the ingredients and weights of the ingredients in every dish on every recall occasion, standard recipes were collected by data collectors with the cooks of the households in our sample. Occasionally other community members also assisted by participating in standard recipe-making. To reduce chances of bias, data collectors were not permitted to eat the dish after it was cooked and asked respondents to make the dish as they usually would and not to make it 'special' in any way.

For each recipe collected, the empty cooking pot was weighed and then all the raw ingredients were weighed before adding to the dish. In the case of vegetables, the peeled, clean portion was weighed before cooking, unless the recipe called for something to be pre-boiled (as was the case for certain potato dishes). After all ingredients were weighed and the recipe was completely cooked, the weight of the entire cooked dish in the cooking pot weighed earlier was taken to estimate the weight of the compete dish. To 'calibrate' the food models, a portion of each of the standard dishes prepared was weighed in a standard size bowl and recorded. Then the equivalent volume of the food model for that food was weighed and recorded. For packet foods and foods recorded by number of items an average weight of that food was taken. Generally, the recipe was collected in the same village from which it had been consumed. The number of replicate recipes collected was determined by how frequently the food was collected and varied between 1 and 23 replicates with more frequent recipes having more replicates. For a small number of unusual items, recipes could not be recorded in the community; for these, recipes were collected by HERD researchers in Jumla bazaar or Kathmandu.

Recipe composition calculations

After all replicate recipes had been collected, data were cleaned and processed in STATA by summing ingredients across all replicates to get an average recipe. Ingredients were summed rather than averaged to enable all ingredients to be equally weighted, even if they were rarely used. After calculating the standard recipe, the amount of each ingredient per 100g of the cooked dish was calculated and this was merged with food composition data to provide the nutrient composition of each dish. For analyses where the average recipe content is needed, the sum of all the recipe ingredients was converted to nutrient value per 100g and added to the food composition table.

Food composition data sources

Our Nepal-specific food composition table (FTC) was compiled from a range of data sources following a hierarchical decision-making process whereby values from a nearby region were prioritised where available. Where possible, the Indian FCT (Longvah *et al.* 2017) was used, followed by the Bangladesh FCT (Shaheen *et al.* 2013), USDA SR25 (U.S. Department of Agriculture 2013), McCance and Widdowson's Composition of Foods (Public Health England 2021) and the Nepal FCT (Government of Nepal 2012). Owing to errors and incomplete information in the Nepal FCT, this source was only used for Nepali-specific foods that could not be found in other FCTs. If values for specific nutrients (e.g. B12) were missing from the FCTs of India or Bangladesh, values were added from equivalent foods from the USDA (SR25) or McCance and Widdowson's Composition of Foods. For a small number of packaged foods available in local shops, nutrient information from the back of the packet was used; however, for most packaged items, equivalent foods from existing FCTs were selected. For foods that did not have FCT values, searches of peer reviewed literature and grey literature were undertaken to find any available estimates. For wild and local foods that could not be located in any of the FCTs, values were taken for the closest equivalent items from whichever FCT provided the best match. Full details of the FCT data source for each ingredient are shown in the original data file (Memmott *et al.* 2024).

<u>Nutrient retention factors</u>

Because cooking can reduce vitamin and mineral levels, we adjusted nutrient values using the USDA Table of Nutrient Retention Factors, Release 6 (U.S. Department of Agriculture 2007). This table provides standard estimates of how much of each nutrient remains after different cooking methods (e.g. boiling, frying, steaming). Ingredients cooked in different ways were treated as separate entries (e.g. boiled vs. fried potato) so that the correct adjustment could be applied. This ensured that the nutrient intakes we calculated reflected the foods in the form they were eaten.

Supplementary Method S5. Height and weight recording methods

Data collectors were trained to measure height, length and weight of respondents. A data collector standardisation exercise was undertaken with 10 women and 10 children at the end of the training course to ensure that the technical error of measurement and coefficient of reliability were acceptable. Standing height was taken using Shorr Board Stadiometers for adults, adolescents and children over 2 years (or 87 cm or more in height), and length (lying down) was taken for children under 2 or under 87 cm. Height/length was recorded to the nearest completed millimetre. Weight was measured using a Seca 877 scale which provides readings accurate to the nearest 100g. If the child was not happy or not cooperating on the scale, mothers or other caregivers held the child during measurement. As Jumla is a cold place for much of the year and caregivers would not permit removal of clothes, children were weighed in their clothes and a set of similar dry clothes was weighed to estimate the weight of the clothes worn on each occasion. All measures were taken in duplicate with a third reading taken if the difference between readings exceeded 0.1kg for weight or 0.4cm for height/length, as per algorithms set in the electronic data collection form. The average of the two closest replicate measures was used.

Anthropometric calculations

Anthropometric Z-scores for length/height-for-age (LAZ/HAZ), weight-for-age (WAZ), weight-for-height (WHZ/WLZ), and BMI-for-age (BMIZ) were calculated in Stata using the zanthro command, based on the World Health Organisation growth reference for children aged 0–59 months (WHO 2006) and the WHO (2007) reference for children and adolescents aged 5–19 years. Standard WHO data cleaning criteria were applied, excluding implausible Z-score values (LAZ/HAZ < –6 or > +6; WAZ < –5 or > +6; and WLZ/WHZ/BMIZ < –5 or > +5). Nutritional status was categorised using standard WHO cut-offs: stunting (HAZ or LAZ < –2 SD), wasting (WHZ, WLZ, or BMIZ < –2 SD), and underweight (WAZ < –2 SD).

Supplementary Method S6. Calculation of Probability of Adequacy

To estimate the adequacy of individual nutrient intakes, we calculated the probability of adequacy (PA) for 11 micronutrients of public health concern: calcium, zinc, vitamin C, thiamin (vitamin B1), riboflavin (vitamin B2), niacin (vitamin B3), vitamin B6, folate (vitamin B9), vitamin A, vitamin B12, and iron.

<u>Estimation of usual intake</u>

Participants were surveyed every two weeks throughout a 12-month period, providing 24 repeated 24-hour recalls per individual (with a small number of missed rounds for some individuals). The mean daily intake across all recalls was used as an estimate of each individual's usual intake. Statistical adjustment for within-person variation is generally necessary when only a few recalls are available (Carriquiry 2003), but with a large number of repeated measures, bias from day-to-day variability is substantially reduced, and the mean intake more closely approximates the true usual intake (Nyambose *et al.* 2002; Kipnis *et al.* 2009).

<u>Nutrient requirements</u>

The Estimated Average Requirement (EAR) represents the daily intake level of a nutrient estimated to meet the needs of half of the healthy individuals in a given group, and is commonly used to assess dietary adequacy at the population level. EARs differ based on a person's sex, age, and physiological status, therefore we divided participants into separate age/sex/physiology categories (e.g. pregnancy, lactation, pre- vs. post-menarche for adolescent girls) and calculated their probability of adequacy (PA) using the relevant EAR values. For women whose physiological status changed during the study period (e.g. non-pregnant at enrolment but pregnant part-way through), we calculated PA separately for each status period and then derived an overall annual PA by taking a weighted mean of these values according to the number of recalls contributed in each state.

We used WHO/FAO reference values for nutritional requirements of vitamin C, vitamin A, vitamin E, thiamin, riboflavin, niacin, vitamin B6, folate, and vitamin B12 (WHO/FAO 2001); Institute of Medicine reference values for calcium (Institute of Medicine 2011) and iron (Institute of Medicine 2001); and International Zinc Nutrition Consultative Group (IZiNCG) recommendations for zinc (Hotz 2007). Because iron requirements for non-pregnant women and men are not normally distributed, probability of adequacy (PA) values were calculated using a table of probabilities for different intake intervals, adapted from the IOM (Institute of Medicine 2001).

For both iron and zinc, low bioavailability diets were assumed, consistent with cereal-based diets high in phytate typical of the study area (WHO & FAO 2004). Low bioavailability refers to the limited

proportion of a nutrient that is absorbed and utilized by the body due to the presence of dietary inhibitors such as phytates, polyphenols, and fiber. This corresponds to an assumed iron bioavailability of 5% and zinc absorption of approximately 15%.

Probability approach

For nutrients with requirements assumed to follow a normal distribution (all except iron), probabilities of adequacy were calculated by first expressing each individual's usual intake relative to the Estimated Average Requirement (EAR) and its variability (i.e. as a Z-score). These Z-scores were then converted into probabilities using the standard normal distribution, which indicates the likelihood that an individual's intake meets or exceeds their requirement (National Research Council 1986). Iron was treated differently because requirements are not normally distributed, mainly due to variation in menstrual blood losses among women. To account for this, we used published requirement distributions for each age–sex–physiology group (e.g. men, women, adolescents, pregnancy, lactation, children) and assigned probabilities of adequacy based on how each individual's usual intake compared to these distributions (Institute of Medicine 2001). To provide a summary measure of overall dietary adequacy, we calculated the Mean Probability of Adequacy (MPA), defined as the unweighted mean of the individual PAs across the 11 nutrients of interest.

Supplementary Method S7. Plant-pollinator visitation survey methods

Plant and pollinator surveys were conducted every two weeks from 18 April to 4 November 2021. The flowering, cropping and active pollinator season in Jumla usually begins at the start of April and ends in November with the arrival of winter. In each of the 10 study villages, a 600 x 600 metre (36 hectare) sampling area was drawn around the centre point of the village and the landscape inside this study area was divided into three different landcover categories and delineated in QGIS (Version 3.16.8-Hanover). The three landcover types were: village (village houses and associated vegetable gardens); crop (areas under arable cultivation); and semi-natural (non-cultivated areas including pasture, forest, scrub etc.) (Figure S2). For each of the three habitat types, three fixed survey plots of 60 m x 60 m were randomly located in each village using QGIS; these were then ground-truthed to check that they were safe and accessible for data collectors to visit. This gave nine plots in total for each village and 90 plots from all 10 villages. Every two weeks from April-November, a 40-minute survey was conducted to record the visitation of insect pollinators to crop and non-crop plants inside each plot. To our knowledge there are no vertebrate crop pollinators in Nepal, so we limited our surveys to insects only. Surveys were conducted between the hours of 09.00 and 17.00 when temperatures exceeded 15°C and when rain was absent or light. During each survey, data collectors spent 40 minutes walking haphazardly at a constant pace between flowering patches within the plot, and captured any insect they observed visiting a flower. The details of each capture event (including host plant species, specimen code, time, and location) were recorded on Android tablets using a custom-built data collection app in CommCare Version 2.48.3 (http://www.commcarehq.org/home/), an open-source, cloud-based data collection platform. Sampling permission was provided by the Nepal Ministry of Forest and Environment [Ref: 258].

All captured insect specimens were transferred to a killing tube containing ethyl acetate and subsequently pinned (or transferred to paper envelopes in the case of moths and butterflies) and labelled to await identification. Plants were identified by data collectors using a custom-made Plant Atlas for Jumla District, and species identifications were checked for accuracy by a botanist from the national herbarium of Nepal. All insect specimens were identified by insect taxonomists (see acknowledgements) and are stored in the Central Department of Zoology, Tribhuvan University, Nepal. Insect identifications were checked for accuracy by an independent taxonomist from the University of Agricultural Sciences, India.

Supplementary Method S8. Pollen quantification: methods and caveats

In scoring pollination services over space and time, we were faced with three key challenges which prevented us from measuring pollination services directly, for example through the use of single-visit pollen deposition (King *et al.* 2013). First, the system is characterised by a high diversity of crops and pollinators, thus necessitating the measurement of pollination of a wide range of insect taxa visiting a wide range of plant species. Second, our metrics of pollinator importance across the full season called for high levels of replication throughout the year. Third, our analyses of consistency in patterns across villages called for high levels of replication in space. These challenges prevented us from directly measuring pollen deposition by insects. Nonetheless, we went substantially further than simply measuring pollinator visitation, as we weight the visitation frequency of each insect taxon by its pollen carrying capacity. Although not all of the pollen grains on an insect's body are deposited, we assume that insects carrying more pollen grains have greater potential for depositing grains during a visit than insects carrying few pollen grains (consistent with (Howlett *et al.* 2011; Földesi *et al.* 2021).

A total of 1928 insect specimens were sampled for pollen, representing 18% of the total insect specimen collection. Insects were selected by stratified random sampling to limit our sample to only those taxa which were recorded visiting crops plants (rather than those exclusively visiting wild plants) and only those captured during the main period of crop flowering (April-September). This process ensured we had multiple replicates of the insect taxa most-frequently recorded visiting crops.

The number of pollen grains on each individual insect specimen was quantified by swabbing the insect with glycerine jelly and counting the total number of pollen grains on each insect using light microscopy. The glycerine jelly solution was prepared by adding 10 grams of gelatine crystals to 25 ml water and 30 ml glycerol and adding a few drops of Sainfrin red stain until the solution was strongly pink but still transparent. The solution was heated and mixed to dissolve all crystals and then poured into a petri dish to a depth of c.5 mm and left to cool and solidify in a fridge for 20 minutes. The thin layer of jelly was then cut into an even grid of 3 mm^3 cubes.

Insects were only sampled if they were in suitably good condition (i.e. showed no major damage like a lost head, abdomen or thorax); in case of major damage, a reserve specimen was selected instead. A single cube of jelly was held with a pair of forceps and used to swab each insect across the entire right-hand side of its body. We consistently limited pollen sampling to the right-hand side of the insect's body to reduce the number of pollen grains that need to be counted; this assumes the insects are bilaterally symmetrical with regard to pollen deposition. Final counts were multiplied by two in all cases to scale up to the level of the whole insect. Areas sampled include the right-hand side of the insect's face, head, back, side, front legs, and underside. The only areas avoided were those where pollen had been gathered for transport to the nest and was therefore unavailable for pollination, such

as pollen collected in the pollen baskets (corbiculae) on the back legs of a honey bee or the pollen bundles underneath a solitary bees' legs.

Once all pollen was collected from the body, we placed the cube of jelly on a clean glass slide and put a cover slip on top. The jelly was gently heated over a low flame until it started to melt and spread evenly across the slide. Pollen grains were then counted systematically under a light microscope (Zeiss Primo star), using a haemocytometer. In cases where it was impossible to count all grains, we sub-sampled the slide (using the haemocytometer grid), and counted only the grains in the right-hand half or bottom-right quarter; values were then multiplied up accordingly. Finally, we calculated the mean pollen carrying capacity of each insect taxon by taking a mean of the total number of pollen grains recorded across all replicates of the taxon. The pollen grains on each insect were not identified to species (impractical in a study of this scale), thus we do not differentiate between conspecific and heterospecific pollen loads and our measures of pollen carrying capacity are limited to one value per insect taxon, rather than specific values for each plant-pollinator combination.

Supplementary Method S9. Pollinator exclusion experiments

During the field season of 2022, we conducted a series of pollinator exclusion experiments to record the pollinator dependence of four important crops in our study area. The reasons for these experiments were two-fold: 1) to check the consistency of pollinator-dependence values in our local area with values reported in the literature; and 2) to obtain pollinator dependence values for important local crops that could not be found in the literature (e.g., the slipper gourd, *Cyclanthera pedata,* a member of the Cucurbitaceae family). The crops that were surveyed included: pumpkin (*Cucurbita maxima*), Jumli bean (*Phaseolus vulgaris*), slipper gourd (*Cyclanthera pedata*), and apple (*Malus domestica*; Red Delicious variety which is the most common cultivar). These crops were chosen as they are the most frequently grown and economically or nutritionally important pollinator dependent crops in our study region. We conducted the experiments in 12 separate villages in Patarasi Rural Municipality, Jumla. These included the 10 original study villages (Fig. S2), plus two extra villages (Huri and Mahuri) in the same municipality. To increase the number of high-altitude plots and to spread the sites more evenly across an altitudinal gradient, we selected both a high and a low experimental site in the three highest villages (Huri, Mahuri and Patmara). These sampling sites were assigned to the highest and lowest point of the village, at least 300 m distance apart. This gave a total of 15 experimental sites along an altitudinal gradient spanning 2400 – 3100 metres above sea level. For the three annual crops (pumpkin, Jumli bean and slipper gourd), we selected a small plot of agricultural land to cultivate replicate plants under standardised conditions (same watering/ fertiliser/ weeding regime). For apple, we selected 10 well-established Red Delicious apple trees in each site to serve as our experimental plants.

To each of these crop species at each site, we applied three experimental treatments: 1) Pollinator exclusion - flowers covered with a netting bag to exclude all insects; 2) Open pollination - flowers freely visited by the insect pollinator community; 3) Hand pollination - flowers pollinated with a paintbrush to achieve supplemental pollination. The number of replicate plants for each treatment at each site was: 10 apple trees, 12 pumpkin vines, 8 bean plants and 12 slipper gourd vines. Experimental treatments were applied at different scales for each crop, depending on what was most practical and biologically sensible. Thus, treatments were applied to individual plants for beans, individual branches on the same tree for apples, and individual flowering nodes for pumpkins and slipper gourds. Treatments were applied during the flowering period only and then removed so that all experimental branches/ plants/ fruits could develop under exactly the same conditions. At the time of fruit maturation (between September and October, depending on the crop), each experimental plant was harvested, and the yield recorded using Salter weighing scales.

Unfortunately, due to Covid-19 lockdowns coinciding with the training and set-up period, the hand pollination treatments were not implemented correctly. In many sites, flowers were damaged during

manipulation, leading to aborted flowers and no fruit development. As a result, these data were not usable. We therefore restricted our analyses to the pollinator exclusion and open pollination treatments. From these, we determined the pollinator dependence of each crop and each site by calculating the percentage reduction in yield when pollinators were excluded from the crop compared to when pollinators were free to visit. We then calculated a mean pollinator dependence value for each crop across all 15 sites. Overall, our results aligned closely with published values in the literature (Klein *et al.* 2007; Silva *et al.* 2021).

Supplementary Method S10. Farmer questionnaire surveys for economic assessments

During September 2022, data collectors conducted structured interviews with the lead farmer of each of the 200 study households (the person responsible for making most of the agricultural decisions in the household). Respondents were asked a range of questions about their farming practices and their agroecological and environmental awareness, as well as their different sources of farming income. Each respondent was asked to report the total economic revenue (in Nepalese rupees) generated from the sale of agricultural products during the previous 12-month period. They were then asked to list all of the pollinator-dependent crops grown on their farm in the previous 12-months and report the total economic revenue generated from each of these crops. Thus, for each pollinator-dependent crop, we were able to calculate its contribution to total farming income and estimate the economic losses that would result from changes in the yield of each crop (as a result of changes in the pollination service). To minimise errors, interviewers read the response to each economic question (i.e. the value) back to respondents and asked them to confirm it before moving on.

Supplementary Method S11. Estimating local pollinator declines: rationale and caveats

Despite the immense importance of pollination services to smallholder farmers, we know almost nothing about the population trends of insect pollinators in regions of the world where smallholder farming systems predominate, including in Nepal. This lack of historical data makes it challenging to assess changes in pollinator abundance over time and predict the corresponding impacts on yields of pollinator-dependent crops. In the absence of long-term quantitative data on insect population trends, we must rely on other forms of data to assess pollinator population trends in under-studied regions of the world. One option is to utilise the records and perceptions of local beekeepers who typically have a strong awareness of the health and population trends of their bees and the environment in which they forage.

Following this approach, we draw on the findings of Kortsch *et al.* (2024), who documented steep and ongoing declines in honey production per hive of the native honeybee *Apis cerana* in our study region, and use their results as a proxy for broader pollinator change. Kortsch *et al.* (2024) report a 44% decline in the number of occupied hives and a 50% decline in honey yields per hive from 2012-2021, and extrapolate these trends to predict a further 33% decline in honey yields (low-CI: 22%; upper-CI: 42%) between 2021 and 2030. We use this figure of 33% as a conservative best-guess estimate of wider pollinator community declines by 2030. This trajectory is not intended as a definitive forecast, but rather as a benchmark scenario, which we use to extract results from our simulations of continuous pollinator decline in order to estimate likely impacts on crop yields and nutrient intake.

We acknowledge that extrapolating honeybee yield trends to wild pollinator communities involves important assumptions. Social species such as *Apis cerana* may not respond to stressors in the same way as solitary pollinators, and honey yield is only an indirect indicator of population abundance. However, we justify the use of this benchmark for the following reasons:

1) As far as we are aware, it is the only dataset available from Nepal that provides a quantitative indication of pollinator population trends. Moreover, the rate of decline is highly consistent with modelled estimates of wild insect declines elsewhere (Millard *et al.* 2023).

2) Honeybees are generalist foragers whose accumulation of honey is largely determined by the quantity and quality of floral resources present in the landscape, as well as local climatic conditions which influence their ability to forage (Quinlan *et al.* 2023). Honey yields can therefore serve as an informative bioindicator of floral resource availability and foraging conditions for the wider pollinator community (Quinlan *et al.* 2023).

3) Beekeepers in this study reported changing weather patterns and reduced flower availability as the two most important drivers of honey yield decline (Kortsch *et al.* 2024); these same factors are also

well established as major constraints on non-honeybee pollinator populations (Roulston & Goodell 2011; Müller *et al.* 2023).

4) Evidence from other regions suggests that wild pollinators often experience even steeper declines than managed honeybees (Zattara & Aizen 2021; Aldercotte *et al.* 2022). We therefore interpret the *Apis cerana* trend as a conservative proxy for wider pollinator declines, while clearly recognising the uncertainty and limitations of this approach.

Supplementary Method S12. Predicting yield increases from optimum pollination

Our aim in this analysis was to calculate yield gaps for a range of pollinator-dependent crops grown in our study region and use these values to determine the likely increase in yields that would be attainable by enhancing pollination services. In a large-scale international study looking at the benefit of pollinator abundance and diversity for crop yields, (Garibaldi *et al.* 2016) showed that yield gaps for a range of pollinator-dependent crops could be closed by a median of 24% through increasing flower visitor density. They calculated this figure by analysing how variation in flower visitor density across sites explained yield differences, after accounting for other agronomic inputs (e.g. fertiliser, water, management). Their study collected data from 344 fields from 33 pollinator dependent crop systems in Africa, Latin America and Asia (including in Nepal) from both small and large farms between 2010 and 2014. Regions were chosen to focus primarily on low income countries. Given the similarity between the scale and context of farming in (Garibaldi *et al.* 2016) and our study region, we made the assumption that approximately the same proportion of the yield gaps in our study region could be closed by optimising pollination services. Thus, we only reduced the portion of the yield gap attributed to insufficient pollination; we do not assume any other changes in management (e.g. fertilizer or water input) which might further close the yield gap. Following (Smith *et al.* 2022), we fit a Gaussian distribution to the pollinator-attributable yield gap percentages reported by (Garibaldi *et al.* 2016) and identified the mean along with its 95% confidence interval (CI) (25.5%; 95% CI: 5.5%, 45.4%) for use in our model.

We measured local yield gaps in the four locally-grown pollinator-dependent crops that are most abundantly consumed in Jumla District (based on total grams of consumption); these were: Jumli beans (*Phaseolus vulgaris*), mustard (*Sinapis alba*), apple (*Malus domestica*), and pumpkin (*Cucurbita maxima*). Collectively, these four crops comprised more than 75% of the total consumption of local pollinator dependent crops (Jumli beans: 47%; mustard: 13%; apples: 11%, and pumpkin: 5%). From an economic perspective, apples, Jumli beans, and mustard are also the three most economically-important crops in Jumla, collectively making up 93% of total farming income.

For each of these four crops, we measured yields in 15-33 separate locations around Jumla District and yield gaps were calculated as the difference between the 50th and 90th percentile recordings, following (Smith *et al.* 2022). We recorded yields in slightly different ways for each crop, to align with the way in which the crop is typically grown and harvested. For beans and mustard which are grown in relatively uniform patches and harvested in a single event, we randomly located three 1x1 m quadrats in 33 different study fields and recorded the total dry weight of all seeds harvested from within the quadrat. A mean of the three quadrats was taken for each field and this value served as the crop yield for that site, expressed in grams per square metre. For apples, which are grown in orchards, we randomly selected five healthy Red Delicious apple trees from 33 different apple orchards to serve as our study trees. During harvest time, the total number of apples on each tree was counted and a

random sample of five apples was taken from each tree and weighed. The mean apple weight per tree was multiplied by the total number of apples on the tree to give an estimate of the total weight of apples produced by the tree. For each site, we took a mean value of total apple weight per tree across the five study trees to represent our yield value for that site. For pumpkins which are grown haphazardly as vines, with no fixed unit of area, we cultivated 12 replicate plants of a uniform size in 15 different sites across Patarasi Municipality. On each plant, we marked five female flower buds and monitored their development. At the time of harvest, we recorded how many of these flowers had developed into fruit and then recorded the weight of each of the successful fruit. Whilst this does not provide a yield value per unit area, it gave us a unit of measurement that was entirely comparable amongst sites, enabling us to assess the variation in yield and calculate percentage yield gaps.

Due to time and resource constraints, we were unable to record yields and calculate yield gaps for all pollinator-dependent crops grown in the region. For crops within the same taxonomic family as one of the four crops recorded (beans: Fabaceae; mustard: Brassicaceae; apple: Rosaceae; and pumpkin: Cucurbitaceae), we applied the yield gap value from these recorded crops. For crops in different families to those recorded, we simply applied the lowest recorded yield gap value to provide a conservative estimate of the potential for yield increases. Although these assumptions are simplistic, the crops for which this approach were applied constituted less than 25% of the total consumption of pollinator-dependent crops and therefore changes in their yield gap estimates have limited influence on overall nutrient intake (confirmed through a sensitivity analysis). By assuming a yield gap value of zero for all unrecorded crops, we would be sure to underestimate the potential for increased yields, thus we concluded that imputing yield gap values from recorded crops was likely to provide us with the most accurate assessment of the potential for yield increases.

The pollinator-associated yield enhancements predicted by our analysis align closely with those reported in empirical studies where the influence of pollinator abundance and diversity on crop yields are directly measured e.g. (Bartomeus *et al.* 2014; Blaauw & Isaacs 2014).

Supporting Tables

Table S1. Crops recorded in Jumla study sites.

List of all 50 crop species recorded in the plant-pollinator surveys in the 10 study villages, including their family name, English name and Nepali name.

Plant family	Crop scientific name	Crop English name	Crop Nepali name
Alliaceae	*Allium* sp.	Weave garlic	NA
Amaranthaceae	*Beta vulgaris*	Swiss chard	Swiss saag
Amaranthaceae	*Spinacia oleracea*	Spinach	Palungo saag
Amaryllidaceae	*Allium cepa*	Onion	Pyaz / Pyaaj
Amaryllidaceae	*Allium sativum*	Garlic	Lasun
Amaryllidaceae	*Allium tuberosum*	Chinese leek	Dundu saag
Apiaceae	*Anethum graveolens*	Dill	Saunph / Sop
Apiaceae	*Coriandrum sativum*	Coriander	Dhaniya
Apiaceae	*Daucus carota*	Carrot	Gajar
Apiaceae	*Foeniculum vulgare*	Fennel	Saunph saag
Araceae	*Colocasia esculenta*	Taro	Karkalo / Gaava / Pidhaalu
Asteraceae	*Helianthus annuus*	Sunflower	Suryamukhi
Basellaceae	*Basella alba*	Creeping spinach	Bhadae saag
Brassicaceae	*Brassica alba*	Broad leaf mustard	Rayo bhaji saag
Brassicaceae	*Brassica oleracea var. capitata*	Cabbage	Banda kovi
Brassicaceae	*Brassica rapa*	Turnip	Gante mula
Brassicaceae	*Lepidium sativum*	Garden cress	NA
Brassicaceae	*Raphanus sativus*	Radish	Choto / Koiro
Brassicaceae	*Raphanus sativus var. longipinnatus*	Daikon	Mula
Cannabaceae	*Cannabis sativa*	Cannabis	Bhango
Cucurbitaceae	*Cucumis sativus*	Cucumber	Kakhara
Cucurbitaceae	*Cucurbita maxima*	Pumpkin	Kaddu
Cucurbitaceae	*Cyclanthera pedata*	Slipper gourd	Chuche karela / Barela
Cucurbitaceae	*Cucurbita pepo*	Courgette	NA
Cucurbitaceae	*Momordica balsamina*	Balsam apple	Ban karela
Fabaceae	*Canavalia ensiformis*	Jack bean	Goreto simi
Fabaceae	*Phaseolus coccineus*	Scarlet bean	Ghogate simi
Fabaceae	*Phaseolus vulgaris*	Jumli bean	Jumli simi
Fabaceae	*Pisum sativum*	Garden pea	Kerau mattar
Fabaceae	*Pisum sativum var. arvense*	Field pea	Saano kalaa / Naano kalaa
Juglandaceae	*Juglans regia*	Walnut	Daatee okhar
Lamiaceae	*Mentha × piperita*	Peppermint	Putina
Lamiaceae	*Origanum vulgare*	Oregano	Ram tulasi
Lamiaceae	*Perilla frutescens*	Perilla	Tilkhudo / Tilkuro
Poaceae	*Eleusine coracana*	Finger millet	Kodo
Poaceae	*Hordeum vulgare*	Barley	Jahu
Poaceae	*Hordeum vulgare subsp. vulgare*	Naked barley	Mudule Jahu / Uwa

Poaceae	*Sorghum bicolor*	Sorghum	Junelo
Poaceae	*Zea mays*	Maize	Makkai
Polygonaceae	*Fagopyrum esculentum*	Sweet buckwheat	Mite phapar
Polygonaceae	*Fagopyrum tataricum*	Tartary buckwheat	Tite phapar
Rosaceae	*Malus domestica*	Apple	Shyau
Rosaceae	*Prunus domestica*	Plum	Khurpani / Aru bokhara
Rosaceae	*Prunus persica*	Peach	Aaru
Solanaceae	*Capsicum sp.*	Chilli	Khursani
Solanaceae	*Cyphomandra betacea*	Tree tomato	Tyammatar
Solanaceae	*Lycopersicon esculentum*	Tomato	Golveda / Golbera
Solanaceae	*Nicotiana tabacum*	Tobacco	Surti
Solanaceae	*Solanum melongena*	Aubergine	Bhyanta
Solanaceae	*Solanum tuberosum*	Potato	Aalu

Table S2. List of key pollinator-dependent crops cultivated in Jumla

Crops are shown alongside their degree of pollinator dependence and the source of this information (values are a mean of all listed sources). Also shown is the percentage of farmers recorded growing the crop in our study villages

Crop category	Crop scientific name	Crop family	English name	Nepali name	Pollinator dependence	Pollinator dependence source	% farmers growing crop
Cereal	*Fagopyrum tataricum*	Polygonaceae	Buckwheat	Phapar	0.65	1	27.0%
Fruit/nut	*Malus domestica*	Rosaceae	Apple	Shyau	0.80	1,2	77.0%
Fruit/nut	*Prunus armeniaca*	Rosaceae	Apricot	Chule aru	0.65	1	37.0%
Fruit/nut	*Prunus domestica*	Rosaceae	Plum	Khurpani	0.65	1	34.0%
Fruit/nut	*Prunus persica*	Rosaceae	Peach	Aaru	0.65	1	16.0%
Fruit/nut	*Pyrus communis*	Rosaceae	Pear	Naspati	0.65	1	12.5%
Herb/spice	*Capsicum sp.*	Solanaceae	Chilli	Khursani	0.43	1,3,4	89.0%
Oilseed	*Brassica alba*	Brassicaceae	Mustard seed	Tori	0.25	1	24.5%
Oilseed	*Helianthus annuus*	Asteraceae	Sunflower	Suryamukhi	0.25	1	10.5%
Pulse	*Glycine max*	Fabaceae	Soybean	Bhatmas	0.30	5	61.0%
Pulse	*Phaseolus coccineus*	Fabaceae	Scarlet bean	Ghogate simi	0.25	1	6.0%
Pulse	*Phaseolus vulgaris*	Fabaceae	Green bean	Jumli simi	0.25	1	10.5%
Pulse	*Phaseolus vulgaris*	Fabaceae	Jumli bean	Ghyu simi	0.20	1,2	99.0%
Pulse	*Vigna unguiculata*	Fabaceae	Cowpea	Bodi	0.25	1	1.5%
Vegetable	*Cucumis sativus*	Cucurbitaceae	Cucumber	Kakhara	0.65	1	51.5%
Vegetable	*Cucurbita maxima*	Cucurbitaceae	Pumpkin	Kaddu	0.95	1,2	79.5%
Vegetable	*Cyclanthera pedata*	Cucurbitaceae	Slipper gourd	Chuche karela	0.88	2	51.0%
Vegetable	*Cyphomandra betacea*	Solanaceae	Tree tomato	Rukh tamatar	0.65	6	4.5%
Vegetable	*Lycopersicon esculentum*	Solanaceae	Tomato	Golveda	0.05	1	36.5%
Vegetable	*Momordica charantia*	Cucurbitaceae	Bittergourd	Tite karela	0.25	1	1.0%
Vegetable	*Solanum melongena*	Solanaceae	Aubergine	Bhanta	0.25	1	20.5%

Sources: 1 = Silva *et al.* (2021); 2 = Author's measurements (Supplementary Method S9); 3 = Wanza Soli *et al.* (2020); 4 = Yourstone *et al.* (2021); 5 = da Cunha *et al.* (2023); 6 = Ahmad Shah *et al.* (2023)

Table S3. Pollinator Contribution (PC) scores of each insect for each key micronutrient

Values show the 30 most important insect pollinator taxa in our study system, ranked by their mean contribution to six key pollinator-dependent micronutrients. Values show the Pollinator Contribution (PC) scores of each insect (see equation in the main text) which represents their estimated contribution to the total intake of each nutrient across the study population. For example, the PC score of *Apis cerana* for folate implies that 5.12% of folate intake is attributable to *Apis cerana* via its pollination (and resulting yield enhancement) of the crops that provide people's folate in this region.. For village-specific values, see Fig. S5b.

**Insect taxon**	**Calcium**	**Folate**	**Iron**	**Vit. A**	**Vit. C**	**Vit. E**	**Mean**
Apis cerana	4.86%	5.12%	3.17%	6.61%	3.92%	1.96%	4.27%
Bombus tunicatus	1.37%	2.86%	1.34%	1.23%	0.35%	1.96%	1.52%
Bombus spp.	1.35%	2.88%	1.49%	1.10%	0.28%	0.44%	1.26%
Apis laboriosa	1.20%	3.05%	1.36%	0.03%	0.01%	0.26%	0.98%
Eristalis tenax	0.65%	0.50%	0.34%	1.77%	0.76%	0.23%	0.71%
Tetralonia sp.02	0.46%	1.14%	0.53%	0.09%	0.01%	0.09%	0.39%
Bombus asiaticus	0.42%	1.02%	0.46%	0.05%	0.02%	0.25%	0.37%
Lasioglossum sp.02	0.31%	0.43%	0.25%	0.28%	0.34%	0.15%	0.29%
Andrena sp.01	0.32%	0.65%	0.33%	0.17%	0.04%	0.09%	0.27%
Andrena sp.02	0.16%	0.07%	0.14%	0.61%	0.13%	0.15%	0.21%
Eristalis arbustorum	0.28%	0.06%	0.15%	0.48%	0.14%	0.09%	0.20%
Anthophora sp.02	0.22%	0.49%	0.22%	0.11%	0.04%	0.07%	0.19%
Hymenoptera	0.15%	0.27%	0.11%	0.24%	0.30%	0.08%	0.19%
Diptera spp.	0.18%	0.29%	0.14%	0.07%	0.12%	0.11%	0.15%
Popillia sp.02	0.13%	0.32%	0.14%	0.01%	0.00%	0.02%	0.10%
Halictus sp.01	0.04%	0.02%	0.02%	0.26%	0.05%	0.02%	0.07%
Anthophora sp.03	0.04%	0.09%	0.04%	0.16%	0.04%	0.03%	0.07%
Mylabris sp.01	0.06%	0.14%	0.07%	0.04%	0.02%	0.02%	0.06%
Halictus sp.02	0.05%	0.02%	0.02%	0.13%	0.07%	0.01%	0.05%
Andrena sp.04	0.05%	0.02%	0.02%	0.13%	0.06%	0.02%	0.05%
Eupeodes sp.03	0.05%	0.04%	0.03%	0.07%	0.08%	0.02%	0.05%
Eristalis sp.01	0.06%	0.11%	0.06%	0.02%	0.01%	0.03%	0.05%
Lasioglossum sp.01	0.05%	0.02%	0.03%	0.12%	0.04%	0.02%	0.05%
Tenthredo sp.01	0.08%	0.01%	0.03%	0.11%	0.04%	0.01%	0.05%
Megachile spp.	0.06%	0.15%	0.06%	0.00%	0.00%	0.01%	0.05%
Halictus sp.03	0.03%	0.02%	0.02%	0.13%	0.06%	0.01%	0.04%
Mylabris sp.02	0.05%	0.13%	0.06%	0.00%	0.00%	0.01%	0.04%
Syrphidae spp.	0.04%	0.01%	0.02%	0.12%	0.04%	0.01%	0.04%
Augochlorella sp.01	0.06%	0.03%	0.02%	0.05%	0.07%	0.00%	0.04%
Bombus trifasciatus	0.02%	0.01%	0.01%	0.08%	0.02%	0.08%	0.04%
Total pollinator dependence	**14.3%**	**19.2%**	**10%**	**21.3%**	**14.2%**	**3.6%**	

Table S4. Food item contributions to key micronutrients

Values show the 40 most important crops/non-crop food items based on their contribution to six key micronutrients. Values show the percentage contribution to the total supply of each nutrient across the study population, as well as the mean percentage contribution across all six nutrients to give a sense of overall nutritional value. For village-specific values, see Fig. S5a.

Crop / food item	Scientific name	Calcium	Folate	Iron	Vit. A	Vit. C	Vit. E	Mean
Jumli bean	*Phaseolus vulgaris*	21.9%	55.9%	24.8%	0.3%	0.0%	3.5%	17.7%
Potato	*Solanum tuberosum*	6.8%	10.3%	9.8%	0.0%	65.9%	3.4%	16.1%
Broad leaf mustard	*Brassica alba*	7.8%	4.1%	2.9%	34.8%	11.4%	2.1%	10.5%
Soybean	*Glycine max*	0.6%	2.2%	0.7%	0.1%	0.1%	43.3%	7.8%
Rice	*Oryza sativa*	9.1%	8.7%	15.9%	0.0%	0.0%	7.8%	6.9%
Finger millet	*Eleusine coracana*	8.4%	3.8%	10.0%	0.0%	0.0%	1.0%	3.9%
Fortified food	NA	2.1%	0.6%	0.9%	12.7%	2.5%	3.8%	3.7%
Wheat	*Triticum sp.*	3.1%	3.8%	8.2%	0.1%	0.0%	5.0%	3.4%
Dairy	NA	5.6%	0.3%	0.2%	4.0%	0.4%	1.4%	2.0%
Sunflower	*Helianthus annuus*	0.0%	0.0%	0.0%	0.0%	0.0%	10.3%	1.7%
Stinging nettle	*Urtica dioica*	10.2%	0.0%	0.0%	0.0%	0.0%	0.0%	1.7%
Market item	NA	1.9%	2.1%	2.5%	0.4%	0.0%	3.3%	1.7%
Nepalese Allium	*Allium hypsistum*	0.7%	0.1%	0.4%	5.4%	2.4%	0.0%	1.5%
Taro	*Colocasia esculenta*	0.8%	0.5%	0.3%	6.5%	0.6%	0.0%	1.5%
Chilli	*Capsicum sp.*	1.0%	0.3%	1.3%	4.4%	0.5%	1.2%	1.5%
Wild plant	NA	0.7%	0.4%	0.4%	5.3%	1.0%	0.3%	1.4%
Maize	*Zea mays*	1.9%	0.3%	4.8%	0.7%	0.0%	0.3%	1.3%
Pumpkin	*Cucurbita maxima*	0.6%	0.6%	0.2%	2.5%	0.8%	1.4%	1.0%
Mixed vegetable oil	NA	0.0%	0.0%	0.0%	0.0%	0.0%	5.9%	1.0%
Alcohol	NA	3.1%	0.0%	2.2%	0.0%	0.1%	0.0%	0.9%
Cabbage	*Brassica oleracea*	0.9%	0.8%	0.1%	0.1%	2.6%	0.1%	0.8%
Tomato	*Lycopersicon esculentum*	0.1%	0.1%	0.0%	3.5%	0.8%	0.1%	0.8%
Turmeric	*Curcuma longa*	0.3%	0.0%	2.8%	0.0%	0.0%	0.6%	0.6%
Cauliflower	*Brassica oleracea*	0.4%	0.5%	0.3%	0.0%	2.5%	0.0%	0.6%
Coriander	*Coriandrum sativum*	2.1%	0.1%	1.2%	0.1%	0.0%	0.2%	0.6%
Radish	*Raphanus sativus*	1.0%	0.7%	0.3%	0.1%	1.4%	0.0%	0.6%
Cumin seed	*Cuminum cyminum*	1.9%	0.1%	1.2%	0.1%	0.0%	0.3%	0.6%
Arum	*Arum spp.*	0.3%	0.2%	0.1%	2.4%	0.2%	0.0%	0.6%
Chinese mallow	*Malva verticillata*	0.3%	0.1%	0.2%	2.0%	0.4%	0.1%	0.5%
Barmola	*Megacarpaea polyandra*	0.3%	0.1%	0.2%	1.9%	0.4%	0.1%	0.5%
Cobra lily	*Arisaema utile*	0.3%	0.1%	0.2%	1.7%	0.3%	0.1%	0.5%
Barley	*Hordeum vulgare*	0.5%	0.1%	1.1%	0.0%	0.0%	0.8%	0.4%
Coriander leaf	*Coriandrum sativum*	0.2%	0.1%	0.2%	1.8%	0.2%	0.1%	0.4%
Egg	NA	0.2%	0.1%	0.2%	1.5%	0.0%	0.4%	0.4%
Buckwheat leaves	*Fagopyrum esculentum*	0.3%	0.1%	0.2%	1.3%	0.2%	0.1%	0.4%
Mixed spice	NA	0.3%	0.0%	0.5%	1.3%	0.0%	0.0%	0.4%
Apple	*Malus domestica*	0.3%	0.2%	0.3%	0.0%	1.1%	0.2%	0.4%
Garlic	*Allium sativum*	0.2%	0.7%	0.2%	0.2%	0.6%	0.0%	0.3%
Slipper gourd	*Cyclanthera pedata*	1.0%	0.0%	0.3%	0.0%	0.2%	0.0%	0.3%

Table S5. Nutritional status of study population

Values show the cluster-adjusted mean, standard error and upper and lower 95% confidence intervals of the percentage of individuals within each population subgroup who are classified as being of a certain nutritional status. For adult men and women, their mean weight, height and BMI scores are also given. Abbreviations used in table: HAZ = Height-for-age z score; LAZ = Length-for-age z score; WAZ = Weight-for-age z score; WHZ = Weight-for-Height for age z score; WLZ = Weight-for-Length z score; BMIZ = BMI-for-age z score; BMI = body mass index.

**Children 6 to 59 months**	**Mean value**	**Std. Err**	**95% CI lower**	**95% CI upper**	**Variable definition**
Stunted	50.5%	3.3%	44.1%	56.9%	Stunted (LAZ or HAZ<-2 too short for age)
Underweight	24.3%	2.7%	18.9%	29.7%	Underweight (WAZ<-2 weighs too little for age)
Wasted	4.9%	1.1%	2.7%	7.1%	Wasted (WLZ or WHZ<-2 too thin for height)
Low BMI for Age Z score	4.3%	1.0%	2.4%	6.2%	Underweight by BMIZ <-2 (too thin for height)
Severely Stunted	17.2%	2.4%	12.5%	22.0%	Severe stunting <-3 LAZ or HAZ scores
Severely Underweight	6.0%	1.4%	3.2%	8.8%	Severe underweight <-3 WAZ scores
Severely Wasted	0.7%	0.3%	0.1%	1.4%	Severely wasted <-3 WLZ or WHZ scores
Severely Low BMI for Age Z score	0.4%	0.2%	0.1%	0.7%	Severely low BMI <-3 BMIZ scores
Overweight/obese	10.8%	1.6%	7.8%	13.9%	Overweight/obese >1 BMI Z for age
Obese	1.6%	0.4%	0.7%	2.5%	Obese >2 BMI Z for age
**Adolescent girls**					
Stunted	34.7%	3.2%	28.5%	41.0%	Stunted (LAZ or HAZ<-2 too short for age)
Low BMI -for-Age Z score	8.1%	1.7%	4.8%	11.4%	Underweight by BMIZ <-2 (too thin for height)
Severely Stunted	6.9%	1.7%	3.6%	10.2%	<-3 LAZ or HAZ scores
Severely Low BMI-for-Age Z score	0.7%	0.3%	0.2%	1.3%	Severely low BMI <-3 BMIZ scores
Overweight/obese	1.4%	0.5%	0.5%	2.4%	Overweight/obese >1 BMIZ for age
Obese	0.0%	0.0%	0.0%	0.0%	Obese >2 BMI Z for age
**Adult women**					
Short stature <145cm	9.7%	2.0%	5.7%	13.7%	
Underweight BMI<18.5 g/m^2	13.6%	1.9%	9.8%	17.4%	
Overweight BMI>=25 g/m^2	5.5%	1.3%	2.9%	8.1%	
Obese BMI>=30 g/m^2	0.1%	0.1%	-0.1%	0.3%	
Weight (kg)	47.7	0.4	46.9	48.5	
Height (cm)	151.4	0.3	150.7	152.1	
BMI (kg/m^2)	20.8	0.2	20.5	21.1	
**Adult men**					
Short stature <145cm	1.6%	0.3%	1.0%	2.3%	
Underweight BMI<18.5 g/m^2	11.2%	1.9%	7.5%	14.8%	
Overweight BMI>=25 g/m^2	8.4%	1.9%	4.7%	12.2%	
Obese BMI>=30 g/m^2	1.1%	0.7%	-0.2%	2.3%	
Weight (kg)	54.3	0.5	53.4	55.3	
Height (cm)	159.9	0.5	158.9	160.9	
BMI (kg/m^2)	21.3	0.2	20.9	21.7	

Table S6. Nutrient intake, requirements and probability of adequacy scores

Values show the daily intake of each macronutrient and 12 key micronutrients as well as the estimated average requirements (EAR) and probability of adequacy for each of the micronutrients. Values are expressed as the mean and standard deviation (SD) for each population subgroup. The sample size (n) of each group is as follows: adolescent girls: 190; adult women: 215; adult males: 186; under-5 children: 185.

Nutrient	Metric	Adolescent girl		Adult woman		Adult male		Under-five child	
		Mean	SD	*Mean*	SD	*Mean*	SD	*Mean*	SD
**Energy**	Daily intake (Kcal)	2245.4	590.1	2773.3	726.6	3023.7	727.1	998.7	374.7
**Fat**	Daily intake (g)	34.3	13.9	42.5	26.3	84.4	108.2	18.8	7.5
**Protein**	Daily intake (g)	57.2	16.6	70.9	22.2	77.1	21.9	26.2	10.4
**Vitamin A**	Daily intake (µg)	123.5	91.4	168.4	156.9	171.0	105.4	104.0	78.2
	Daily req. (µg)	444.2	83.7	465.3	73.8	428.6	85.7	298.2	59.6
	**Prob. adequacy**	**1.6%**	**8.3%**	**5.2%**	**15.3%**	**4.2%**	**14.0%**	**5.0%**	**17.5%**
**Vitamin B1 (Thiamin)**	Daily intake (mg)	0.86	0.26	1.09	0.37	1.14	0.40	0.41	0.16
	Daily req. (mg)	0.96	0.10	1.06	0.11	1.00	0.10	0.40	0.05
	**Prob. adequacy**	**32.1%**	**37.7%**	**47.1%**	**43.2%**	**60.6%**	**42.4%**	**46.4%**	**40.3%**
**Vitamin B2 (Riboflavin)**	Daily intake (mg)	0.53	0.16	0.65	0.25	0.69	0.21	0.34	0.14
	Daily req. (mg)	0.96	0.06	1.09	0.11	1.08	0.11	0.41	0.05
	**Prob. adequacy**	**1.5%**	**9.8%**	**8.5%**	**23.3%**	**4.8%**	**15.6%**	**25.2%**	**38.0%**
**Vitamin B3 (Niacin)**	Daily intake (mg)	13.60	3.83	17.17	5.32	17.98	5.22	6.29	2.24
	Daily req. (mg)	12.29	1.84	11.83	1.77	12.31	1.85	4.87	0.73
	**Prob. adequacy**	**59.5%**	**35.8%**	**83.6%**	**27.5%**	**86.5%**	**24.3%**	**73.7%**	**33.0%**
**Vitamin B6 (Pyridoxine)**	Daily intake (mg)	1.00	0.30	1.24	0.40	1.30	0.46	0.50	0.18
	Daily req. (mg)	1.09	0.11	1.34	0.13	1.08	0.11	0.40	0.05
	**Prob. adequacy**	**39.9%**	**40.7%**	**39.7%**	**43.1%**	**65.2%**	**41.3%**	**69.8%**	**36.3%**
**Vitamin B12**	Daily intake (µg)	0.28	0.36	0.37	0.60	0.40	0.52	0.34	0.33
	Daily req. (µg)	2.04	0.20	2.14	0.20	2.00	0.20	0.77	0.10
	**Prob. adequacy**	**0.4%**	**3.8%**	**2.4%**	**14.7%**	**2.6%**	**13.8%**	**14.6%**	**32.3%**
**Vitamin C**	Daily intake (mg)	64.83	27.88	80.61	53.87	78.76	32.97	29.04	13.27
	Daily req. (mg)	36.29	3.77	44.37	5.41	37.50	3.75	25.00	2.50
	**Prob. adequacy**	**92.8%**	**19.4%**	**86.4%**	**24.6%**	**92.7%**	**24.3%**	**60.1%**	**44.2%**
**Vitamin E**	Daily intake (mg)	3.96	1.48	4.67	1.97	4.81	1.57	2.39	0.87
	Daily req. (mg)	6.02	0.73	6.14	0.63	5.77	0.87	3.66	0.46
	**Prob. adequacy**	**8.4%**	**20.0%**	**18.1%**	**27.0%**	**27.0%**	**32.4%**	**12.8%**	**27.5%**
**Folate**	Daily intake (µg)	343.0	123.7	417.4	171.3	460.5	175.5	151.7	78.7
	Daily req. (µg)	331.9	40.5	359.7	41.1	320.0	40.0	123.7	15.5
	**Prob. adequacy**	**47.0%**	**40.2%**	**59.3%**	**38.1%**	**75.8%**	**34.4%**	**62.5%**	**39.5%**
**Calcium**	Daily intake (mg)	285.1	95.2	389.2	185.5	432.1	212.2	162.7	68.7
	Daily req. (mg)	1034.9	101.9	842.6	80.6	833.3	83.3	430.6	43.1
	**Prob. adequacy**	**0.0%**	**0.0%**	**2.8%**	**13.1%**	**5.0%**	**19.9%**	**0.8%**	**7.9%**
**Iron**	Daily intake (mg)	12.75	3.73	16.55	5.41	18.83	5.92	5.65	2.24
	Daily req. (mg)	NA	NA	NA	NA	NA	NA	NA	NA
	**Prob. adequacy**	**4.4%**	**9.5%**	**7.3%**	**11.2%**	**29.9%**	**31.0%**	**9.4%**	**12.1%**
**Zinc**	Daily intake (mg)	7.41	2.14	9.28	2.85	10.41	2.88	3.51	1.29
	Daily req. (mg)	11.91	1.19	10.71	1.07	11.67	1.17	7.31	0.73
	**Prob. adequacy**	**5.9%**	**20.0%**	**37.3%**	**44.3%**	**31.1%**	**36.8%**	**1.7%**	**9.1%**

Table S7. Predicted changes in nutrient intake resulting from changes in pollination service

Percentage changes in nutrient intake (mean and standard deviation of all participants) are shown for three different scenarios of pollinator change: 1) loss of local pollinators, 2) ongoing declines in pollinator populations up to the year 2030, and 3) pollination enhancements. To test the sensitivity of our results to one of our model assumptions, we calculate values based on replacing lost food in the diet with either a) rice or c) potatoes of an equal number of calories (see Supplementary Method S1 for more information).

Nutrient	a) Rice replacement - local crops only						b) Rice replacement - local & imported crops						c) Potato replacement - local crops only					
	1) Poll. loss		2) Poll. decl.		3) Poll. incr.		1) Poll. loss		2) Poll. decl.		3) Poll. incr.		1) Poll. loss		2) Poll. decl.		3) Poll. incr.	
	Mean	*SD*	*Mean*	*SD*	*Mean*	*SD*	*Mean*	*SD*	*Mean*	*SD*	*Mean*	*SD*	*Mean*	*SD*	*Mean*	*SD*	*Mean*	*SD*
Folate	-19.2%	2.8%	-6.7%	1.0%	9.3%	1.5%	-21.0%	3.0%	-6.6%	1.0%	9.3%	1.5%	-15.9%	3.4%	-5.1%	1.1%	9.3%	1.5%
Calcium	-14.3%	4.9%	-5.0%	1.7%	5.5%	1.9%	-16.4%	5.3%	-4.8%	1.7%	5.4%	1.9%	-12.1%	4.6%	-3.8%	1.5%	5.4%	1.9%
Iron	-10.0%	2.6%	-3.5%	0.9%	4.7%	1.4%	-10.9%	2.9%	-3.4%	0.9%	4.7%	1.4%	-6.6%	2.3%	-2.1%	0.7%	4.7%	1.4%
Zinc	-2.4%	1.3%	-0.8%	0.5%	2.5%	0.8%	-0.6%	1.6%	-0.8%	0.5%	2.5%	0.8%	-1.9%	1.4%	-0.6%	0.5%	2.5%	0.8%
Vitamin B1	-5.8%	2.1%	-2.0%	0.7%	2.7%	0.9%	-5.9%	2.3%	-1.8%	0.7%	2.7%	0.9%	0.8%	1.6%	0.4%	0.5%	2.7%	0.9%
Vitamin B2	-8.8%	2.9%	-3.0%	1.0%	3.7%	1.2%	-9.2%	2.7%	-2.6%	0.9%	3.6%	1.2%	-9.4%	3.1%	-2.6%	0.9%	3.6%	1.2%
Vitamin B3	-1.1%	1.4%	-0.4%	0.4%	1.5%	0.5%	0.6%	1.7%	-0.4%	0.5%	1.5%	0.5%	5.1%	2.1%	1.6%	0.7%	1.5%	0.5%
Vitamin B6	-4.6%	2.0%	-1.6%	0.7%	2.0%	0.6%	-3.5%	2.2%	-1.2%	0.7%	2.0%	0.6%	4.1%	2.2%	1.6%	0.7%	2.0%	0.6%
Vitamin B12	0.0%	0.0%	0.0%	0.0%	0.0%	0.0%	0.0%	0.0%	0.0%	0.0%	0.0%	0.0%	0.0%	0.0%	0.0%	0.0%	0.0%	0.0%
Vitamin A	-21.3%	9.4%	-7.2%	3.1%	5.2%	2.2%	-23.9%	9.4%	-5.0%	2.3%	5.2%	2.2%	-22.9%	10.2%	-5.0%	2.3%	5.2%	2.2%
Vitamin C	-14.2%	7.6%	-4.7%	2.5%	2.4%	1.3%	-16.0%	8.2%	-3.9%	2.2%	2.4%	1.3%	37.9%	69.3%	12.8%	23.2%	2.4%	1.3%
Vitamin E	-3.6%	1.9%	-1.3%	0.6%	1.4%	0.6%	-19.8%	2.8%	-1.3%	0.7%	1.4%	0.6%	-2.4%	1.9%	-0.8%	0.6%	1.4%	0.6%

Table S8. Probability of adequacy by population subgroup and pollination scenario.

Probability of adequacy scores (mean and standard deviation) are shown for key micronutrients and each population subgroup under three pollination scenarios: 1) current pollination, 2) loss of local pollinators, and 3) optimum pollination. Mean probability of adequacy (MPA) represents the average probability of meeting estimated average requirements across calcium, zinc, vitamin C, vitamin B1 (thiamin), vitamin B2 (riboflavin), vitamin B3 (niacin), vitamin B6, folate (vitamin B9), vitamin A, vitamin B12, and iron, and provides a summary measure of overall micronutrient adequacy.

		Adolescent girl		Adult woman		Adult male		Under-five child	
**Nutrient**	**Scenario**	*Mean*	SD	*Mean*	SD	*Mean*	SD	*Mean*	SD
**Vitamin A**	Current pollination	1.6%	8.3%	5.2%	15.3%	4.2%	14.0%	5.0%	17.5%
	No pollination	0.7%	5.1%	3.2%	12.5%	2.0%	9.4%	4.3%	16.2%
	Optimum pollination	1.9%	9.5%	6.1%	16.3%	5.0%	15.3%	5.2%	18.0%
**Vitamin C**	Current pollination	92.8%	19.4%	86.4%	24.6%	92.7%	24.3%	60.1%	44.2%
	No pollination	85.7%	27.8%	79.3%	30.5%	87.6%	29.6%	48.5%	44.8%
	Optimum pollination	93.6%	18.4%	87.1%	23.9%	93.1%	23.8%	61.9%	43.7%
**Vitamin E**	Current pollination	8.4%	20.0%	18.1%	27.0%	27.0%	32.4%	12.8%	27.5%
	No pollination	6.4%	17.8%	14.9%	24.6%	22.9%	30.4%	11.9%	26.9%
	Optimum pollination	9.1%	20.8%	19.5%	28.0%	28.6%	33.1%	13.2%	27.8%
**Folate**	Current pollination	47.0%	40.2%	59.3%	38.1%	75.8%	34.4%	62.5%	39.5%
	No pollination	26.6%	37.1%	39.8%	41.1%	55.2%	43.0%	43.2%	42.2%
	Optimum pollination	57.6%	38.6%	68.0%	35.1%	83.4%	29.4%	70.8%	36.6%
**Calcium**	Current pollination	0.0%	0.0%	2.8%	13.1%	5.0%	19.9%	0.8%	7.9%
	No pollination	0.0%	0.0%	2.1%	11.7%	4.0%	17.9%	0.6%	7.0%
	Optimum pollination	0.0%	0.0%	3.2%	14.1%	5.6%	20.7%	1.0%	8.5%
**Iron**	Current pollination	4.4%	9.5%	7.3%	11.2%	29.9%	31.0%	9.4%	12.1%
	No pollination	2.1%	5.8%	4.3%	8.1%	20.0%	27.1%	6.7%	9.6%
	Optimum pollination	5.8%	11.1%	9.2%	12.9%	34.7%	32.7%	10.2%	12.7%
**MPA**	Current pollination	26.5%	15.0%	36.1%	17.8%	41.7%	17.6%	33.6%	19.5%
	No pollination	22.0%	14.3%	31.6%	17.6%	36.2%	18.0%	29.1%	19.6%
	Optimum pollination	28.6%	15.1%	38.1%	17.7%	44.0%	17.6%	35.3%	19.2%

Table S9. Summary of models relating species network metrics to nutritional importance

Summary of linear models evaluating the relationship between pollinator nutritional importance (quantified as the summed proportional decline in nutrient intake following pollinator removal) and either pollinator abundance alone or abundance plus one Z-standardised species-level network metric. Network metrics were calculated on the pooled plant–pollinator metaweb and standardised using abundance-constrained null models (see Methods). Metrics tested include interaction selectivity (d′), crop focus (proportion of visits directed to crop species), interaction breadth (degree), weighted closeness centrality, and interaction evenness (Shannon diversity). Models are ranked by Akaike Information Criterion (AIC), with lower AIC indicating better fit. Adj. R^2 denotes adjusted R-squared; ΔAdj. R^2 indicates change relative to the abundance-only model; Metric coefficient gives the slope estimate for the added network metric; Metric p-value gives its associated significance; No. of predictors indicates the number of fixed effects included. Across all models, network metrics explained little additional variation in nutritional importance beyond pollinator abundance.

Model description	Network metric included	No. of predictors	AIC	Adj. R2	Δ Adj. R2	Metric coefficient	Metric p-value
Abundance only	-	1	637.915	0.418	0.000	NA	NA
Abundance plus z-crop focus	Crop focus	2	638.200	0.421	0.003	0.074	0.195
Abundance plus z-d'	Blüthgen’s d′	2	638.702	0.419	0.001	0.024	0.276
Abundance plus z-degree	Degree	2	639.168	0.417	-0.001	-0.034	0.393
Abundance plus z-diversity	Shannon diversity	2	639.351	0.417	-0.002	-0.013	0.457
Abundance plus z-closeness	Closeness centrality	2	639.735	0.415	-0.003	0.012	0.674
Abundance plus all predictors	All	6	643.247	0.417	-0.002	NA	NA

Table S10. Non-crop plant species Indirect Contributions (IC) to each key micronutrient

By supporting crop pollinators with pollen and nectar, non-crop plant species provide an indirect contribution (IC) to the pollination service underpinning micronutrient production. Values show the IC score of each non-crop plant species for each key micronutrient, as well as the mean score across all six nutrients. These IC scores represent the percentage contribution to the supply of each micronutrient that is provided by the plant species (via their support of key pollinators; Table S3). These top-30 plants are listed in order of most to least important and represent promising targets for pollination management interventions on farmland. For village-specific values, see Fig. S5c.

Plant species	Calcium	Folate	Iron	Vit. A	Vit. C	Vit. E	Mean
Persicaria nepalensis	1.73%	1.99%	1.21%	2.26%	1.24%	0.76%	1.53%
Tagetes erecta	0.87%	1.25%	0.70%	1.14%	0.51%	0.53%	0.83%
Cotoneaster microphyllus	0.85%	0.93%	0.57%	1.26%	0.71%	0.34%	0.78%
Cirsium wallichii	0.60%	1.28%	0.62%	0.46%	0.14%	0.55%	0.61%
Rosa sericea	0.65%	0.80%	0.47%	0.92%	0.50%	0.28%	0.60%
Galinsoga ciliata	0.61%	0.79%	0.46%	0.79%	0.42%	0.26%	0.55%
Cosmos bipinnatus	0.50%	0.96%	0.49%	0.46%	0.17%	0.31%	0.48%
Impatiens sp.01	0.49%	1.03%	0.50%	0.37%	0.13%	0.26%	0.46%
Spiraea canescens	0.34%	0.37%	0.23%	0.50%	0.27%	0.14%	0.31%
Potentilla argyrophylla	0.29%	0.38%	0.23%	0.49%	0.24%	0.13%	0.29%
Thymus linearis	0.30%	0.31%	0.19%	0.43%	0.25%	0.12%	0.27%
Stachys sp.	0.23%	0.44%	0.22%	0.20%	0.08%	0.19%	0.23%
Cirsium verutum	0.18%	0.36%	0.17%	0.17%	0.05%	0.22%	0.19%
Impatiens balsamina	0.19%	0.39%	0.20%	0.17%	0.06%	0.13%	0.19%
Erysimum hieracifolium	0.19%	0.20%	0.13%	0.30%	0.18%	0.08%	0.18%
Cynoglosum zeylanium	0.20%	0.20%	0.13%	0.29%	0.16%	0.08%	0.18%
Taraxacum sp.	0.19%	0.21%	0.13%	0.28%	0.18%	0.07%	0.18%
Anemone sp.	0.17%	0.20%	0.12%	0.29%	0.15%	0.09%	0.17%
Anisomeles indica	0.16%	0.31%	0.15%	0.16%	0.06%	0.08%	0.15%
Dipsacus inermis	0.15%	0.27%	0.14%	0.16%	0.06%	0.14%	0.15%
Euphorbia sikkimensis	0.17%	0.15%	0.11%	0.27%	0.12%	0.07%	0.15%
Lonicera myrtillus	0.15%	0.20%	0.12%	0.19%	0.09%	0.07%	0.14%
Salvia nubicola	0.13%	0.26%	0.12%	0.11%	0.03%	0.14%	0.13%
Commelina paludosa	0.14%	0.28%	0.14%	0.11%	0.04%	0.09%	0.13%
Nicotiana rustica	0.13%	0.19%	0.10%	0.14%	0.07%	0.06%	0.12%
Nepeta leucophylla	0.12%	0.17%	0.09%	0.16%	0.08%	0.06%	0.11%
Dahlia sp.	0.11%	0.14%	0.08%	0.13%	0.08%	0.05%	0.10%
Verbascum thapsus	0.11%	0.19%	0.10%	0.07%	0.03%	0.04%	0.09%
Berberis aristata	0.09%	0.13%	0.07%	0.11%	0.07%	0.04%	0.08%
Anaphalis royleana	0.09%	0.16%	0.08%	0.07%	0.04%	0.03%	0.08%

Table S11. Characteristics of the study population in Jumla, Nepal.

Characteristics of the study population in Jumla, Nepal, based on the household enrolment survey and farmer questionnaire. Household size is the number of usual residents. Household head literacy is the self-reported ability to read and write. Caste categories are self-identified. Landholding size denotes total agricultural land owned by the household (hectares). Income from agriculture is the share of total household income (cash and in-kind) attributed to agricultural activities. The lead farmer is the primary agricultural decision-maker named by the household. Seed-sourcing categories are mutually exclusive and sum to 100%. Cooking variables capture the primary stove type and primary fuel used.

**Demographic information**	**Value**
Number of households in the study population	200
Number of individuals in the study population	776
Mean household size (number of occupants)	6.73
Median household size (number of occupants)	6
Mean years of schooling for household head	1.8
Percentage of household heads who are literate (able to read & write)	40.0%
**Caste information**	
Percentage of households from Matwali Chhetri caste	90.0%
Percentage of households from Newari caste	0.5%
Percentage of households from Janajati caste	3.3%
Percentage of households from Dalit caste	6.2%
**Agricultural characteristics**	
Mean landholding size (hectares)	0.2
Mean percentage of household income derived from agriculture	64.6%
Mean age of lead farmer (years)	38
Percentage of lead farmers who are women	57.5%
Mean number of crops cultivated per household	18.9
Percentage of households sourcing seeds from own crops	97.4%
Percentage of households sourcing seeds from neighbour's crops	2.6%
Percentage of households sourcing seeds from external sources	0.0%
**Cooking information**	
Percentage of households cooking on metal stove with chimney	100.0%
Percentage of households using firewood as main source of heat	100.0%

Table S12. Annual production of key crops and crop groups in Jumla District

Values show annual production during the period of crop-pollinator surveys (2021–22) and the period of dietary recall surveys (2022–23), with percentage difference in production between years. Production (measured in metric tonnes, Mt) remained broadly consistent across years, with a mean interannual variation of 3.4% (maximum 14%). Source: Government of Nepal, Ministry of Agriculture and Livestock Development (2024), Statistical Information on Nepalese Agriculture, 2078/79 (2021/22) and 2079/80 (2022/23).

Crop	2021-22 Production (Mt)	2022-23 Production (Mt)	% difference 2021-22 Production (Mt)
Potato	43,227	39,170	-10.36%
Jumli beans	2,608	3,020	13.64%
Cereal crops	26,425	30,110	12.24%
Apple	13,958	14,351	2.74%
Broadleaved mustard	148	139	-6.47%
Fresh vegetables	4,024	4,405	8.65%
		**Mean % difference:**	**3.41%**

Supporting figures

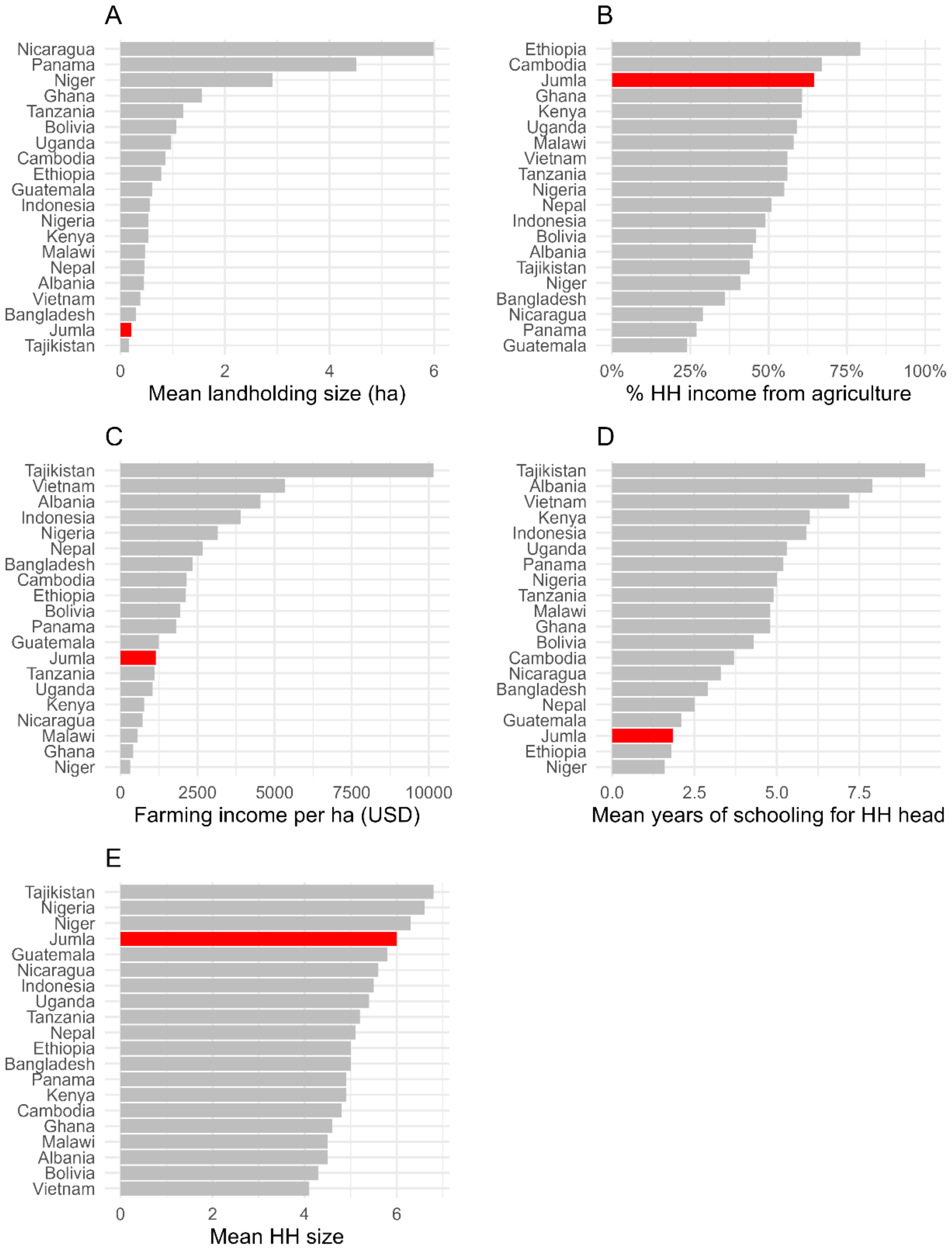

Figure S1. Comparing smallholder farmers in Jumla with other regions of the world

We compare the smallholder households (HH) in our study region of Jumla District (n=200 households) against smallholder households in 19 countries where the UN Food & Agricultural Organisation (FAO) have collected data on key demographic, socioeconomic and agricultural metrics to compile a global data portrait of smallholder farmers (FAO 2017). The comparisons indicate that Jumla District is relatively representative of many other smallholder farming regions of the world based on a) mean landholding size; b) percentage of household income derived from agriculture; c) farming income per hectare of land; d) mean years of schooling for the household head; e) mean family (household) size.

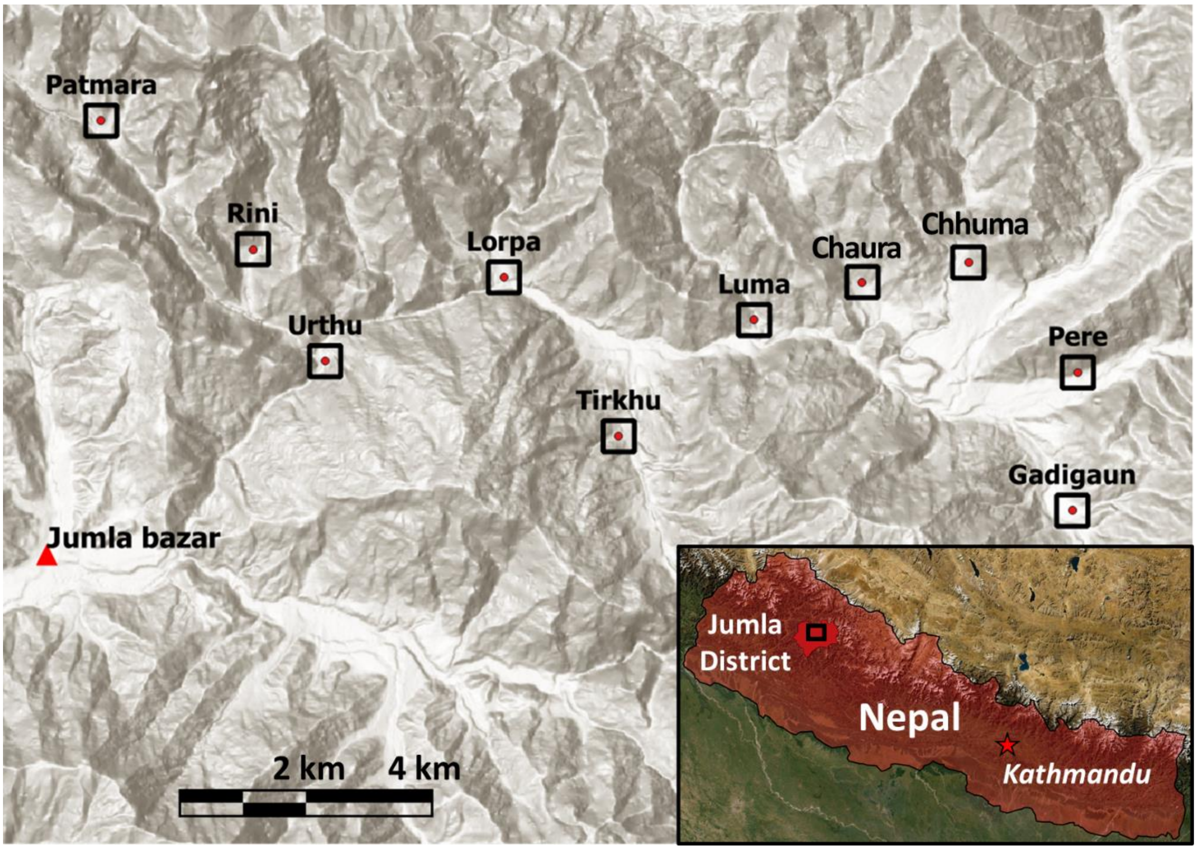

Figure S2. Map of study village locations in Patarasi Rural Municipality, Jumla

Map showing the location of the 10 study villages in relation to the district headquarters, Jumla bazar. Red points mark the midpoint of each village, and the black boxes show the 600 x 600 metre study area, into which nine 60 x 60 metre survey plots were placed. The inlay shows the location of Jumla District (marked in red) within Nepal, with the area of the map marked with a black rectangle. **Basemap imagery**: Esri World Imagery. Source: Esri, Maxar, Earthstar Geographics, and the GIS User Community.

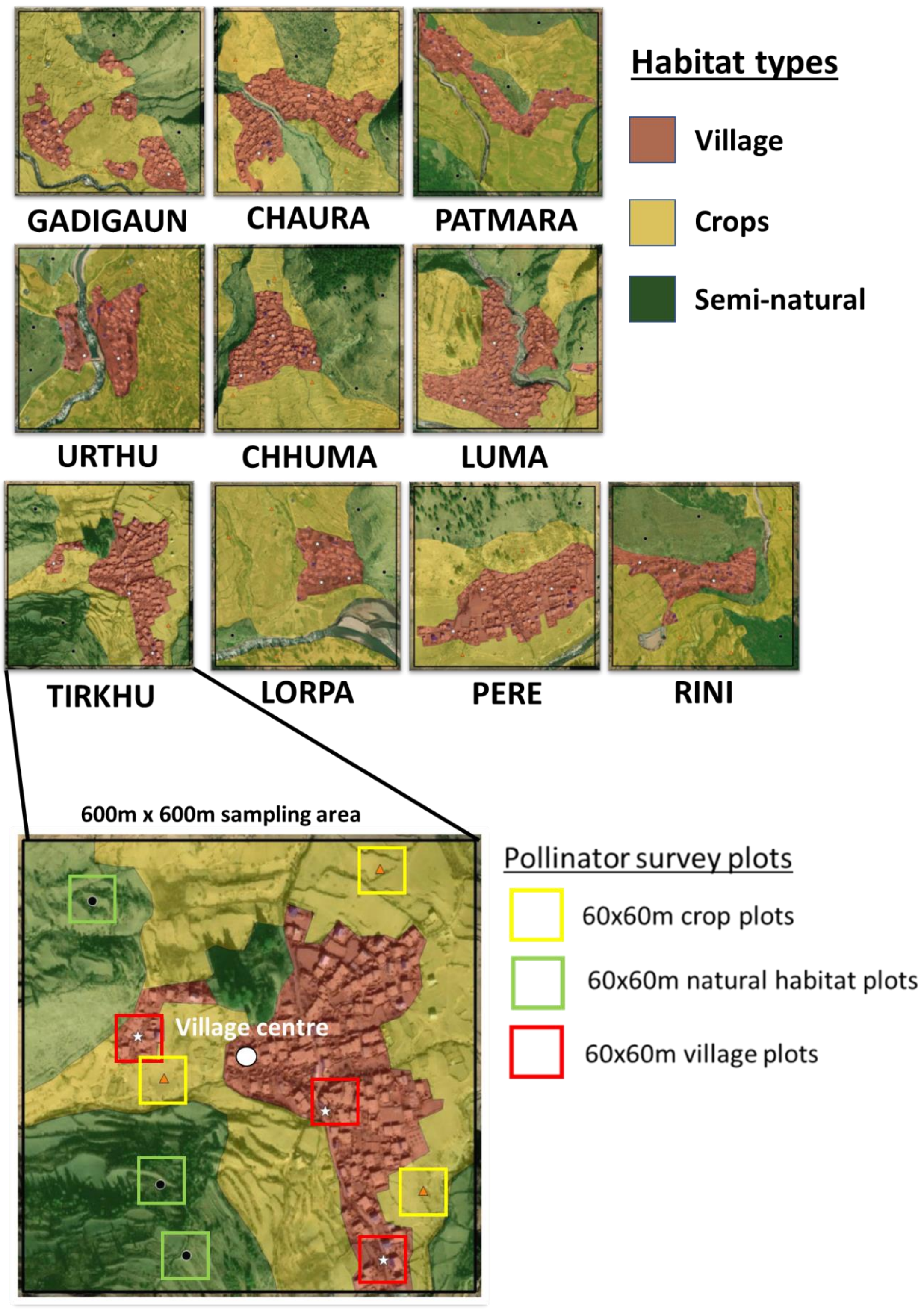

Figure S3. Habitat composition of study villages

Habitat composition of the ten 600 x 600 metre sampling areas centred the midpoint of each village. The sampling area of Tirkhu village is enlarged to demonstrate the random placement of 60 x 60 metre survey plots within each habitat type. **Basemap imagery**: Esri World Imagery. Source: Esri, Maxar, Earthstar Geographics, and the GIS User Community.

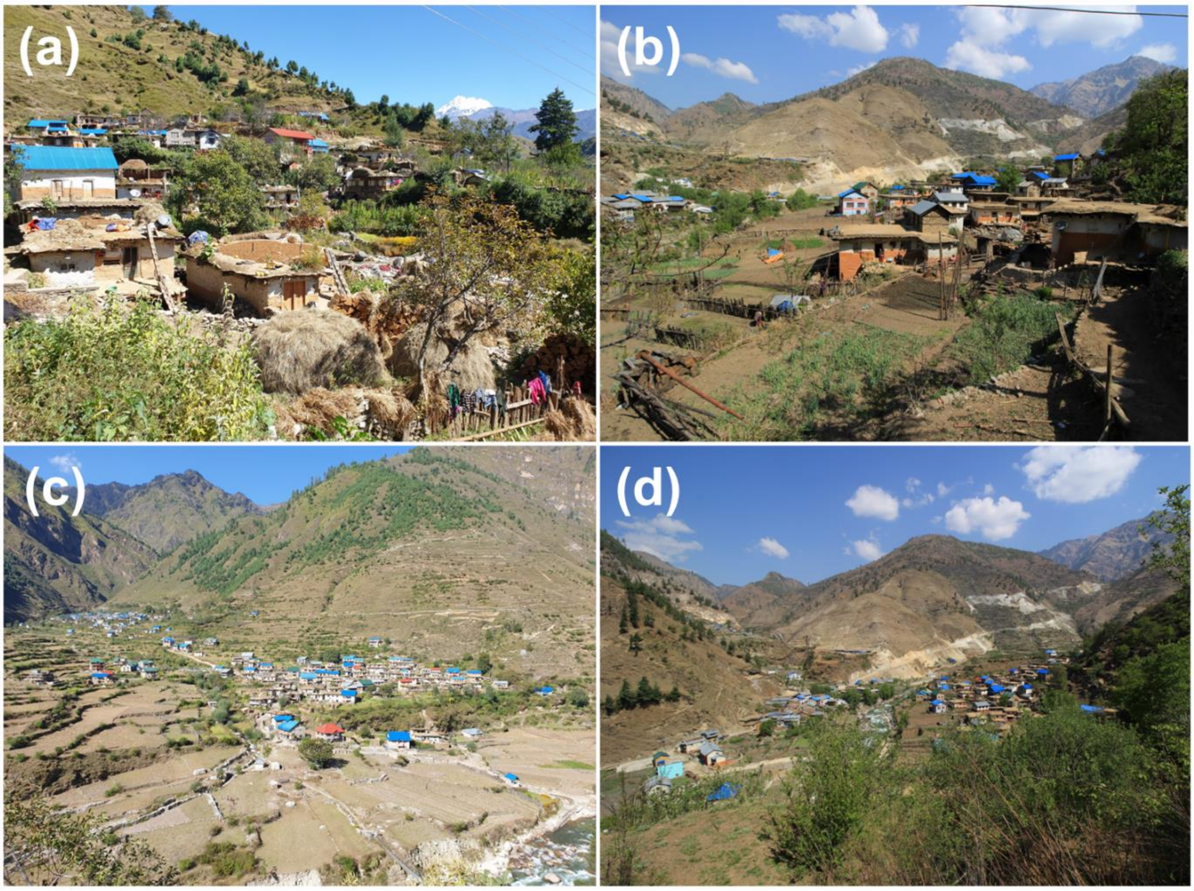

Figure S4: Photographs of study sites

Images of households and vegetable gardens (a-b), along with the surrounding crop fields and semi-natural habitat (c-d) taken from three representative study villages. Image A was taken in October during the harvest season (credit: author Naomi Saville) while images B-D were taken during the start of the growing season in April (credit: author Thomas Timberlake).

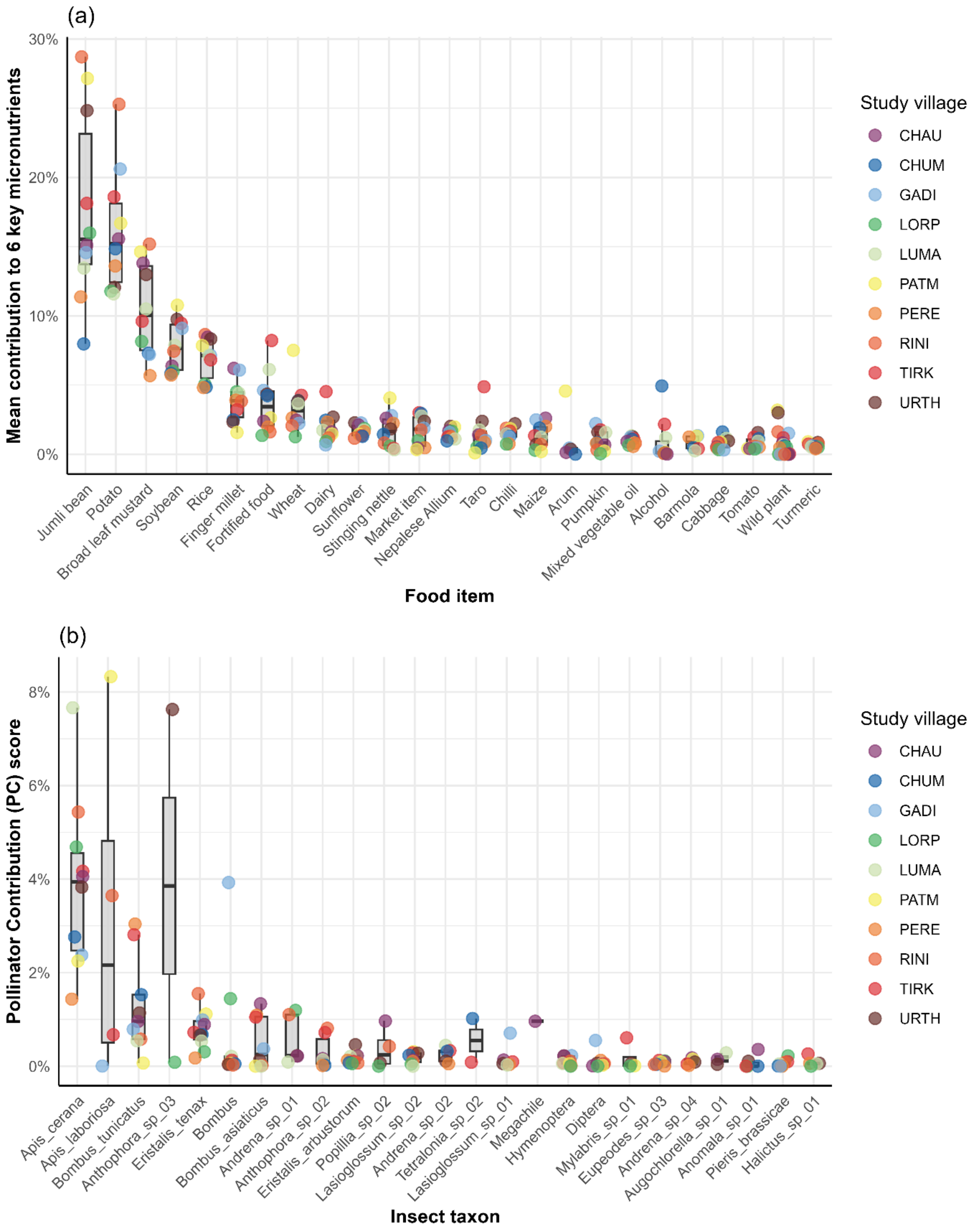
(a)
Mean contribution to 6 key micronutrients
30%
20%
10%
0%
Jumli bean
Potato
Broad leaf mustard
Soybean
Rice
Finger millet
Fortified food
Wheat
Dairy
Sunflower
Stinging nettle
Market item
Nepalese Allium
Taro
Chilli
Maize
Arum
Pumpkin
Mixed vegetable oil
Alcohol
Barmola
Cabbage
Tomato
Wild plant
Turmeric
Food item
Study village
CHAU
CHUM
GADI
LORP
LUMA
PATM
PERE
RINI
TIRK
URTH
(b)
Pollinator Contribution (PC) score
8%
6%
4%
2%
0%
Apis_cerana
Apis_laboriosa
Bombus_tunicatus
Anthophora_sp_03
Eristalis_tenax
Bombus
Bombus_asiaticus
Andrena_sp_01
Anthophora_sp_02
Eristalis_arbustorum
Popillia_sp_02
Lasioglossum_sp_02
Andrena_sp_02
Tetralonia_sp_02
Lasioglossum_sp_01
Megachile
Hymenoptera
Diptera
Mylabris_sp_01
Eupeodes_sp_03
Andrena_sp_04
Augochlorella_sp_01
Anomala_sp_01
Pieris_brassicae
Halictus_sp_01
Insect taxon
Study village
CHAU
CHUM
GADI
LORP
LUMA
PATM
PERE
RINI
TIRK
URTH

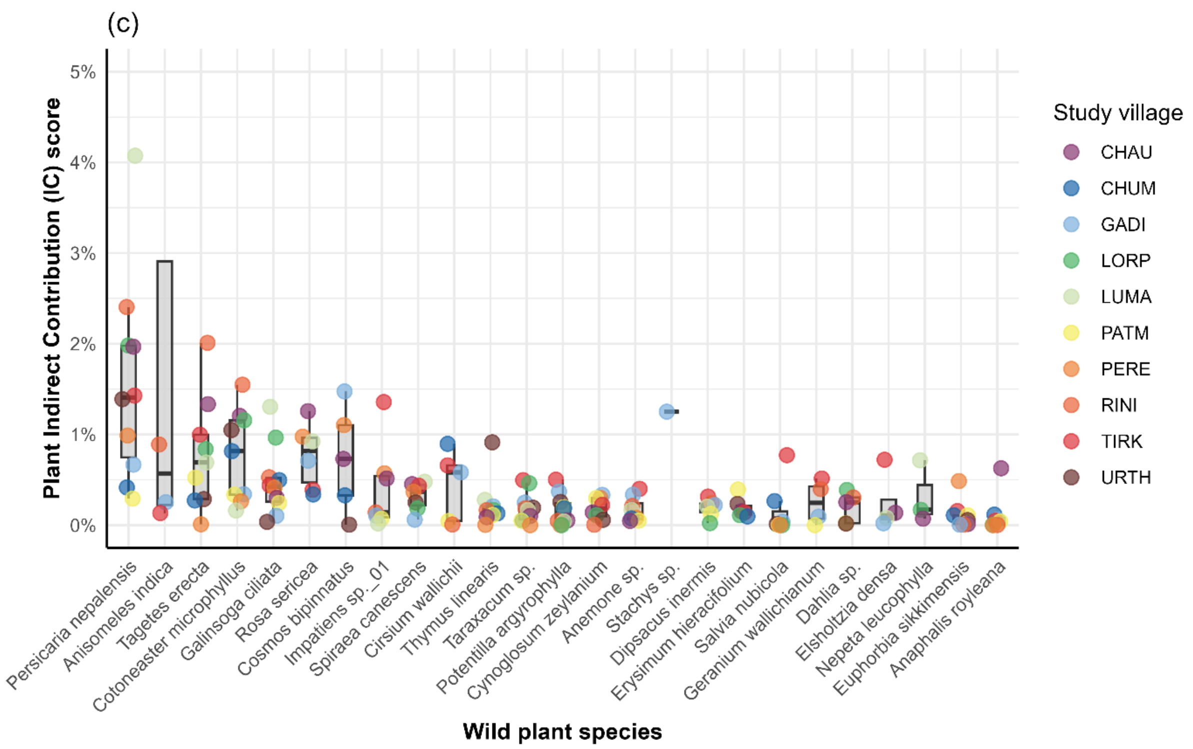

Figure S5. Village-level importance of crops, pollinators and wild plants

Figures show the contributions of the 25 most important a) crops; b) insect pollinators; and c) wild plants to the supply of six key pollinator-dependent micronutrients (calcium, iron, vitamin C, folate, vitamin A, and vitamin E) in each village. For crops (panel a), the values show the mean percentage contribution of each crop to the total intake of all six micronutrient. For pollinators (panel b), the values show the Pollinator Contribution (PC) scores of each insect to the production of these six micronutrients. For plants (panel c), the values show each plant's Indirect Contribution (IC) to the pollination service of these six micronutrients. See the main text for the equations that generate each score. For all panels, boxplots summarise variation across villages: boxes indicate the interquartile range (25th–75th percentiles), central lines show medians, whiskers extend to 1.5 × the interquartile range, and coloured points represent individual village-level values. Analyses are based on plant–pollinator interaction records collected across all study villages (n = 10 villages).

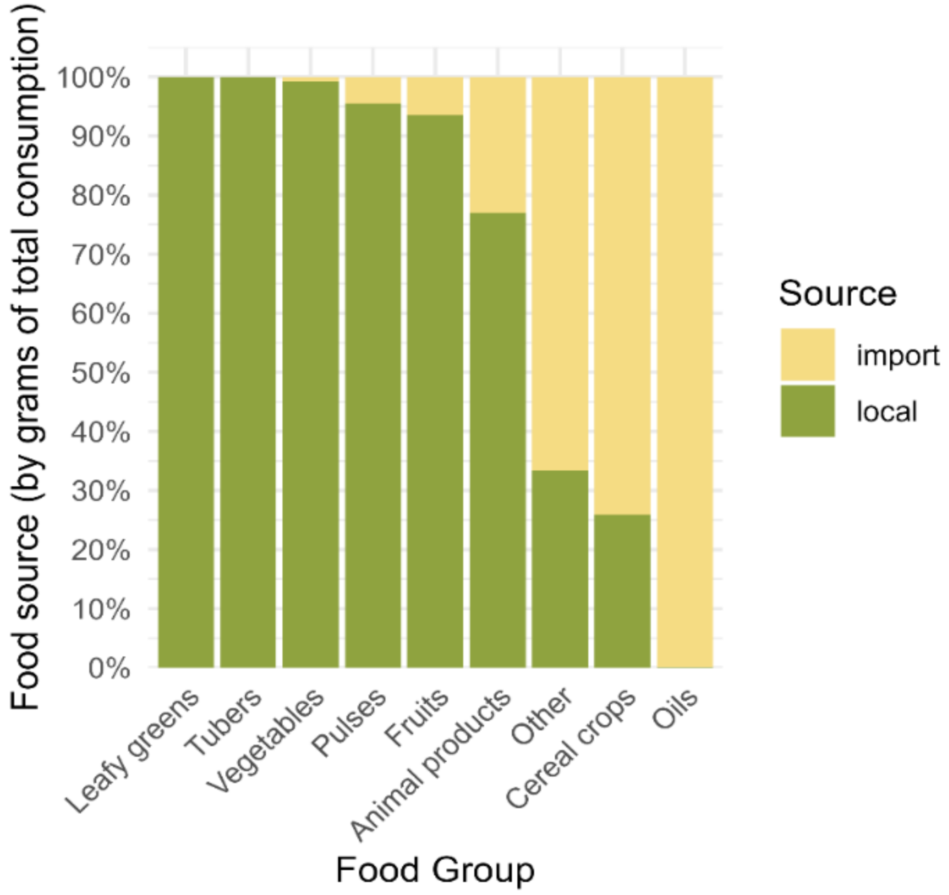

Figure S6. Source of key food groups

Stacked bars show the share of total household consumption (by mass) derived from local production (green) versus foods that are imported from outside of Jumla District (yellow) across nine major food groups. Percentages represent total intake pooled across all study participants (n = 776 individuals). For most nutritionally important and largely pollinator-dependent food groups (leafy greens, vegetables, pulses, fruits), over 90% of consumption comes from local production, underscoring the minimal role of markets in supplying these foods. In contrast, cereals (primarily polished rice) and cooking oils are the only foods routinely imported in significant quantities.

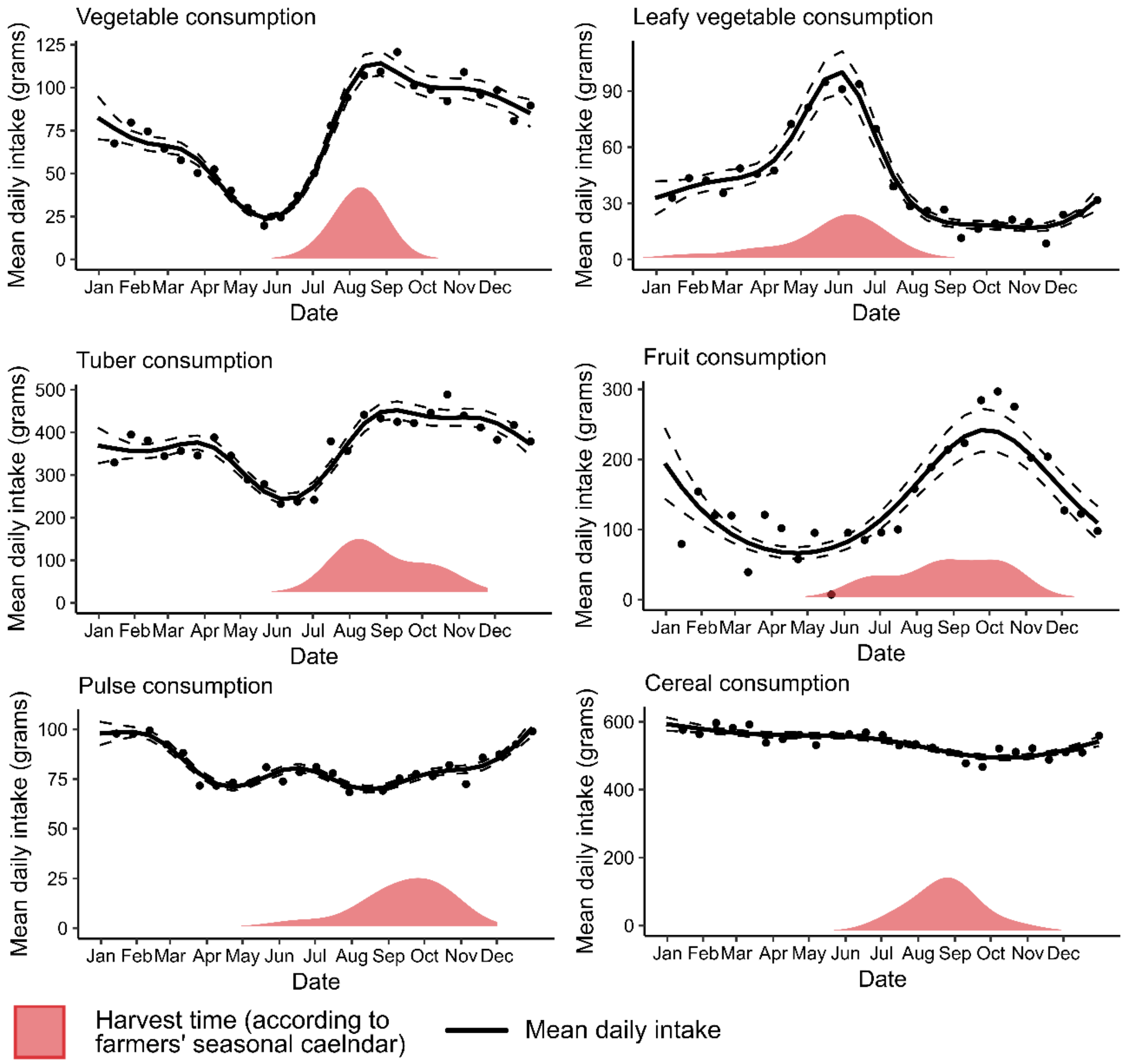

Figure S7. Seasonal patterns in mean daily intake of major food groups

Seasonality in the consumption of key food groups by adults and adolescent girls in Jumla District, Nepal (n = 591 individuals; children excluded). Each point represents the mean daily intake during a single sampling round (fortnightly intervals) pooled across all non-child respondents. Solid lines show smoothed trends fitted using a Generalized Additive Model (GAM) with 95% confidence intervals (dashed lines). Shaded red areas indicate the main harvest periods for each food group based on farmers' seasonal calendars. Food groups are ordered from most seasonal (top left) to least seasonal (bottom right) based on the amplitude of their consumption peaks (percentage difference between maximum and minimum intake values across the year). Strong seasonal fluctuations are evident for most locally grown food groups (vegetables, leafy vegetables, tubers, pulses and fruits), with sharp post-harvest peaks followed by declines, reflecting tight constraints of local availability. In contrast, cereal intake remains relatively constant throughout the year, consistent with its status as one of the few routinely imported foods.

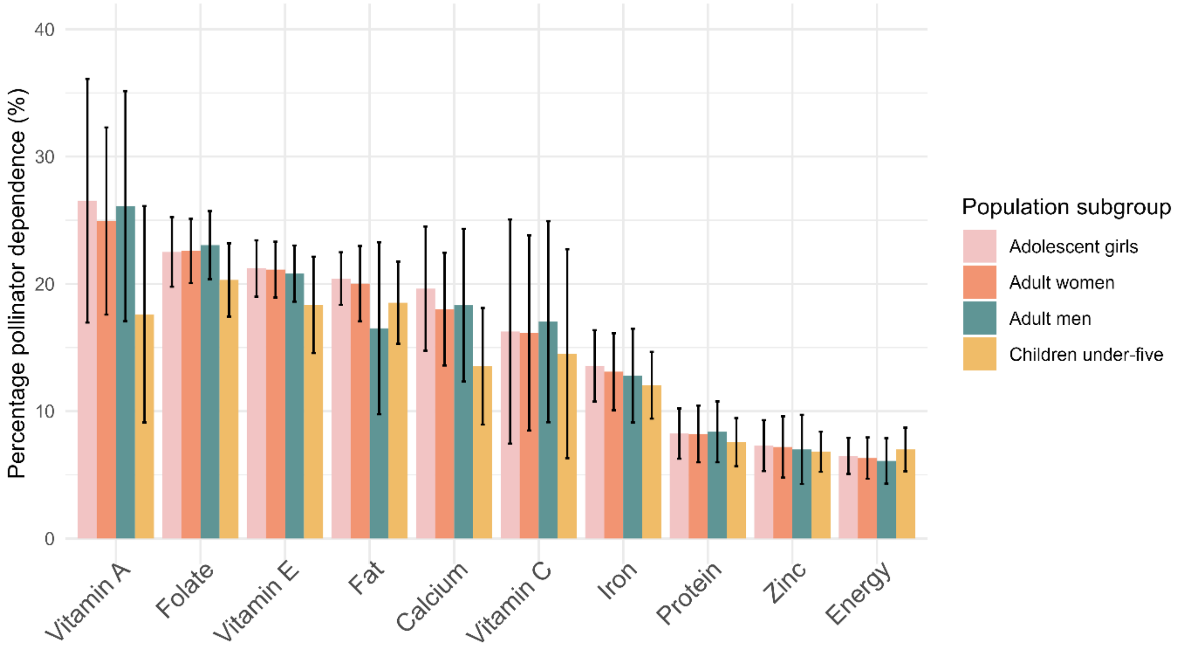

Figure S8. Pollinator dependence of key nutrients by population subgroup

The proportion of each key nutrient that is directly attributable to insect pollinators is shown for each population subgroup: adolescent females (n = 190 individuals), adult females (n = 215 individuals), adult males (n = 186 individuals), and children under five years (n = 185 individuals). Values show the mean ± standard deviation of all individuals within each population subgroup and are based on their total dietary intake across the 12-month study period. Pollinator dependence values include foods grown outside of Jumla and imported into the region (i.e. reliant on pollinators in other regions), as well as foods that depend on insect pollinators for seed production, as farmers in this region rely on their own seeds for cultivation in subsequent seasons.

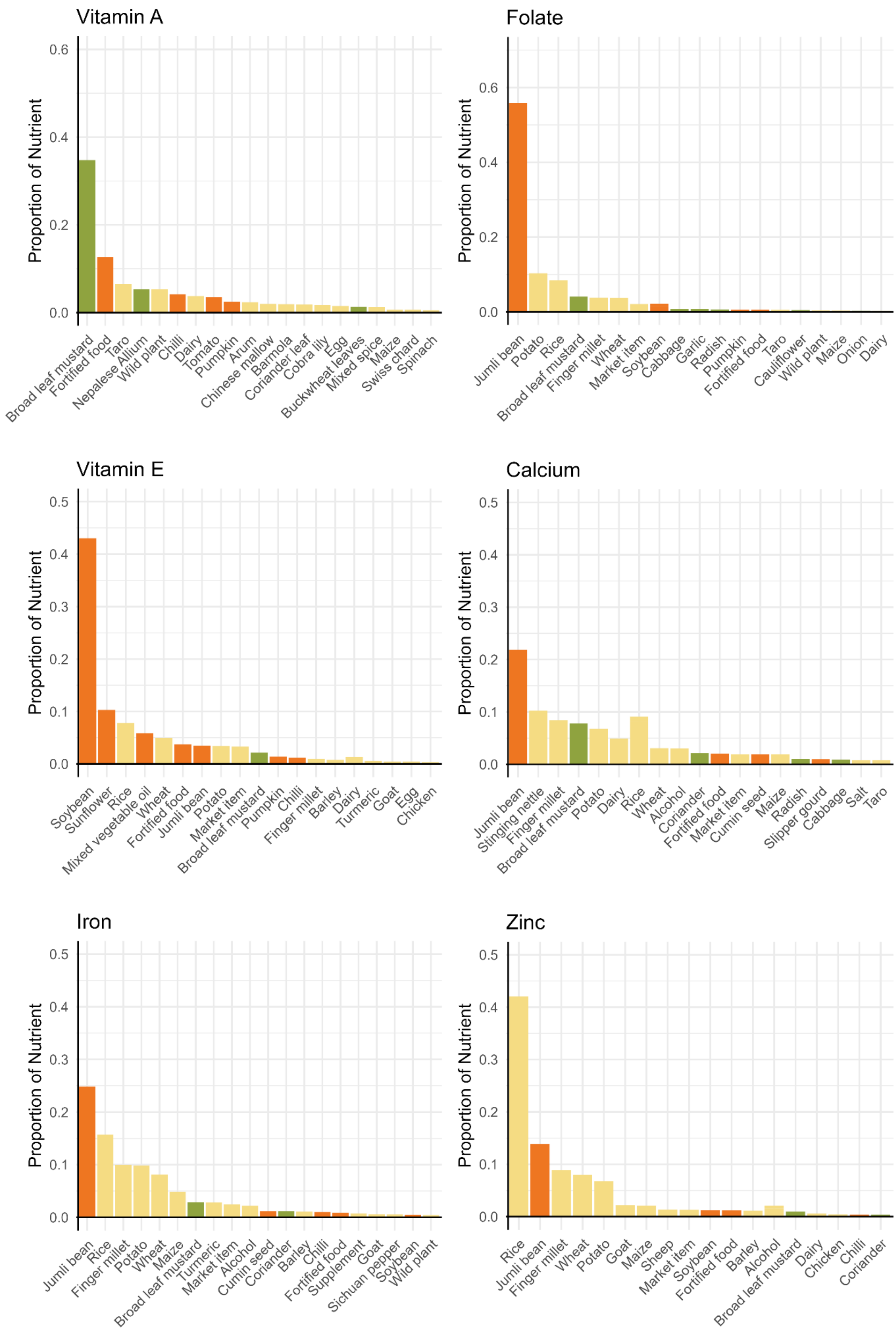
Vitamin A
Proportion of Nutrient
0.6
0.4
0.2
0.0
Broad leaf mustard
Fortified food
Taro
Nepalese Allium
Wild plant
Chilli
Dairy
Tomato
Pumpkin
Arum
Chinese mallow
Barmola
Coriander leaf
Cobra lily
Egg
Buckwheat leaves
Mixed spice
Maize
Swiss chard
Spinach
Folate
Proportion of Nutrient
0.6
0.4
0.2
0.0
Jumli bean
Potato
Rice
Broad leaf mustard
Finger millet
Wheat
Market item
Soybean
Cabbage
Garlic
Radish
Pumpkin
Fortified food
Taro
Cauliflower
Wild plant
Maize
Onion
Dairy
Vitamin E
Proportion of Nutrient
0.5
0.4
0.3
0.2
0.1
0.0
Soybean
Sunflower
Rice
Mixed vegetable oil
Wheat
Fortified food
Jumli bean
Potato
Market item
Broad leaf mustard
Pumpkin
Chilli
Finger millet
Barley
Dairy
Turmeric
Goat
Egg
Chicken
Calcium
Proportion of Nutrient
0.5
0.4
0.3
0.2
0.1
0.0
Jumli bean
Stinging nettle
Finger millet
Broad leaf mustard
Potato
Dairy
Rice
Wheat
Alcohol
Coriander
Fortified food
Market item
Cumin seed
Maize
Radish
Slipper gourd
Cabbage
Salt
Taro
Iron
Proportion of Nutrient
0.5
0.4
0.3
0.2
0.1
0.0
Jumli bean
Rice
Finger millet
Potato
Wheat
Maize
Broad leaf mustard
Turmeric
Market item
Alcohol
Cumin seed
Coriander
Barley
Chilli
Fortified food
Supplement
Goat
Sichuan pepper
Soybean
Wild plant
Zinc
Proportion of Nutrient
0.5
0.4
0.3
0.2
0.1
0.0
Rice
Jumli bean
Finger millet
Wheat
Potato
Goat
Maize
Sheep
Market item
Soybean
Fortified food
Barley
Alcohol
Broad leaf mustard
Dairy
Chicken
Chilli
Coriander

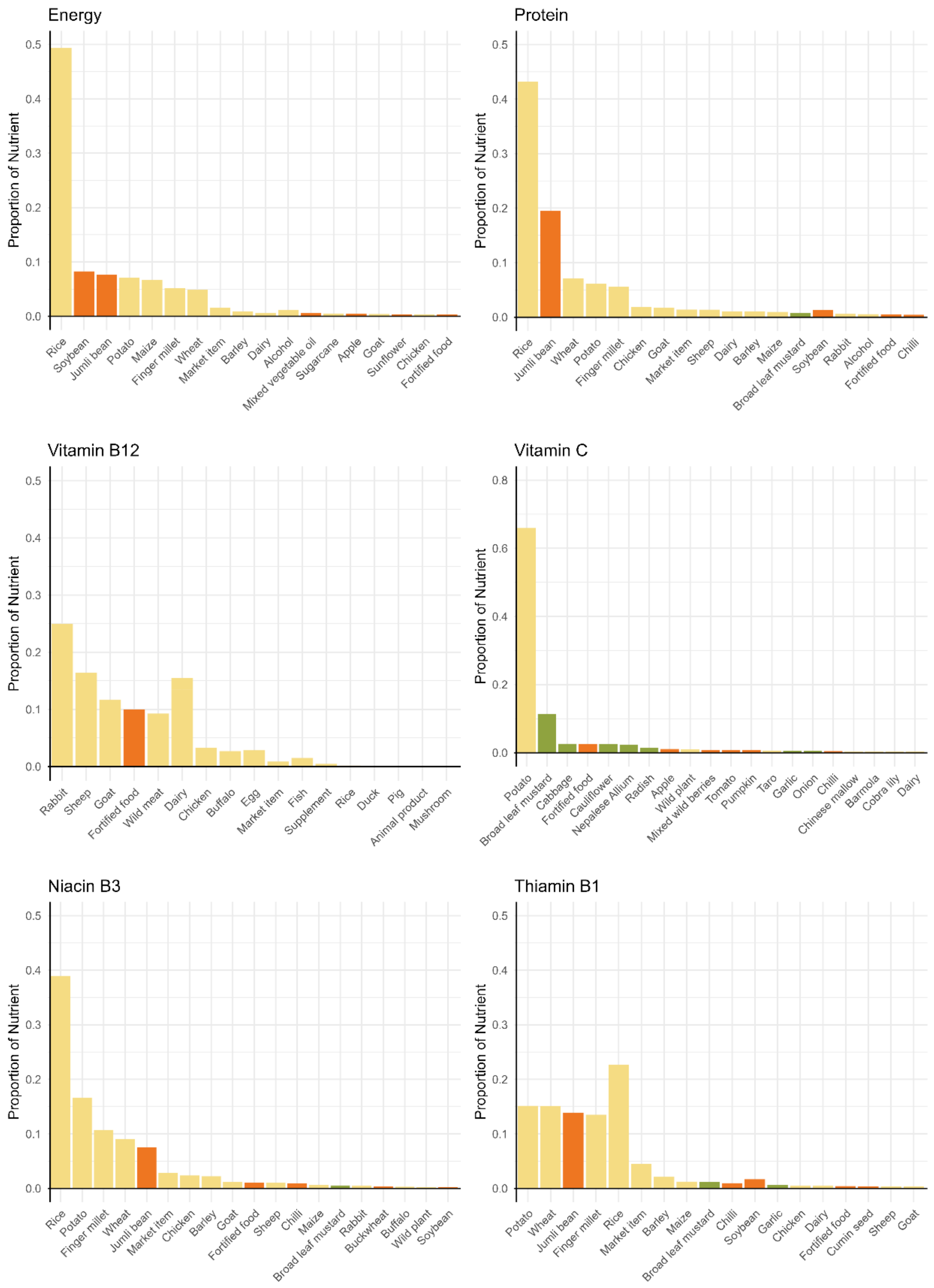

Figure S9. Contribution of different foods to intake of key micronutrients

Proportion of each key dietary nutrient provided by different food ingredients. Values represent the proportion of total nutrient intake pooled across all study participants (n = 776 individuals) over the 12-month study period that is contributed by each ingredient. Ingredients shown in orange are crops whose yield is directly dependent on insect pollination; those in green rely on insect pollinators for seed production; and those in yellow are not dependent on animal pollination.

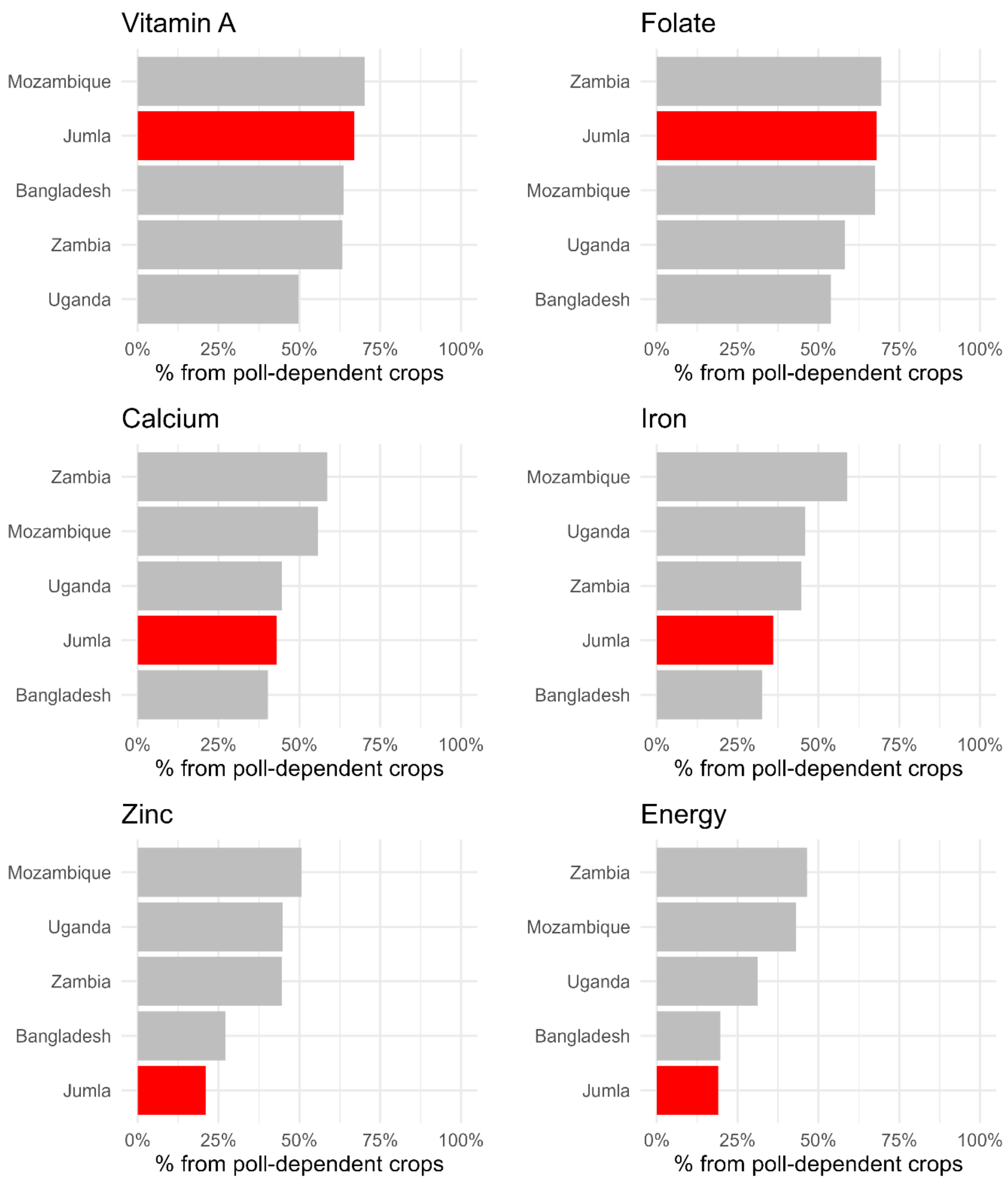

Figure S10. Comparing reliance on pollinator-dependent crops with other countries

We compare our results from Jumla District (red) with those from smallholder populations in four other countries taken from the only previous study to quantify the pollinator-dependence of people's diets (Ellis *et al.* 2015). For six key nutrients, we compared the percentage of people's dietary intake that was derived from pollinator-dependent crops. The comparisons show a high degree of similarity in people's nutritional reliance on pollinator-dependent crops.

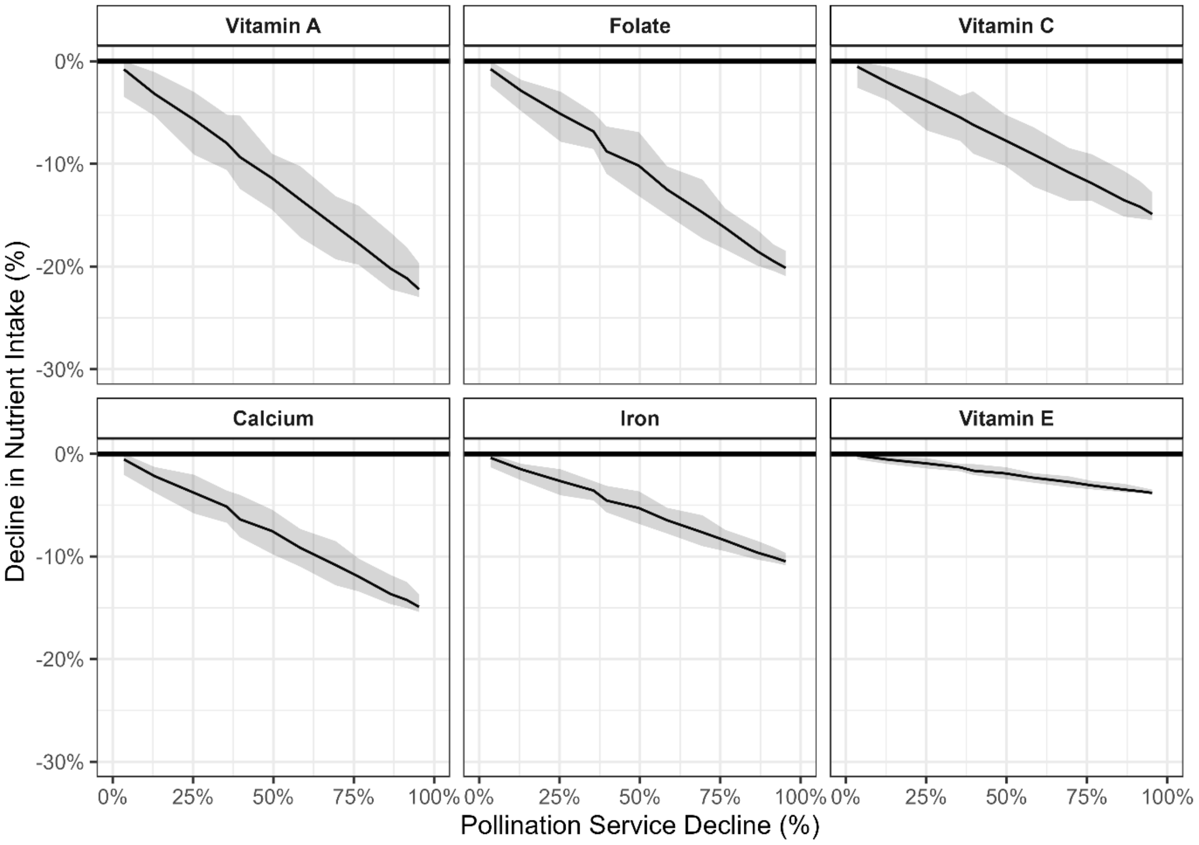

Figure S11. Simulated impacts of pollination decline on nutrient intake

Each panel shows the predicted percentage decline in population-level intake of six key pollinator-dependent nutrients (vitamin A, folate, vitamin C, calcium, iron, and vitamin E,) across a gradient of pollinator population decline (0%–100%). Pollinator decline is simulated in 10% increments, with response diversity incorporated by drawing individual species' decline rates from a normal distribution with a mean equal to the target decline and a standard deviation of 0.2. Results represent the mean and 95% confidence intervals (shaded ribbons) from repeated simulation runs (100 simulations per scenario) across n = 776 individuals.

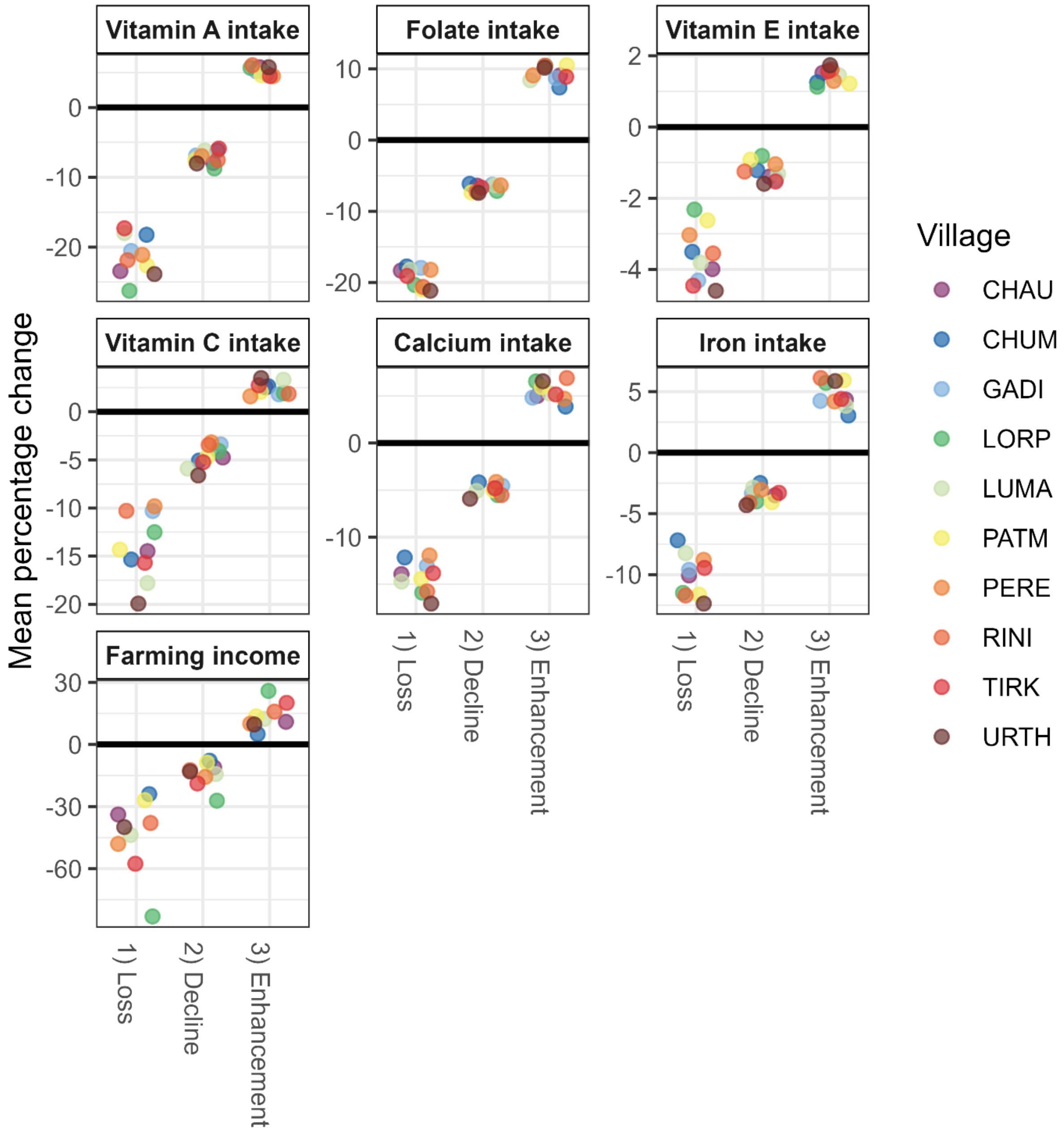

Figure S12. Village-level changes in micronutrient intake and farming income

Points show the percentage changes in micronutrient intake and farming income that are predicted to result from three different scenarios of change in the pollination service that were modelled in this study: 1) complete loss of local pollinators; 2) ongoing declines in pollinator abundance up to the year 2030; and 3) pollination enhancement, whereby crop pollination deficits are removed. Each point shows the mean percentage change for all participants within each of the 10 study villages (n=10 villages). For more details on the model assumptions and caveats, see Supplementary Method S1.

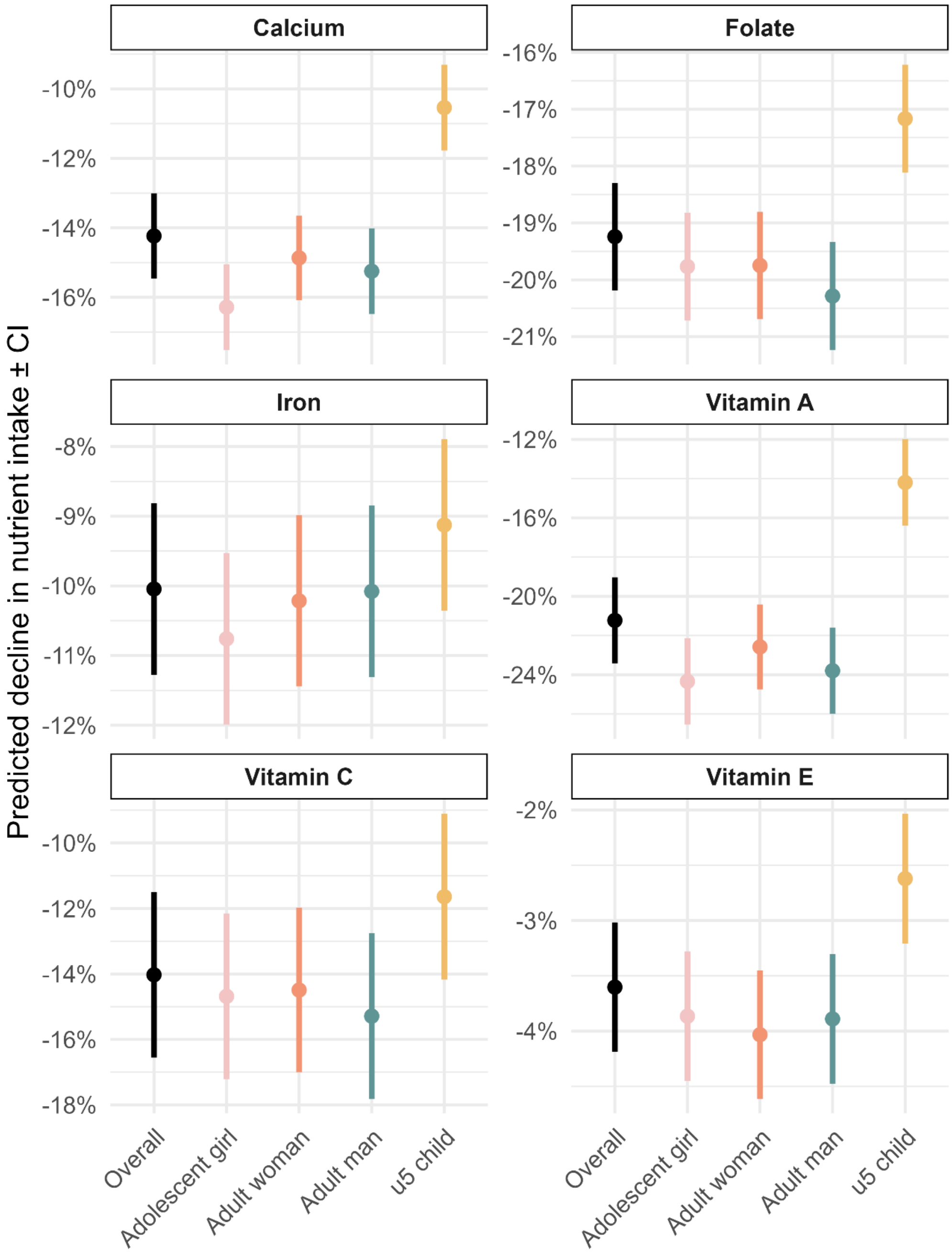

Figure S13. Predicted declines in nutrient intake by population subgroup

Predicted percentage decline (mean ± 95% CI) in dietary intake of six key nutrients (calcium, folate, iron, vitamins A, C, and E) under a scenario of total pollinator loss. Estimates were derived from linear mixed-effects models with demographic subgroup as a fixed effect and village as a random effect, followed by marginal means estimated using the *emmeans* package. Results are shown for the overall population (in black) and for key demographic subgroups (adolescent girls, adult women, adult men, and children under five). Analyses are based on n = 776 individuals comprising 215 adult women, 186 adult men, 190 adolescent girls, and 185 children under five years.

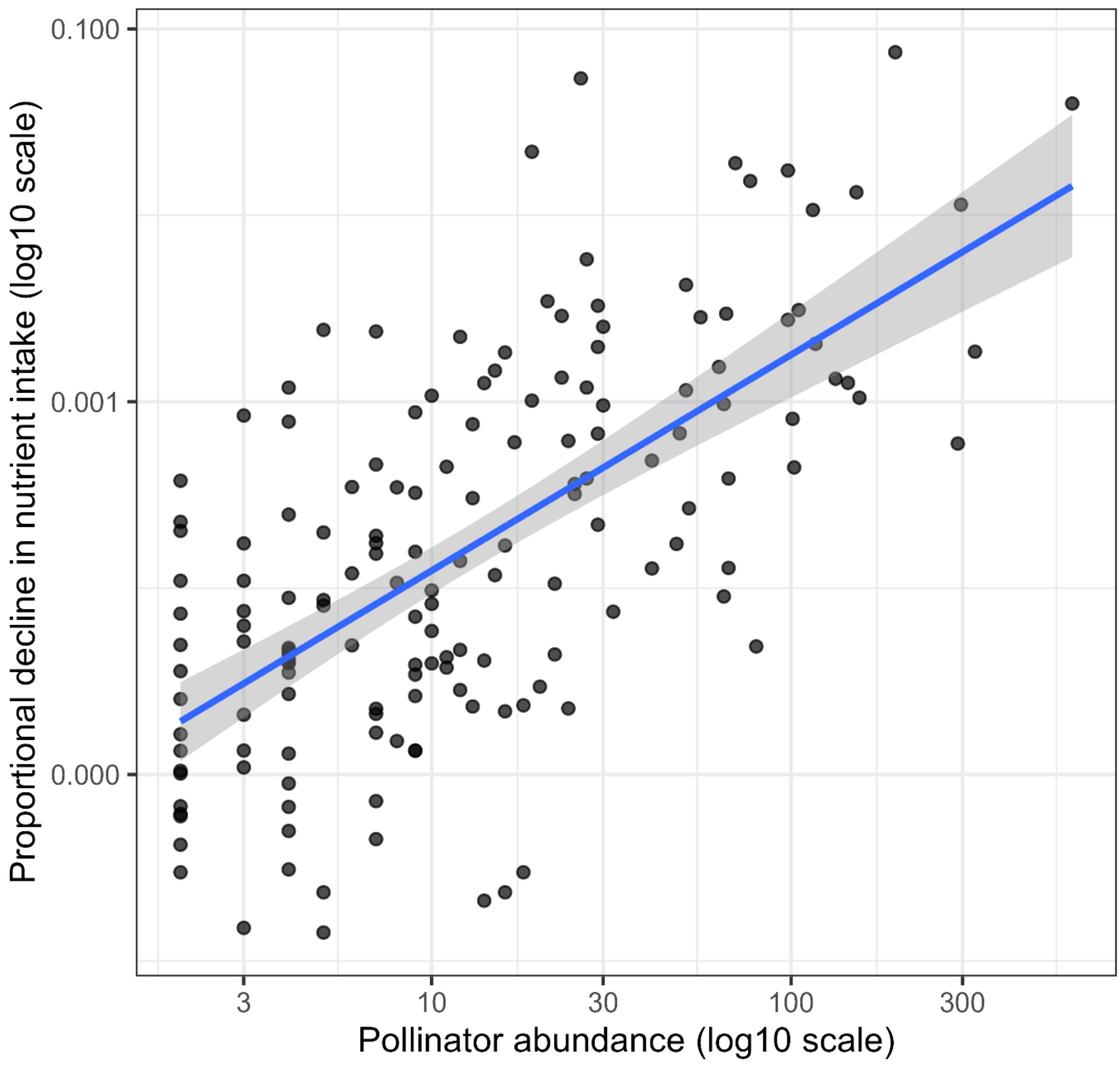

Figure S14. Relationship between pollinator abundance and nutritional importance

Each point represents a pollinator taxon in the plant–pollinator network (n = 185 taxa), showing the association between species abundance (total plant visits; $\log_{10}$ scale) and the proportional decline in nutrient intake resulting from its removal. Nutritional importance is calculated as the summed proportional reduction in intake across six key pollinator-dependent nutrients (vitamin A, folate, vitamin E, calcium, vitamin C and iron). Taxa lacking a species-level OTU identification were excluded, as species-level network metrics could not be calculated for these records. Pollinator abundance was the only species attribute strongly associated with nutritional importance (linear model: $R^2 = 0.42$, $t_{183} = 12.63$, $p < 2 \times 10^{-16}$). No species-level network metrics (degree, interaction diversity, specialisation d′, closeness centrality or crop focus) provided significant additional explanatory power once abundance was accounted for (Table S9). The blue line shows the fitted linear regression with 95% confidence intervals (shaded).

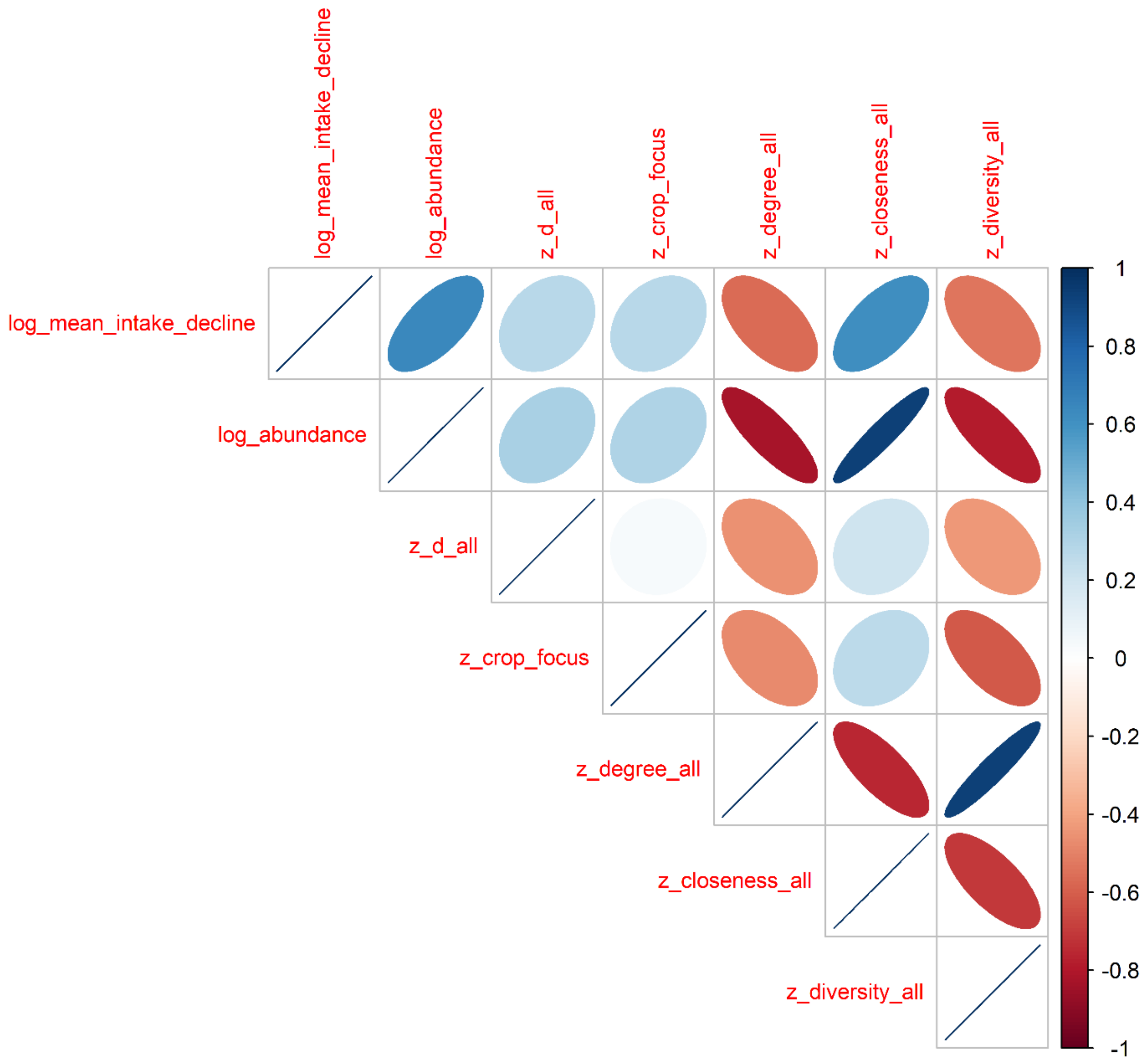

Figure S15. Correlations amongst species-level network metrics

Pairwise correlations among a pollinator's nutritional importance (log mean intake decline), abundance (log transformed) and Z-standardised species-level network metrics calculated from the pooled plant–pollinator metaweb. Network metrics were converted to Z-scores based on abundance-constrained null models that preserve each pollinator's total number of visits while randomising the distribution of visits across plant species (see Methods). Metrics shown include interaction selectivity (Blüthgen's d′), crop focus (proportion of visits directed to crop species), interaction breadth (degree), weighted closeness centrality, and interaction evenness (Shannon diversity). Ellipses indicate the direction and strength of correlations (blue = positive, red = negative). Despite null-standardisation, several network metrics remain correlated with abundance, indicating that abundant pollinators tend to exhibit non-random interaction structure. However, as shown in the main analysis (Table S9), these network descriptors explain little additional variation in nutritional importance beyond pollinator abundance. Correlations were calculated across n = 185 pollinator taxa.

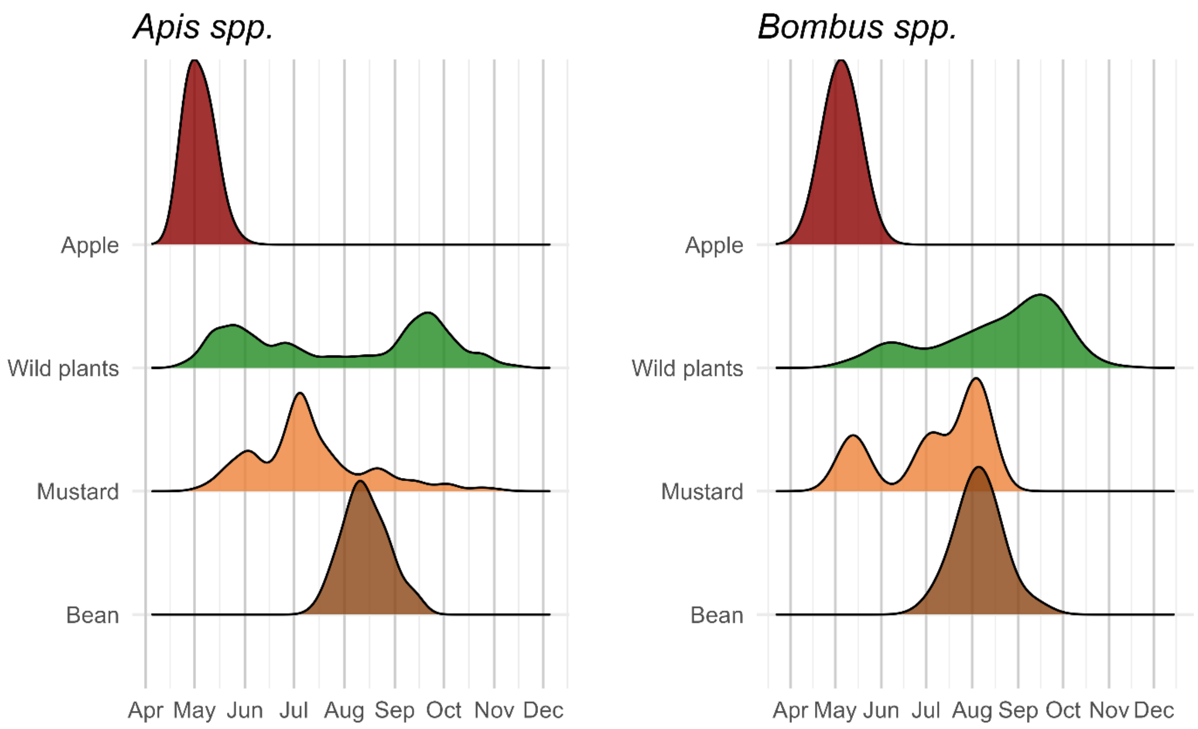

Figure S16. Seasonal patterns of crop and wild plant visitation

Ridgeline plots show the frequency of flower visits by the two most important crop pollinator groups in the region—honeybees (*Apis* spp.; n = 3,639 visits) and bumblebees (*Bombus* spp.; n = 466 visits)—recorded across 10 study villages (n = 10 villages). Plots show visits to the three most nutritionally and economically important pollinator-dependent crops and to wild plants. Colours indicate floral resource: apple (red), wild plants (green), mustard (gold) and bean (brown). For both honeybees and bumblebees, visits to wild plants increase outside the main crop-flowering periods (particularly September–December), indicating that pollinators shift onto wild flora to maintain floral resource supply when crops are no longer flowering. This pattern suggests limited competition between crops and wild plants for pollinators, and instead indicates that wild plants help sustain populations of key pollinators throughout the year, thereby facilitating the pollination of crop plants.

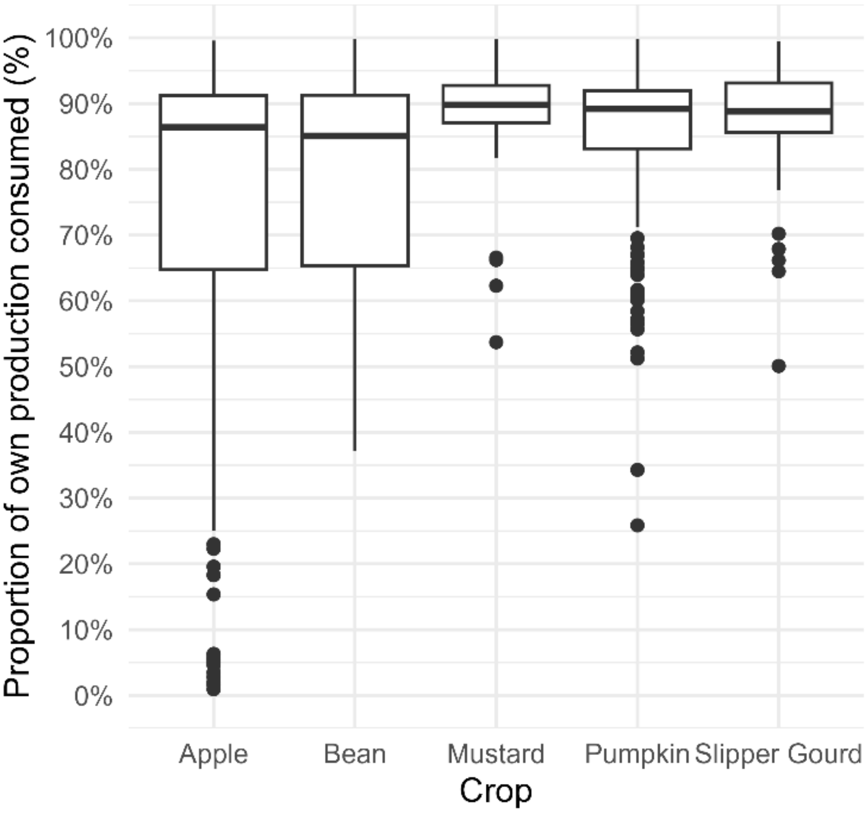

Figure S17. Proportion of household crop production consumed by household members

Boxplots show the proportion of total harvest consumed by the producing household (rather than sold, traded, gifted, or fed to animals) for five key pollinator-dependent crops grown in Jumla, Nepal (apple, bean, mustard, pumpkin, and slipper gourd). Data are based on surveys of lead farmers from n=200 study households. Across all crops, households reported consuming more than 85% of their own production, highlighting Jumla's highly subsistence-oriented food system with limited market channels for storage, trade, or export. Boxes indicate the interquartile range (25th–75th percentiles), the central line shows the median, whiskers extend to 1.5 × the interquartile range, and points denote individual households outside this range.

References

Aldercotte, A.H., Simpson, D.T. & Winfree, R. (2022). Crop visitation by wild bees declines over an 8-year time series: A dramatic trend, or just dramatic between-year variation? *Insect Conserv Diver*.

Bartomeus, I., Potts, S.G., Steffan-Dewenter, I., Vaissière, B.E., Woyciechowski, M., Krewenka, K.M. *et al.* (2014). Contribution of insect pollinators to crop yield and quality varies with agricultural intensification. *Peerj*, 2, e328.

Blaauw, B.R. & Isaacs, R. (2014). Flower plantings increase wild bee abundance and the pollination services provided to a pollination-dependent crop. *J Appl Ecol*, 51, 890-898.

Carriquiry, A.L. (2003). Estimation of usual intake distributions of nutrients and foods. *The Journal of nutrition*, 133, 601S-608S.

Dainese, M., Martin, E.A., Aizen, M.A., Albrecht, M., Bartomeus, I., Bommarco, R. *et al.* (2019). A global synthesis reveals biodiversity-mediated benefits for crop production. *Science Advances*, 5, eaax0121.

Ellis, A.M., Myers, S.S. & Ricketts, T.H. (2015). Do Pollinators Contribute to Nutritional Health? *Plos One*, 10.

FAO (2017). A data portrait of smallholder farmers. Food and Agriculture Organisation of the United Nations Rome.

Földesi, R., Howlett, B.G., Grass, I. & Batáry, P. (2021). Larger pollinators deposit more pollen on stigmas across multiple plant species—A meta-analysis. *J Appl Ecol*, 58, 699-707.

Gaire, K., Beilin, R. & Miller, F. (2015). Withdrawing, resisting, maintaining and adapting: food security and vulnerability in Jumla, Nepal. *Regional Environmental Change*, 15, 1667-1678.

Garibaldi, L.A., Carvalheiro, L.G., Vaissiere, B.E., Gemmill-Herren, B., Hipolito, J., Freitas, B.M. *et al.* (2016). Mutually beneficial pollinator diversity and crop yield outcomes in small and large farms. *Science*, 351, 388-391.

Government of Nepal (2012). *Food Composition Table for Nepal 2012*. Government of Nepal, Ministry of Agriculture Development, Department of Food Technology Quality Control, National Nutrition Program, Kathmandu.

Harris-Fry, H., Beard, B.J., Harrisson, T., Paudel, P., Shrestha, N., Jha, S. *et al.* (2018). Smartphone tool to collect repeated 24 h dietary recall data in Nepal. *Public Health Nutr*, 21, 260-272.

Hotz, C. (2007). Dietary indicators for assessing the adequacy of population zinc intakes. *Food and Nutrition Bulletin*, 28, S430–S453.

Howlett, B., Walker, M., Rader, R., Butler, R., Newstrom-Lloyd, L. & Teulon, D. (2011). Can insect body pollen counts be used to estimate pollen deposition on pak choi stigmas. *New Zealand Plant Protection*, 64, 25-31.

Institute of Medicine (2001). *Dietary Reference Intakes for Vitamin A, Vitamin K, Arsenic, Boron, Chromium, Copper, Iodine, Iron, Manganese, Molybdenum, Nickel, Silicon, Vanadium, and Zinc*. The National Academies Press, Washington, DC.

Institute of Medicine (2011). *Dietary Reference Intakes for Calcium and Vitamin D*. The National Academies Press, Washington, DC.

King, C., Ballantyne, G. & Willmer, P.G. (2013). Why flower visitation is a poor proxy for pollination: measuring single-visit pollen deposition, with implications for pollination networks and conservation. *Methods Ecol Evol*, 4, 811-818.

Kipnis, V., Midthune, D., Buckman, D.W., Dodd, K.W., Guenther, P.M., Krebs-Smith, S.M. *et al.* (2009). Modeling Data with Excess Zeros and Measurement Error: Application to Evaluating Relationships between Episodically Consumed Foods and Health Outcomes. *Biometrics*, 65, 1003-1010.

Klein, A.M., Vaissiere, B.E., Cane, J.H., Steffan-Dewenter, I., Cunningham, S.A., Kremen, C. & Tscharntke, T. (2007). Importance of pollinators in changing landscapes for world crops. *P Roy Soc B-Biol Sci*, 274, 303-313.

Kortsch, S., Timberlake, T.P., Cirtwill, A.R., Sapkota, S., Rokoya, M., Devkota, K. *et al.* (2024). Decline in Honeybees and Its Consequences for Beekeepers and Crop Pollination in Western Nepal. *Insects*, 15, 281.

Longvah, T., Ananthan, R., Bhaskarachary, K. & Venkaiah, K. (2017). *Indian Food Composition Tables 2017*. National Institute of Nutrition, Indian Council of Medical Research, Department of Health Research, Ministry of Health and Family Welfare, Government of India, Hyderabad.

Lonsdorf, E., Kremen, C., Ricketts, T., Winfree, R., Williams, N. & Greenleaf, S. (2009). Modelling pollination services across agricultural landscapes. *Ann Bot-London*, 103, 1589-1600.

Memmott, J., Timberlake, T.P., Baral, S., Bhandari, L., Bhusal, D.R., Bohara, S. *et al.* (2024). Ecological, dietary, and socio-economic data from 10 smallholder farming villages in Jumla District, Nepal, 2021-2022. NERC EDS Environmental Information Data Centre.

Millard, J., Outhwaite, C.L., Ceausu, S., Carvalheiro, L.G., da Silva e Silva, F.D., Dicks, L.V. *et al.* (2023). Key tropical crops at risk from pollinator loss due to climate change and land use. *Science Advances*.

Ministry of Health and Population, N. (2018). *Nepal National Micronutrient Status Survey Report 2016*. Government of Nepal Ministry of Health and Population, UNICEF, USAID, European Union, CDC, New Era Kathmandu.

Müller, J., Hothorn, T., Yuan, Y., Seibold, S., Mitesser, O., Rothacher, J. *et al.* (2023). Weather explains the decline and rise of insect biomass over 34 years. *Nature*.

National Research Council (1986). The Probability Approach. In: *Nutrient adequacy: Assessment using food consumption surveys*. National Academies Press (US).

Nyambose, J., Tucker, K.L. & Koski, K.G. (2002). High intra/interindividual variance ratios for energy and nutrient intakes of pregnant women in rural Malawi show that many days are required to estimate usual intake. *The Journal of nutrition*, 132, 1313-1318.

Public Health England (2021). *McCance and Widdowson's The Composition of Foods Integrated Dataset*. Public Health England, London.

Quinlan, G.M., Miller, D.A.W. & Grozinger, C.M. (2023). Examining spatial and temporal drivers of pollinator nutritional resources: evidence from five decades of honey bee colony productivity data. *Environmental Research Letters*, 18, 114018.

Roulston, T.H. & Goodell, K. (2011). The Role of Resources and Risks in Regulating Wild Bee Populations. *Annual Review of Entomology, Vol 56*, 56, 293-312.

Shaheen, N., Rahim, A.T.M.A., Mohiduzzaman, M., Banu, C.P., Bari, M.L., Tukun, A.B. *et al.* (2013). *Food Composition Table for Bangladesh 2013*. Institute of Nutrition and Food Science, Centre for Advanced Research in Sciences, University of Dhaka, Dhaka.

Silva, F.D.S., Carvalheiro, L.G., Aguirre-Gutiérrez, J., Lucotte, M., Guidoni-Martins, K. & Mertens, F. (2021). Virtual pollination trade uncovers global dependence on biodiversity of developing countries. *Science Advances*, 7, eabe6636.

Smith, M.R., Mueller, N.D., Springmann, M., Sulser, T.B., Garibaldi, L.A., Gerber, J. *et al.* (2022). Pollinator Deficits, Food Consumption, and Consequences for Human Health: A Modeling Study. *Environ Health Perspect*, 130, 127003.

Smith, M.R., Singh, G.M., Arian, D.M. & Myers, S.S. (2015). Effects of decreases of animal pollinators on human nutrition and global health: a modelling analysis. *Lancet*, 386, 1964-1972.

Thapa, R., Ghimire, S., Bhattarai, P., Acharya, S., Poudel Chhetri, B. & Kushma Tharu, R. (2024). A Comprehensive Assessment of Apple Production in Jumla District, Nepal: Status, Economics,

Marketing and Challenges. *Turkish Journal of Agriculture - Food Science and Technology*, 12, 159-178.

U.S. Department of Agriculture, A.R.S. (2007). *USDA Table of Nutrient Retention Factors, Release 6*. U.S. Department of Agriculture, Beltsville, MD.

U.S. Department of Agriculture, A.R.S. (2013). *USDA National Nutrient Database for Standard Reference, Release 25 (SR25)*. U.S. Department of Agriculture, Beltsville, MD.

WHO (2006). *WHO Child Growth Standards: Length/height-for-age, weight-for-age, weight-for-length, weight-for-height and body mass index-for-age: Methods and development*. World Health Organization, Geneva.

WHO (2007). *WHO Reference 2007: Growth reference data for children and adolescents 5–19 years*. World Health Organization, Geneva.

WHO & FAO (2004). *Vitamin and mineral requirements in human nutrition*. World Health Organization & Food and Agriculture Organisation, Bankok, Thailand.

WHO/FAO (2001). *Human Vitamin and Mineral Requirements: Report of a Joint FAO/WHO Expert Consultation*. World Health Organization and Food and Agriculture Organization of the United Nations, Bangkok, Thailand and Rome, Italy.

Woodcock, B.A., Garratt, M.P.D., Powney, G.D., Shaw, R.F., Osborne, J.L., Soroka, J. *et al.* (2019). Meta-analysis reveals that pollinator functional diversity and abundance enhance crop pollination and yield. *Nat Commun*, 10.

Zattara, E.E. & Aizen, M.A. (2021). Worldwide occurrence records suggest a global decline in bee species richness. *One Earth*, 4, 114-123.
