# Supplementary material for: Pollinators support the nutrition and income of vulnerable communities
Source: Nature. 2026 May 6;654(8119):683–8. doi: 10.1038/s41586-026-10421-x (PMC13275292; doi:10.1038/s41586-026-10421-x)
Supplement: Supplementary file 2 — Reporting Summary [file 41586_2026_10421_MOESM2_ESM.pdf]

nature portfolio

Corresponding author(s): Thomas Timberlake, Jane Memmott

Last updated by author(s): Mar 9, 2026

Reporting Summary

Nature Portfolio wishes to improve the reproducibility of the work that we publish. This form provides structure for consistency and transparency in reporting. For further information on Nature Portfolio policies, see our Editorial Policies and the Editorial Policy Checklist.

Statistics

For all statistical analyses, confirm that the following items are present in the figure legend, table legend, main text, or Methods section.

n/a	Confirmed	
☐	☒	The exact sample size (*n*) for each experimental group/condition, given as a discrete number and unit of measurement
☐	☒	A statement on whether measurements were taken from distinct samples or whether the same sample was measured repeatedly
☐	☒	The statistical test(s) used AND whether they are one- or two-sided<br>*Only common tests should be described solely by name; describe more complex techniques in the Methods section.*
☐	☒	A description of all covariates tested
☐	☒	A description of any assumptions or corrections, such as tests of normality and adjustment for multiple comparisons
☐	☒	A full description of the statistical parameters including central tendency (e.g. means) or other basic estimates (e.g. regression coefficient) AND variation (e.g. standard deviation) or associated estimates of uncertainty (e.g. confidence intervals)
☐	☒	For null hypothesis testing, the test statistic (e.g. *F*, *t*, *r*) with confidence intervals, effect sizes, degrees of freedom and *P* value noted<br>*Give P values as exact values whenever suitable.*
☒	☐	For Bayesian analysis, information on the choice of priors and Markov chain Monte Carlo settings
☒	☐	For hierarchical and complex designs, identification of the appropriate level for tests and full reporting of outcomes
☐	☒	Estimates of effect sizes (e.g. Cohen's *d*, Pearson's *r*), indicating how they were calculated

Our web collection on statistics for biologists contains articles on many of the points above.

Software and code

Policy information about availability of computer code

Data collection	Data was collected using a customised data collection form using the cloud-based data collection platform CommCare (Version 2.49, http://www.commcarehq.org/ home/) on an Android tablet. All data collection had range checks and internal validity checks built in to help maintain quality control.
Data analysis	Data cleaning and processing were conducted in Stata v18, while all data analyses, modelling, simulations, network analyses, and figure generation were performed in RStudio v2023.12.1 using R (R Core Team 2023). All R scripts used for data processing, statistical analyses, simulations, network analyses, and figure generation in this study, together with documentation and source data required to reproduce the results, are publicly available on Zenodo at: https://doi.org/10.5281/zenodo.18838606<br>Analyses relied on several R packages for data handling, modelling, and visualisation, including tidyverse, dplyr, tidyr, reshape2, readxl, data.table, openxlsx, skimr, ggplot2, cowplot, gridExtra, ggridges, networkD3, webshot2, bipartite, vegan, lme4, emmeans, car, robustbase, broom, scales, and viridis.

For manuscripts utilizing custom algorithms or software that are central to the research but not yet described in published literature, software must be made available to editors and reviewers. We strongly encourage code deposition in a community repository (e.g. GitHub). See the Nature Portfolio guidelines for submitting code & software for further information.

Data

Policy information about availability of data

All manuscripts must include a data availability statement. This statement should provide the following information, where applicable:

- Accession codes, unique identifiers, or web links for publicly available datasets
- A description of any restrictions on data availability
- For clinical datasets or third party data, please ensure that the statement adheres to our policy

The datasets generated and analysed in this study are publicly available in the NERC Environmental Information Data Centre (EIDC) repository at https://doi.org/10.5285/d7434d83-c30d-4186-aab0-9764821cd807 . This repository contains the processed datasets supporting the analyses and figures presented in this study, together with associated metadata. Plant identification during field data collection was supported using the custom-made Plant Atlas for Jumla District, which is publicly available at https://herdint.com/resources/jumla-plant-atlas/

Research involving human participants, their data, or biological material

Policy information about studies with human participants or human data. See also policy information about sex, gender (identity/presentation), and sexual orientation and race, ethnicity and racism.

Reporting on sex and gender	Our study was designed to explicitly take account of differences in nutritional status between sexes. We ensured that our sample size was sufficient to analyse data from male and female participants separately and investigate differences between their nutritional status. Sex was determined based on self-reported information provided by the participants themselves. All data and study results are disaggregated by sex, where relevant, and for any results presented at a population-level in the main text, corresponding sex-specific results are provided in the Supporting Information.
Reporting on race, ethnicity, or other socially relevant groupings	Our study was designed to explicitly take account of differences in nutritional status between age categories (as well as sex). From each of the participating study households, we surveyed the following eligible people: 1) adult woman of child-bearing age (20-48 years at enrolment, 21 to 49 by the end of the study), 2) adult male of 20-48 years (the husband of the adult woman, or the household head if they are not available), 3) unmarried or married adolescent girl (10-18 years at enrolment, 11 to 19 years by study end) 4) young child (6-47 months at enrolment, 18 to 59 months at end of the data collection). We selected these population subgroups as groups 1, 3 and 4 are the most vulnerable to micronutrient deficiency whilst group 2 enabled us to investigate differences between male and female adults. For each participant, we also collected information on their religion and social caste; this information is available in the anonymised source data.
Population characteristics	From each of the participating study households, we surveyed the following eligible people: 1) adult woman of child-bearing age (20-48 years at enrolment, 21 to 49 by the end of the study), 2) adult male of 20-48 years (the husband of the adult woman, or the household head if they are not available), 3) unmarried or married adolescent girl (10-18 years at enrolment, 11 to 19 years by study end) 4) young child (6-47 months at enrolment, 18 to 59 months at end of the data collection). We selected these population subgroups as groups 1, 3 and 4 are the most vulnerable to micronutrient deficiency whilst group 2 enabled us to investigate differences between male and female adults. Individuals within a household were excluded from participating in the study if they: a) Had some disability which prevented them from responding clearly to the dietary recall survey or from understanding the questions. b) Had an ongoing medical condition which caused them to change or restrict their consumption of certain foods. This included diabetes (type 1 and 2), gout, cancer, or any liver or kidney disorder. c) Are only a temporary resident of the village (spend more than two months of the year outside the village, for example on migrant labour). Our final study population consisted of 776 individuals (215 adult women, 186 adult men, 190 adolescent girls, and 185 children under-5 years).
Recruitment	Our ten study villages were selected using satellite imagery and discussion with a local key informant who has worked for many years in the study region. Selection criteria were that the villages should be: accessible (within one day's walking distance from the main population centre); medium sized villages (30–200 households); located in the mid-altitude zone between 2300 and 2800 meters of Jumla district where fruit and vegetable production is common; and broadly representative of the wider district in terms of caste, ethnicity, livelihoods and farming practices. These criteria were designed to ensure that the study villages reflected the typical agricultural and socioeconomic characteristics of the region while remaining logistically feasible for repeated field visits over a 12-month study period. Although restricting village selection to accessible communities may exclude the most remote settlements, the selected villages fall within the dominant agricultural zone of the district and are therefore expected to be broadly representative of smallholder communities in this production system.Within each study village, we randomly selected 20 study households from a pool of eligible households determined through a full census of households in each village. Each of these households was approached by local data collectors who explained the study and asked whether the household would be willing to participate. Fully informed consent was obtained in all cases, and we did not encounter any eligible households declining participation. As a result, self-selection bias arising from differential willingness to participate is unlikely to have materially influenced the study sample.From each household, we aimed to enrol one adult female, one adult male, one adolescent girl, and one child under the age of five as participants in the study. If more than one individual from within a subgroup was present in the household, a random selection process was used to determine which individual was surveyed. If a study household contained only three of the above subgroups, an additional participant (where possible) was sampled from another study household in the same village with additional members within that subgroup to maintain a balanced sampling design. Households lacking these demographic groups were rare in the study communities and therefore very few households were excluded on these grounds, meaning that any bias arising from these eligibility criteria is likely minimal.For all participants over 18 years of age, informed consent (signature or thumb print) was provided; for all participants under 18 years, consent was provided by their parent or guardian and adolescent girls also provided assent.
Ethics oversight	Ethical approval for this study was obtained from the Ethical Review Board (ERB) of the Nepal Health Research Council

Ethics oversight	(NHRC) [Ref: 1709] and the Faculties of Life Sciences and Science Research Ethics Committee (FREC) at the University of Bristol [Ref: 102982]. All procedures involving human participants were conducted in accordance with the relevant institutional and national ethical guidelines.

Note that full information on the approval of the study protocol must also be provided in the manuscript.

Field-specific reporting

Please select the one below that is the best fit for your research. If you are not sure, read the appropriate sections before making your selection.

☒ Life sciences ☐ Behavioural & social sciences ☐ Ecological, evolutionary & environmental sciences

For a reference copy of the document with all sections, see nature.com/documents/nr-reporting-summary-flat.pdf

Life sciences study design

All studies must disclose on these points even when the disclosure is negative.

Sample size	Our final study population consisted of 776 individuals (215 adult women, 186 adult men, 190 adolescent girls, and 185 children under-5 years). When designing our study and and estimating the required sample size, we used data from the Low Birth Weight South Asia Trial for intakes of pregnant women, non-pregnant women and adult men for vitamin A and folate and calculated the detectable difference between two population groups of 200 each, depending on different means, SDs and intra-cluster correlation coefficients when we had 10 clusters with 20 cases in each cluster. If individuals are randomized to the study, then vitamin A detectable difference ranges from 47.63 to 48.19 micrograms and folate from 33.62 to 91.89 between two groups. If clusters are randomized the detectable difference, taking into account intra-cluster correlation coefficients is bigger and amounts to 88.33 to 101.13 micrograms Vitamin A and 73.05 to 162.23 micrograms of folate. Hence we estimated that 200 participants in each population subgroup would be sufficient to enable us to compare intakes of population subgroups and to compare intakes within those subgroups by type of food (insect-pollinated or not), or by broad season of the year.<br>Plant–pollinator interaction data were collected using a standardised sampling regime designed to capture both spatial and temporal variation in interactions within each village. Surveys were conducted fortnightly throughout the flowering season, which corresponds to the approximate timescale over which flowering phenology and pollinator activity change in this system and therefore minimises the risk of missing short flowering periods or brief peaks in pollinator activity. Within each village, we established nine fixed survey plots (60 × 60 m) distributed evenly across the three dominant habitat types (village areas, crop fields, and semi-natural vegetation), providing three replicate plots per habitat type and ensuring representative coverage of the local landscape. Within each plot, 40-minute timed surveys were conducted to record all plant–insect interactions observed during the sampling period. This survey duration provided sufficient time to capture the dominant interactions occurring within each plot while allowing consistent sampling effort across habitats and villages. This standardised sampling design generated 10,975 recorded plant–insect interactions across the study sites, producing plant–pollinator networks that are larger than many comparable field-based ecological network studies. Although interaction counts were not predetermined through formal power calculations, the sampling regime was designed to provide sufficient spatial replication, temporal coverage, and interaction sampling to allow reliable estimation of network structure and species-level metrics. The resulting dataset therefore provides a robust basis for calculating network roles and conducting the simulation analyses presented in this study.
Data exclusions	No observations were excluded from the analyses except for dietary recall records collected before the official start of dietary data collection on 18 November 2021, which were removed prior to analysis. Data quality was monitored throughout the study using built-in range and consistency checks within the CommCare data collection platform and through regular field supervision. Data collectors maintained logbooks documenting any suspected entry errors or omissions. Across the study datasets, 103 dietary survey entries and 104 anthropometric survey entries required correction or clarification. These corrections primarily involved minor data entry issues such as participant identifiers, survey dates, portion size quantities, measurement units, duplicate records, or completion of missing categorical fields. All corrections were verified against field notes and implemented through reproducible R scripts and STATA do-files by the project data management team.<br><br>For the ecological interaction dataset, plant–insect interaction records lacking a valid insect taxon identification or plant species identification were removed prior to network analysis, as these records could not be assigned to nodes within the plant–pollinator network. No additional data were excluded from the analyses.
Replication	Our study was replicated across ten study villages. Results were highly comparable across all sites providing confidence of their reproducibility.
Randomization	Within each of our study villages, we randomly selected 20 study households from within a pool of eligible households. Within each household we randomly selected one individual from within each of our study subgroups (adult female, adult male, adolescent girl, child under the age of five). Randomization was performed in Microsoft Excel using the RANDBETWEEN function.
Blinding	Blinding was not relevant in our study as we were not comparing treatments between experimental groups, we were just recording descriptive data.

Reporting for specific materials, systems and methods

We require information from authors about some types of materials, experimental systems and methods used in many studies. Here, indicate whether each material, system or method listed is relevant to your study. If you are not sure if a list item applies to your research, read the appropriate section before selecting a response.

Materials & experimental systems

n/a	Involved in the study
☒	☐ Antibodies
☒	☐ Eukaryotic cell lines
☒	☐ Palaeontology and archaeology
☐	☒ Animals and other organisms
☒	☐ Clinical data
☒	☐ Dual use research of concern
☒	☐ Plants

Methods

n/a	Involved in the study
☒	☐ ChIP-seq
☒	☐ Flow cytometry
☒	☐ MRI-based neuroimaging

Animals and other research organisms

Policy information about studies involving animals; ARRIVE guidelines recommended for reporting animal research, and Sex and Gender in Research

Laboratory animals

The study did not involve laboratory animals

Wild animals

The study involved wild insect pollinators visiting flowering plants. A total of 10,975 insect specimens representing 503 taxa from 66 families were collected, predominantly from the families Apidae (40%), Syrphidae (21%), Lycaenidae (7%), Calliphoridae (5%), Andrenidae (4%) and Halictidae (4%). Of the 10,975 specimens collected, 5,871 (54%) were identified to species level (76 species), 3,827 (35%) to genus level (grouped into morphospecies), 339 (3%) to family level, and 569 (5%) to order level. Because insects were collected opportunistically during flower visitation surveys, the sex and age of individuals were not determined during field sampling, although both sexes were represented among the collected specimens.

Insects were captured using handheld insect nets while visiting flowers and transferred to collection tubes. Specimens were subsequently euthanised using ethyl acetate in killing tubes, a standard entomological method that preserves morphological characters required for accurate taxonomic identification. Killing specimens was necessary to enable reliable identification to species or morphospecies level, which cannot generally be achieved in the field for many insect taxa.

Following collection, specimens were pinned or placed in paper envelopes (for Lepidoptera), labelled, and preserved as voucher specimens. All specimens were identified by specialist insect taxonomists (see Acknowledgements), and the voucher specimens are stored in the Central Department of Zoology, Tribhuvan University, Nepal for long-term reference. Sampling permission was provided by the Nepal Ministry of Forest and Environment [Ref: 258].

Reporting on sex

Sex was not considered relevant in the case of insect pollinators

Field-collected samples

Insects were captured using handheld insect nets while visiting flowers and transferred to collection tubes. Specimens were subsequently euthanised using ethyl acetate in killing tubes, a standard entomological method that preserves morphological characters required for accurate taxonomic identification. Killing specimens was necessary to enable reliable identification to species or morphospecies level, which cannot generally be achieved in the field for many insect taxa.

Ethics oversight

Sampling permission was provided by the Nepal Ministry of Forest and Environment [Ref: 258]

Note that full information on the approval of the study protocol must also be provided in the manuscript.

Plants

Seed stocks

No seed stocks were used in this study

Novel plant genotypes

No novel plant genotypes were involved in this study

Authentication

Authentication was not relevant or required in this study
