# Supplementary material for: Pollinators support the nutrition and income of vulnerable communities
Source: Nature. 2026 May 6;654(8119):683–8. doi: 10.1038/s41586-026-10421-x (PMC13275292; doi:10.1038/s41586-026-10421-x)
Supplement: Supplementary file 3 — Peer Review file [file 41586_2026_10421_MOESM3_ESM.pdf]

nature portfolio

Peer Review File

Pollinators support the nutrition and income of vulnerable communities

Corresponding Author: Dr Thomas Timberlake

Any redactions in this file are there to maintain patient confidentiality, the confidentiality of unpublished data, or to remove third-party material.

This file contains all reviewer reports in order by version, followed by all author rebuttals in order by version.

Version 0:

Reviewer comments:

Referee #1

(Remarks to the Author)
It has been a pleasure to review this highly ambitious paper by Timberlake et al., which attempts to link the 'health' of pollination services to the health of people by exploring the link between the pollinators in a landscape context, crop pollination in smallholder farms, and human nutrition of the subsistence farming community. The authors use empirical ecological data on plant (crops and wild plants)-pollinator interactions and crop pollinator dependency and nutrient content, and dietary surveys of subsistence farmers to create a – primarily descriptive – case study that goes beyond a proof of concept by delivering valuable insights into the importance of pollinators in underpinning human nutrition. Further, the authors use simulations to explore the impact of reduced (full collapse of pollination services or continued decline of pollination till 2030 as a result of the current rate of insect decline) or increased pollination on nutritional and financial security of the subsistence farmers. The correlational narrative of the study combined with the simulations that aim to predict the consequences of pollination decline for human health generated an impressive piece of work that sheds light on the how and to what degree human health is dependent on fully functional ecosystem services.

The research is entering novel ground in the level of detail at which the connections between pollination and human health are described, the temporal and spatial resolution, and the choice of the study system, which convincingly argues for a high level of generality of the findings. I found the interpretation of the findings, the critical reflection on uncertainty and the overall robustness of the conclusions convincing, partly because of a detailed justification of the study and it's underlying assumptions, and the associated sensitivity analysis.

In addition to the few minor comments below, I have only one main critique. I feel that the study falls short of its analytical potential to identify quantitative differences in the pollinator community and associated changes in crop pollination and nutritional value. The authors collected plant-pollinator communities, and, for example, by employing a network approach (either bipartite between plants and pollinators, or multi-level across all 'interaction' types) the study could provide a more significant contribution towards the generalisability of the findings. More specifically, if differences in the structure of the interaction networks could be compared to patterns of nutritional benefits (or detriments) it would provide the wider community working with plant-pollinator communities in agro-ecosystems with a tool to draw conclusions between the structure of the ecological community and potential consequences for human health. I have the impression that not exploring these connections more systematically with, for example, a network approach (but other quantitative analytical approaches may equally work) is a missed opportunity that render this study less 'useful'. Is there scope for the authors to address this opportunity as part of this work?

Here are a few minor point that may require further clarifications.
L89. I feel that the term 'changes in pollination service…' is a little vague and would perhaps benefit from a rewrite to be more specific.

L165. Delete 'in'

L358. This statement may require strengthening with some reference or data. While I don't question the general notion that

year to year diet is reasonably similar, I'm wondering whether some information of interannual crop rotation for smallholder farms in this region would be available to support this claim.

L384ff (and/or Appendix S7). It's not entirely clear whether pollen on insects were identified in any way and/or whether only pollen grains of the last visited plant species of the specimen were counted. Given that heterospecific pollen loads may be high and conspecific loads may be low in some species (e.g. generalist pollinators with low floral fidelity), some distinction about the type of pollen may be needed here. Please clarify in the text, perhaps just by emphasising that this measure of pollinator importance is not specific to plant species.

(Remarks on code availability)

Referee #2

(Remarks to the Author)
This is an impressive paper linking pollinator services to human income and nutrition. In general, the paper is very well-written, and all claims are backed up properly. I especially appreciate the detailed repository on GitHub, making all results reproducible. Adding a DOI (e.g. via Zenodo) to the repository would make it citable and permanent.

Given the complexity of this very well crafted paper, I have some minor comments aimed at clarifying some decisions taken, or asking to add some caveats in the discussion. I include those as "in-line comments" below. The only major caveat I have when reading the paper is the comparison with the different pollinator trend scenarios. I understand that the "no pollinators" scenario is a straightforward comparison to make, but it is a scenario that can be not too informative. Despite the Chinese example cited (which is a more complex story than just losing pollinators), losing pollinators completely is an implausible scenario, especially in a heterogeneous landscape such as the one studied. In fact, the "pollinator reduction" scenario is also quite simplistic, and assumes a declining rate equal for all pollinators based on the recent decline in honey harvesting. This decline might be a good proxy (and I like how it is justified), but it is also a strong, untested assumption, especially as many species in the network are solitary. Finally, the "enhanced pollination" scenario also relies on complex assumptions (see specific comments below on that). I want to stress that all three scenarios make sense and are correct, and hence, if the authors want to keep them (while adding a few caveats to the text; see in-line comments), I am fine with this decision. However, I would prefer to focus on a continuous "pollinator rate of change" scenario and test different levels of increases/reduction (e.g., from +50% to -50%, which is justified by the literature). Ideally, this can include some response diversity across pollinators (e.g., the decline rate for each species is drawn from a normal distribution with some variability). As predicting pollinator changes in the next years is a daunting task, providing the numbers for a range of situations could be more informative. Again, I want to stress that this is only how I would do things, in case the authors find it interesting, but the 3 scenarios presented are valid and justifiable.

in-line comments:

Line 65: It is unclear to me from the citations if pollinator decline risk is higher in areas with small-holders. Maybe you can explain why (e.g., in comparison with areas that suffer from agricultural intensification) or tone down this claim.

Line 80-83: In the main text, you do not mention that the Jumla pollinator trends mentioned are based on honey harvest statistics. I would use this info with caution, as honey production per hive does not imply fewer hives, nor is it extrapolable to other social and nonsocial species. Again, I would tone down what we know about pollinator declines in the area.

Line 105: This is extensively answered below, but I was wondering here if these crops are locally consumed, and a hint here might help the reader.

Figure 3: I wonder if it's worth combining these two plots. I spent some time linking both mentally, and if I understand correctly, the orange bars in a) can be integrated into the green bars in b). Just a suggestion if you think it enhances the visualization.

Line 157: Again, you don't know pollinator declines in your area, you can keep the reference, but explain the details a bit more to avoid misleading claims.

Line 166: Given the severe under-nutrition in the area, I would appreciate a quick statement on what other factors (other than pollinator) are at play. Even when you show that pollinators can alleviate the situation, even the best pollinator scenario shows nutritional problems for the population. This can be highlighted in conclusions, for example.

Line 171-173: Until reading the methods, how "decline" is extrapolated, and what "optimal" means, is unclear. Here is where a continuous scale of pollinator changes would help me understand this directly.

Line 187: "persists even when consuming below…". This part is confusing to me; maybe changing the wording can help (or maybe it's only me).

Line 205: Here, the % of "no pollinators" and "declining pollinators" are hard to compare as you need to move between paragraphs. I understand that from 44% for no pollinators it goes to 14% for declining pollinators, and for nutrients it drops

from ~22% to ~6%. I find more relvant the second scenario, and would focus on this one.

Line 213: Here, one wonders what else is limiting crop production. Temperature, fertilizers, pests? Is pollen limitation a relevant issue in comparison with these other drivers?

Figure 4: I would not reuse colors from Figure 3 with a different meaning. I like how the gradient is presented here, in line with my main comment above. In contrast, Figure 5 capitalizes only in the no-pollinators scenario.

Looking at the figure, I wondered why adolescent men are not targeted. I wonder if the assumption is that they equal adult men regarding diets; if so, this can be stated in the methods. It is just a curiosity.

Line 248-241: Here, I would love to see if wild plants are visited before and after the crop (indicating clearly they support pollinators complementary to crops) or simultaneously to crops (where competition might (or might not) be occurring).

Line 257: A piece that is missing is which is the plant contribution to pollinator fitness. This is (almost) impossible to get, so it is ok, but can be stated as a caveat. Here, the assumption is that it is proportional to visitation rates.

Line 160: I would be cautious in recommending increasing beekeeping, as it can cause collateral problems. In addition, what about the effects of weeds (which support pollinators) competing with crops, or sacrificing space needed to grow crops to plant ornamental plants? I think this sentence can be more balanced, showing pros and cons.

Line 264: Flower strips work for conservation, but do not always have economic benefits (e.g., work from David Kleijn group).

Line 339-340: But pollinators are also expected to enhance fruit quality (e.g., micronutrients), so your estimates from the literature are conservative.

Line 343: I am impressed with the amount of careful information gathered. Were the participants remunerated? I am just curious about this (and if they were would be good to state this).

Line 379: By “visiting a flower,” you refer to making contact with the plant’s reproductive parts? If so, this would indicate potential pollination with a higher degree of confidence.

Line 465: What about crops such as carrots? (which are pollinator-dependent for seeds only).

Line 472: As stated above, honey per hive does not necessarily relate to number of individuals, and other environmental variables might explain this. In addition, social dynamics are not comparable to solitary lives. In addition, while the linear assumption between visitation and production is straightforward, it is unlikely to be the best functional form, as yield should saturate with visitation (also line 488). Maybe this can be mentioned.

Line 485: But on the other side, you assume a common response across pollinators, while it is well known that there is response diversity across pollinators. In fact, your network approach is perfectly suited to explore the role of response diversity in buffering the effects (e.g. by testing declines with/without response diversity), but I understand if this is beyond the scope of the paper.

Line 498: This part needs details. Yield gap can be caused by many factors, and how do you decide which fraction is attributed to pollinators needs explanation. In supplementary material is also unclear (see below).

Line 505: Here is “mean yield”, right? I would make it explicit.

Line 532: Version of R is more relevant than that of R Studio (which is just a graphical interface for R).

Supplementary Text S8. Pollinator exclusion experiments: I am surprised that you have data on hand pollination too (which is not used). I wonder why not using this data to calculate “optimal pollination”. This would simplify your assumptions, as the difference between hand and open pollination is clearly attributable to pollen limitation.

Supplementary Text S10. Predicting local pollinator declines and associated crop yield
declines: The R2 of the model used to extrapolate is needed, as predictions should be made with models that have a decent fit only.

Supplementary Text S11. Predicting yield increases from enhanced pollinator
abundance: Here, I understand that you assume 24% of the yield gap is attributable to pollinators. This would need a bit more justification. It can also be reflected better in the main text.

Best,
Ignasi Bartomeus

(Remarks on code availability)
I just skimmed the code and documentation (which is super complete!), but did not ran it locally.

Referee #3

(Remarks to the Author)

The manuscript aims at connecting pollinator services and nutrient intake, based on thorough primary longitudinal data collection of individuals' dietary intake and flower visitation by pollinators. The longitudinal feature was used for descriptive purposes (seasonality), and associations were based on indicators averaged across the full data collection period.
The introduction (and overall paper) is very well written and clearly introduces the reader to the topic, even non-experts in pollination like me.
In response to question 1, the paper shows how pollination deficit and adequacy of the diet are connected and quantifies the contribution of pollinators to dietary intakes. The strengths of the paper include primary data collection for four different populations with quantitative dietary assessment every month for a year, with indication whether each food was locally obtained. This allowed to quantify micronutrient intake coming directly from pollinator-dependent local or imported foods. Using a thorough survey of plant visitation in the same study area a year earlier (lack of overlap was due to COVID19 restrictions for household surveys), the authors also quantified the yield of these foods attributable to a range of local pollinators, allowing them to calculate the contribution of each pollinator to micronutrient intake. Findings from these complex primary data analyzed in an original way deserve to be disseminated, as the authors make a very concrete and convincing connection between pollination and healthiness of the diet.
I am less convinced by the interpretation of the simulations used to respond to questions 2 and 3 because they are based on multiple rather unrealistic assumptions of proportionality (e.g. that intake of any crop would vary proportionally to its production). These assumptions are very transparently acknowledged. Although this seems fine to use these assumptions to quantify which share of the observed income and nutrient intake is attributable to pollinators, the interpretation of these attributable fractions as reduction of nutrient intake or wealth which resulting from changes in pollinators effectiveness is less convincing. The message would be more convincing if it interpreted these results as the share of income, PA or micronutrient intake attributable to pollination in the observed scenario, rather than trying to model a change when the moving variables are unlikely to be independent from each other. Alternatively, if data allow, the modification of these assumptions to rely on more sophisticated elasticities may be more appropriate and more convincing.

In addition to these overall comments, I provide detailed comments on the dietary intake methods, which I was specifically asked to review, and found appropriate overall. Data collection methods were strong. However, the calculation of nutrient intake would need to account for micronutrient retention factors if it did not, or the paper needs to describe that it did. Finally, the calculation of probabilities of nutrient adequacy was appropriate but needs to be described in the paper. More details are provided below.
Three to four individuals of four different populations (young children, adolescent girls, women of reproductive age and adult male) were sampled in each household, and quantitative 24-hour recalls were conducted with each individual every month for a year. All individuals were presented together, and limited population-stratified results were presented. Dietary intake data collection described in supplementary texts S3 and S4 corresponded to standard and was well described. The use of a five-stage multi-pass survey method is appropriate, and the use of standard recipes to capture detailed ingredients of mixed dishes is appropriate as well. Portion sizes were also estimated using standard methods, although it may be useful to clarify how leftovers were handled for portion size estimation. The authors transparently disclosed that the CommCare data collection form was not flexible to amendments, but they put in place a strong correction process based on enumerators amendments logs.
My major concern related to diet data management methods is that the authors did not describe if micronutrient retention factors relevant to the cooking method were accounted for. When food is cooked (e.g. boiled, steamed, stewed, etc), loss of 5-25% of micronutrient content is common, and it can go up to 50% for certain micronutrients and certain foods. Published tables of these retention factors exist in the literature. Loss of nutrients due to cooking may significantly affect the intake of these nutrients. I don't know how the results presented in the manuscripts may be affected by this. I recommend the authors should account for nutrient retention factors and/or clarify that they did.
A second major concern is that the author did not describe at all how they calculated probabilities of dietary adequacy from observed nutrient intakes. The authors need to add a summary of the process in the main methods. I reviewed the R code to get the description and reviewed how this was doneI understood that the mean of the 12 records for each individual was assumed to approximate usual intakes for all nutrients presented in the paper. Due to large within to between-person variability in micronutrient intake, a large number of observation days is needed to adequately approximate usual intake by a simple mean. The exact number of days required for this varies ; the literature cite ranges between one and several dozens of days. The study dataset with a dozen observation days across a year for everyone is unique, and probably good enough to get a reasonably reliable estimate of usual intake with a simple mean. However, the authors may want to briefly justify and discuss this using relevant references. Then, the probability approach used to obtain probabilities of adequacy from usual intake is strong. However, it needs to be reported in the paper and the reference provided. Also, I would like to know which dietary requirements were used and which levels of bioavailability were assumed for zinc and iron. Detailed supplemental text may be deemed useful to describe specificities such as iron adequacy calculation (skewed distribution of requirements was assumed), bioavailability for iron and zinc, and references for nutrient requirements considered (EAR and CV or SD).
Finally, though the title is punchy, I have concerns with it. First, the use of "health" in the title is misleading, it implies that some health indicators of individuals were measured and associated with pollination deficits, which is not the case. I suggest replacing health with "healthiness of the diet". Second, as explained above and below, the limitations due to pollen deficits result were simulated based on strong/simplistic assumptions. The title should either clarify that this is based on simulations, or turn it the other way around, stating that pollination contributes to healthiness of the diet and wealth of impoverished communities.

Specific comments on dietary methods and results:
Main text, line 334. As I don't see any mention of yield factors, I assume the proportional composition of each ingredient was based on raw weight. Please clarify in the sentence "calculated based on raw ingredient weight" (or refer to the use of yield factors if they were used).
Main text, line 340. References for published Food Composition Tables are missing.
Main text, line 342: please describe how probability of adequacy was derived from nutrient intake across multiple days.
Line 402: add reference of published food composition tables.
Lines 419-420: Do you have data or a reference showing the magnitude of the use of own seeds versus imported seeds?
Lines 448-460 and supplementary text S1, assumptions: This study has data that could support assumption 1, or refute this assumption. Study data could be used to show the variation in consumption of a crop depending on variation in yield of this crop in the population. I am not an expert in simulations, but wonder if it would be feasible for authors to use study-specific elasticities of crop intake depending on crop yield, or better, elasticity of micronutrient intake depending on a number of crops yield? This would allow to get rid of strong assumptions 1 and 3, which may not be realistic. My specific expertise does not allow me to assess the plausibility of other assumptions. However, these assumptions would appear more convincing if supported by more references or data. Assumptions of proportionality seem simplistic. Also, an assumption is not presented, the assumption that crop yield declines proportionally to the decline in pollinator*pollinator dependance value. This is explained at the end of supplementary text S10 but should be presented as an assumption.
Figure 2: it is a very nice figure visually. However, it is unclear to me what the size of a crop bar represents. Is this the sum of all contributions to micronutrient intakes for all micronutrients together? This is what it seems to indicate but I am not sure it makes sense. I also wonder which unit are used to figure micronutrients' flows, is this percent of total micronutrient X intake?
Figure 3: it is difficult to assess how much pollinator dependance is local or imported. Would it be useful to combine both panels into 1 figure with 4 categories?
Figure 4: vitamin E is presented everywhere else except on this figure.
Figure 5: Y-axis graduations are not harmonized. Are the differences significant and meaningful between original and pollinator loss (see e.g. Calcium and vitamin A)? In line with my comment right above, this figure shows the PA attributable to pollinators (Original PA minus PA with no pollinators).
Supplementary text S3, section Dietary recall survey, first paragraph. Clarify who responded to the dietary survey for adolescents aged 11 and 12 years. If a caregiver responded, were the adolescents also present? Also, how often did the cook respond on behalf of an absent adult? This practice may allow to capture foods consumed at home during meals but is unlikely to correctly capture foods consumed as snack or away from home, so it must be shown that it was not frequent.
Supplementary text S3, section Dietary recall survey, third paragraph. The use of number of items is described as a portion size estimation method here but not in the main text. I suggest to add this in the main text as well (lines 320-324). Also, the authors could add a sentence to clarify if the questionnaire handled leftovers. Finally, is consumption from the shared pot common in this population?
Supplementary table S1. Am I correct that the contribution of all insects to each micronutrient intake would be the sum of all insect-specific contributions? If yes, would be useful to present this sum in table S1 to figure the total contribution of pollinators to each micronutrient intake? Also, I suggest using "intake" instead of "supply". Finally, I am not sure I fully agree with the interpretation on what these numbers mean (last sentence of figure caption). To me, it would be more appropriate to conclude from the calculation that 5.12% of folate intake originated from pollination of various crops by A. cerana.

Other specific comments:
Abstract, line 42. Which approach to which ecosystem services? I struggle to understand the sentence.
Lines 90-93: may not be needed, sounds like a repeat of lines 77-83.
line 155: I suggest replacing "nutrition" with "diets". I am not sure that I agree with the use of the word "impact".
Line 197: which health outcomes? Micronutrient adequacy should not be considered a health outcome.
Lines 203-204: which specific interventions are the authros referring to?
Line 302 : clarify list of eligible households was "obtained by census"
Line 305: not sure the word "representative" is appropriate here. I suggest replacing with "diverse".
Line 468: income that would be lost… but the absence of pollinators was not observed. Would it rather be the proportion of income attributable to pollination/pollinators in the observed scenario? As for my other suggestions below, I would not assume about too much about what would happen with change (assumptions seem very strong), but I find it interesting to present the proportion of what is observed which is attributable to pollinators.
Line 520: Genuine question: how does Vip depends on actual presence and concentration of the wild plant on the plot?
Supplementary text S3, section Selection of villages. Clarify in the first sentence that study villages were purposively selected. On 5th line, the word "representative" is confusing in a paragraph on selection. To avoid confusion with representativeness from random selection, I suggest to replace with a synonym such as "typical" or "similar to".
Table S5. Details on Z-score and anthropometric indicators calculation are not explained anywhere. They could be added (with relevant references) to supplementary text S5.

(Remarks on code availability)
I reviewed the code Script_04_Probability_adequacy_calculations.R, which was clearly presented and well commented. It allowed to clarify the methods and commands used to calculate probability of adequacies. I did not try to install or run it.

Version 1:

Reviewer comments:

Referee #1

(Remarks to the Author)
I had the pleasure to read and comment on the previous submission of this manuscript. After carefully reading and considering all changes made to the revised manuscript I'd like to commend the authors to this greatly improved manuscript of an already impressive piece of work. The detail and implementations of the revisions are exemplary, further substantially strengthening the overall fundamental and applied usefulness of the study.

With specific reference to my previous comments I would like to thank the authors for carrying out the network analysis, which revealed the importance of certain keystone pollinators for maintaining/increasing human nutrition in the study region. Identifying their positions in the network provides evidence for link between network structure and function, which is clearly highlighted in the manuscript.

I'd like to thank the authors for make this valuable contribution to the field, which puts a spotlight on the importance of pollinators for human health.

(Remarks on code availability)

Referee #2

(Remarks to the Author)
I want to thank the authors for the detailed revision. All my comments are addressed appropriately, and I think this is a strong paper showing pollinators contribution to people. That said, in response to another reviewer, some extra analysis were added to the ms, and I have some extra questions about those.

Sorry to add noise by thinking differently that other reviewer, but I am skeptical that network structure can be easily linked to total nutrient intake. For example, there are no good hypothesis (in text, or that I can came with) relating specific plant-pollinator network metrics to total nutrient intake. In fact, as expected, the analysis shows no specific associations. I am ok leaving this exploratory analysis in text if the authors think is useful, but some details are needed. i.e.

line 654: This is the network composed of the crop plants and pollinators, right? Wild plants are not included here. I'd make it explicit.
line 657: Make explicit which indexes are used. All this indexes can be calculated in many different ways (with different interpretations!). Most importantly, I could not find these network level results either in tables or figures in the supplementary.

I am more positive on the use of species level metrics, and relating network roles with its contribution to nutrient intake. I think here there are plausible hypothesis to test. However, here the analysis is done are again in a very exploratory way to my taste, and with few hypothesis on why some indexes can explain or not the outcomes. For example:

321: Here I wonder if degree, nestedness rank, and weighted closeness are correlated? If so, it would be good to state this. And more importantly, I wonder if they are correlated with pollinator abundance. If so, it might be an easier measure to take than network indices, and more informative.

662: By removing one species at a time, the effect of abundance might be much more important than the effect of their interaction patterns (which are likely also influenced by abundance). Acknowledging this will be important and maybe would be good to account for abundance, specially if its correlated with both, network indices and nutrient uptake.
668: Univariate analysis of 20 variables seems a bit of a fishing expedition to me. It's hard to change this now, as knowing the results, the choice of a narrower set of indices will be biased. In any case, I'd suggest showing the correlation among those indices, as many will reflect similar situations (i.e. be highly correlated). One can argue that the same species that have a high degree (and probably abundance) are also central and contribute to Nestedness, so you can not disentangle the effects. This is ok, but should be explicitly stated (if this is the case as I suspect).
671: Maybe I am confused here. Did you perform 20 models (one per variable) using all species across all villages? If so, the village should be in the random structure, right?
Table S8 clarifies that you used a single predictor per model… in this case, R2 and AIC tell you the same information, and I would use only R2 for clarity. I still think many indices will be correlated and a clearer picture can be obtained to answer this question by showing a more in depth analysis.

Finally, two small cosmetic edits:
Line 204 and 601: I would use instead of "a worst-case outcome" something more neutral such as "an extreme scenario"
Line 264: Giving the lack of strong associations I would change "but also reveals" to "but also potentially reveals".

I re-state that the rest of the paper has gained in quality and should be published, once this new analysis is clarified.

Best,

(Remarks on code availability)
I skimmed the scripts, but did not do a full detailed review.

Referee #3

(Remarks to the Author)
I have reviewed the authors' responses to my comments and questions, as well as the related changes in the manuscript. I would like to commend the authors for their revisions which make the manuscript stronger. Overall, the manuscript is clear, well written, and provides evidence for a clear mechanistic link between pollinators and people's micronutrient adequacy and wealth, through pollinator-dependent crops yield and intake.
The re-framing of the interpretation toward the contribution of pollination to diet and wealth, with the various scenarios presented as "boundaries", addressed well my concern while supporting as strongly as previously the same take-away findings (if not more strongly). I like the new title and I am fine with keeping the word "health", now associated to "support" in this new title. Indeed, I agree that "supporting health" is an appropriate attribute for micronutrient adequacy. I also appreciated the additional text (S1) and presentation of additional data to support the assumptions (e.g. figure S7, table S10). The authors also added the appropriate level of detail on diet data management and analysis in the main manuscript and supplementary file and provided references where relevant.
Nevertheless, the revisions brought two additional comments for the reviewers.

First, the revisions brought one question related to the first simulation (scenario 1). The authors state on line 185, 204, 538 and 601 that they model complete loss of *local* pollinators. However, most descriptions in methods and results do not mention "local" and refer to "complete pollinator loss" or "pollinator-dependent share of each crop's yield". Could the author clarify if the relative proportional decline in yields was assumed only for local pollinator-dependent crops or if this was also applied to the imported pollinator-dependent crops? I am asking because the effect on PA of vitamin E presented on Figure 5 appears rather large, in spite of a small proportion of vitamin E intake provided by local pollinator-dependent crops as shown in new Fig 3. On line 201, the reported decline for vitamin E intake is 18%, which is consistent with Figure 4 (which shows impacts of pollinator loss), but inconsistent with table S7 also referred to (which shows a 3.9% decline associated with complete *local* pollinator loss). In table S8 of the previous version of the manuscript, it was clear that considering replacement of local crops only (i.e. due to complete "local" pollinators loss) resulted in very different results for vitamin E compared to replacement of local & imported crops (i.e. due to complete pollinators loss both locally and for imported foods). This was less true for other micronutrients. The current version of the manuscript is confusing, because it has kept only one of these two options for the new analysis (resulting in Figure S11), but it is unclear which one, and pieces of both of these options seem to be reported throughout methods and results.
Other specific points related to scenario 1:
on Figure 4, please clarify what indicators are plotted for micronutrients. From the text, I assume it is intake and not PA. It should also be clarified in the figure or figure caption.
Lines 233-234: Where is the "additional 18% of the population at-risk of deficiency in at least one micronutrient" shown on graph 5? It shows additional population at-risk for each micronutrient, however, it is unclear where the 18% was found there.
Line 280: same question for "9% of the population into sufficiency in at least one micronutrient".

My second follow-up comment is about the nutrient requirements used by the authors to assess the probabilities of adequacy, which are now clearly referenced.
FAO/WHO. Vitamin and mineral requirements in human nutrition. (World Health 896 Organization & Food and Agriculture Organization of the United Nations, 2004).
I recognize that these requirements are the latest published by FAO and WHO, and therefore may be deemed an obvious option for use in countries where there are no published vitamin and mineral requirements. One drawback of this set of requirements is that it is very old and perhaps outdated. In 2020, Allen, Carriquiry and Murphy reviewed the most recent evidence and published a proposed set of harmonized requirements for global use, picking into IOM and EFSA requirements. Some values are rather different from the FAO/WHO values. I thought I would bring this reference to the attention of the authors, who might want to consider using this more recent set of requirements based on more recent evidence.
Reference: Allen LH, Carriquiry AL, Murphy SP. Perspective: Proposed Harmonized Nutrient Reference Values for Populations. Adv Nutr. 2020 May 1;11(3):469-483. doi: 10.1093/advances/nmz096. PMID: 31701998; PMCID: PMC7231601.
Another minor point on requirements: in supplemental text S7, under "Nutrient requirements", it would be useful to add which sources were used for vitamin A and vitamin E requirements.

Besides the changes related to my comments, I reviewed the full manuscript, including the changes resulting from other reviewers' comments. I agree these changes improved the manuscript, and I do not have further comments or suggestions on these other changes.

(Remarks on code availability)

Version 2:

Reviewer comments:

Referee #1

(Remarks to the Author)
I have been asked to comment specifically on the revised network analysis after receiving further comments from Reviewer 2 on this previously added set of analyses. While I generally agree with the reviewer's comments of grounding the network analysis in a hypothetical framework that clearly outlines the expected outcomes, I have doubts whether the revision satisfactorily addresses these concerns. I'll attempt to explain my rationale below:

The authors argue that to minimise the exploratory nature of their network analysis they remove all network-level analysis from the revised paper. While I sympathise with the authors' approach, I'd like to emphasise that there is nevertheless a strong basis for using network-level metrics to support a hypothesis-driven exploration of the plant-pollinator interaction data (as done in many influential networks studies to date - but see for a more critical reflection: Dormann 2023 The rise, and possible fall, of network ecology. In: Defining Agroecology. – A Festschrift for Teja Tscharntke. Eds.: Dormann, C.F., Batáry, P., Grass, I., Klein, A.-M.,Loos, J., Scherber, C., Steffan-Dewenter, I. & Wanger, T.C. Tredition, Hamburg, pages 143-159). As the network-level analysis (using the selected metrics) did not reveal any obvious patterns, however, I agree with the argument that a more extensive analytical framework would be beyond the scope of the paper and the (other) key messages conveyed therein.

The argument of using species-level metrics to refine and direct the analytical approach is understandable and transparent. What I have difficulties with, however, is the choice of metrics, the paper(s) that support the metric selection, and the analytical approach. Equally, I find the formulation of the hypotheses relatively week and unsupported (Line 721-723), lacking detail and references. This lack of clarity translates in the choice of species-level metrics. It is well known that abundance (and to some degree species richness) of pollinators and flowers can explain a large proportion of network patterns (see Blüthgen & Staab 2024 AREES for a recent review). The study cited by the authors to support their rationale of choice of species-level metrics (Coux et al. 2016) uses normalised degree specifically to account for network size when comparing degree between networks. It's not clear whether the authors used normalised degree (or the fully quantitative measure of proportional generality) here - please clarify. Degree in itself is also a unweighted metric, which does not incorporate some of the information benefits of weighted metrics, and comes with a risk of misinterpretation as a result of its relatively weak interpretation strength (see again Blüthgen & Staab 2024 for a more detailed reflection and references on weighted vs unweighted metrics). I can see the authors' attempt to separate unweighted (degree) from weighted (interaction strength) metrics (see also Dormann 2020 bipartite manual), but as shown in Fig. S15, they are both highly correlated with abundance. While abundance is a critical and intrinsic ecological condition of communities, network analysis can go beyond abundance patterns and reveal functional consequences (as attempted here), e.g., whether a more generalised network contributes to a higher functional performance or community stability (the authors allude to the latter but do not actually test this with their analysis).

There are, next to d', other species-level metrics that shed light on the structural roles of species, such as centrality metrics, and z-scores or null models can be used to enable comparisons across networks standardised by sampling effect or variation in abundance. Finally, beta-diversity metrics that reveal the importance of species turn-over or rewiring can provide important context on the stability of the nutritional patterns associated to plant-pollinator interactions across communities.

I recognise that these aspects were not explored in the first set of revisions, but it was my understanding that a more comprehensive (across network-level and species-level) exploratory analysis was an appropriate step towards showing the potential of the data for further in-depth network analysis by future studies. After stripping down the analysis to three species-level metrics, the message has changed somewhat, and the shortcomings listed above have become more obvious.

To present the benefits of a hypothesis-driven network analysis I'd suggest that the authors keep the focus on species-level metrics but strengthen the justification and hypotheses for each individual metric used, and support the rationale of their arguments (e.g., role of individual species, stability of patterns and their impact on nutrition) with appropriate analysis (e.g. testing against null models or use z-scores to compare variation in abundance and species richness across networks). Which approach to select will depend on (the ecological relevance of) differences in sampling intensities across networks.

Finally, I'd like a clarify the authors' choice to log-transform d'. As theoretically d' can contain values of zero, a log+1 transformation is advisable. If the authors transform with log+1, there is very little/no differences between d' and (log+1)d'. Can the authors please provide a justification of the transformation to support the choice of analysis.

(Remarks on code availability)

Referee #2

(Remarks to the Author)
Thanks for revisiting the network analysis. I am very happy with the current implementation, and I think that despite the less shiny results regarding network importance, the result showing abundance is driving most of the responses is very relevant.

I have nothing else to add. Congratulations for this nice piece of work.

(Remarks on code availability)
I did not checked the code this time.

Referee #3

(Remarks to the Author)
I reviewed the third version of the manuscript “Pollinators support the health and wealth of vulnerable communities”. I confirm that my concerns were adequately addressed.
I appreciate the clarified focus on locally produced pollinator-dependent crops, with a sensitivity analysis showing the effect of including non-local crops into the scenarios.
I understand that the authors preferred not to switch vitamin and minerals requirements. Indeed, calculating probabilities of adequacy with the Allen and al. harmonized requirements would imply going back to the detailed documentation of each of the EFSA and IOM selected requirements and extract the coefficients of variation. As the methods are clear on the use of the WHO requirements, I am fine with the author’s decision to not switch requirements.
Overall, the paper makes an important contribution to the field, showing the magnitude of the contribution of pollination and specific pollinators to dietary adequacy and income in an exemplary context, and supports a call to facilitate management of local pollination services. I learned a lot from reviewing this manuscript.
In addition, as requested, I paid particular attention to the statistics and error bars. The variability of PAs is not presented on Figure 5, although adding it would make the figure very difficult to read. One option (to be confirmed if acceptable by the editorial team) might be to clarify in the figure legend that SDs for the current pollination scenario are presented in Table S6. I don’t seem to see anywhere indications on the distribution of PAs estimates with the “no pollination” and “optimum pollination” scenarios, does it need to be added? Where relevant, error bars are appropriately used and what they represent is defined in the corresponding figure legends. It seems to me that the Journal is expecting a full definition of all box-plot elements ; this would not be currently provided in Figure S17. One last point: There seems to be a small mismatch between Figure 3 and Table S6 in the mean for adult women’s PA of vitamin A and calcium (current scenario).

(Remarks on code availability)
I did not review it this time, as I had no issues with the code in the first review round.

Version 3:

Reviewer comments:

Referee #1

(Remarks to the Author)
I would like to thank the authors for thoroughly addressing my previous concerns about the species-level network metrics and controlling for abundance. Also, I very much appreciate the authors' explanation of the rationale for keeping the main focus of the study on agricultural and health benefits of pollinators, and exploring the data in a network context in another study.

This is an excellent study, and I can only commend the authors for their attention to detail during the revision process. Congratulations!

(Remarks on code availability)

Referee #2

(Remarks to the Author)
I (R2 in previous round) read the further details on network analysis suggested, and I thank the authors for thoroughly checking all possibilities. As expected, this more refined analysis do not change the main conclusions, and as I said in my last revision, I support the publication of this manuscript as is. The new analysis are well explained and make the manuscript more robust.

Best,

(Remarks on code availability)
I did not checked the code this time.

Open Access This Peer Review File is licensed under a Creative Commons Attribution 4.0 International License, which permits use, sharing, adaptation, distribution and reproduction in any medium or format, as long as you give appropriate credit to the original author(s) and the source, provide a link to the Creative Commons license, and indicate if changes were made.
In cases where reviewers are anonymous, credit should be given to 'Anonymous Referee' and the source.
The images or other third party material in this Peer Review File are included in the article’s Creative Commons license, unless indicated otherwise in a credit line to the material. If material is not included in the article’s Creative Commons license and your intended use is not permitted by statutory regulation or exceeds the permitted use, you will need to obtain permission directly from the copyright holder.
To view a copy of this license, visit https://creativecommons.org/licenses/by/4.0/

Response to reviewer comments (Manuscript: 2025-03-06493)

We are immensely grateful to the editor and the three reviewers for providing such thoughtful, positive and constructive comments. In response to these comments, we have undertaken substantial new analyses, clarified our methods and assumptions, and expanded our discussion to highlight the broader relevance of our study.

To briefly summarise, we have made five major changes to the manuscript:

1) New network analysis (suggested by Reviewer 1): We conducted a new analysis linking plant–pollinator network structure to nutritional outcomes and show that pollinator network roles are strong predictors of the nutritional impact of their removal. These findings expand the broader relevance of our results by revealing pollinator network roles that can serve as useful indicators of their functional importance to human health. This appears as a new objective in the manuscript (Question 3), with new Fig. S14 and Table S8.

2) Continuous, probabilistic decline framework (suggested by Reviewer 2): We revised our model structure to allow for a continuous, probabilistic simulation of pollinator decline which incorporates response diversity among species. This enables us to visualise the relationship between pollinator abundance and nutrient intake across a wide range of decline scenarios (new Figure S11), providing a more flexible and realistic picture of potential outcomes, as well as a more accurate idea of the uncertainty associated with the predictions. We retain the three original discrete scenarios in the text though, as intuitive reference points, but now refer to them as illustrative boundary estimates, rather than forecasts.

3) New phenological analysis (suggested by Reviewer 2): We conducted a new analysis to investigate the seasonal pattern of pollinator visitation to key crops and wild plants. Through this analysis, we confirm that wild plants are supporting crop pollinators with floral resources outside of the main periods of crop flowering and are therefore likely to be facilitating crop pollination rather than simply competing with crops for pollinators. We present this new analysis in Figure S15 and refer to it in the Main Text and Methods.

4) Reframing interpretation of the modelled scenarios (suggested by Referee 3): We have reframed the MS (including in the title and subtract) to emphasise the share of nutrient intake and income attributable to pollination in the observed system, rather than focusing too strongly on the outcomes of the simulations. We have clarified why we think the three modelled scenarios remain relevant and useful in our study context and provided additional data to support this (new figures S7, S16). Nevertheless, we have softened our wording throughout the manuscript to frame the simulations as boundary estimates (rather than definitive forecasts) that illustrate what could happen under different trajectories of pollinator decline if no mitigating interventions are in place. Importantly, by making our assumptions more explicit throughout the MS, the simulations highlight where interventions could reduce risks, which is an important message of the paper.

5) Clarity and broader relevance: We have revised the Main Text, Methods and SI extensively to improve transparency (e.g., probability of adequacy calculations, retention factors, anthropometric calculations and yield-gap attribution) and have expanded the discussion of key results to place our findings in a broader context. We also implemented all minor edits suggested by reviewers (e.g., figure redesigns, terminology, references).

We attach our revised manuscript as well as a marked-up version (with tracked changes) and an updated Supplementary Information file. A detailed, point-by-point response follows below, with responses to each comment marked in bold font. The line numbers mentioned in our responses below refer to lines in the <u>clean version</u> of the revised manuscript.

Contents

Responses to Referee #1 2

Responses to Referee #2 4

Responses to Referee #3: 12

Responses to Referee #1

In addition to the few minor comments below, I have only one main critique. I feel that the study falls short of its analytical potential to identify quantitative differences in the pollinator community and associated changes in crop pollination and nutritional value. The authors collected plant-pollinator communities, and, for example, by employing a network approach (either bipartite between plants and pollinators, or multi-level across all 'interaction' types) the study could provide a more significant contribution towards the generalisability of the findings. More specifically, if differences in the structure of the interaction networks could be compared to patterns of nutritional benefits (or detriments) it would provide the wider community working with plant-pollinator communities in agro-ecosystems with a tool to draw conclusions between the structure of the ecological community and potential consequences for human health. I have the impression that not exploring these connections more systematically with, for example, a network approach (but other quantitative analytical approaches may equally work) is a missed opportunity that render this study less 'useful'. Is there scope for the authors to address this opportunity as part of this work?

We thank the reviewer for their thoughtful and encouraging comments, in particular for highlighting the potential to strengthen the generalisability of our findings through a more quantitative exploration of the structure of our plant–pollinator communities. In response to this suggestion, we added a new question/objective to our manuscript and carried out additional analyses focused on the relationship between network structure and nutritional outcomes.

First, we examined whether village-level differences in overall network structure (based on the full plant–pollinator interaction networks in each of our 10 study villages) explained variation in nutritional vulnerability to pollinator losses. However, the rate of nutritional decline was very similar across villages (Fig. S12), and no associations were found between village-level network structure and dietary outcomes. This consistency amongst villages does however give us confidence in the generalisability of our results.

We then examined the network roles of individual pollinator species and their associations with nutritional impacts. Our new analysis simulated the loss of individual pollinator species across the network and quantified the resulting decline in nutrient intake. We then tested whether different species-level network metrics (e.g., degree, nested rank, number of effective partners etc.) could explain the nutritional impact of each species' removal. This approach allowed us to identify which aspects of a species' position within the network best predicted its importance for sustaining nutrient intake. This new analysis found that several species-level metrics were

strong predictors of nutritional impact. In particular, species with a higher number of interaction partners (*degree*), greater network centrality (*nestedness rank*), and stronger, closer connections to other species (*weighted closeness*) were associated with larger declines in nutrient intake when removed. These metrics reflect species generalism and centrality within the network, suggesting that well-connected generalist species play a disproportionately important role in safeguarding dietary nutrient supply through pollination. The strength and consistency of these relationships indicate that species' network roles can serve as useful indicators of their functional importance to human health, providing a more widely applicable result than our previous analysis, which largely overlooked network roles and structure.

We have included the results of these new network-based analysis as an entirely new section of the manuscript (Question 3; Lines 262-277 in the Main Text and Lines 589-610 in the Methods) and provide a new Figure (S14) and Table (S8) in the Supporting Information. We believe this additional analysis substantially enhances the generalisability and practical relevance of our findings and we hope it addresses the insightful suggestions of the reviewer and the editor.

Here are a few minor points that may require further clarifications.

L89. I feel that the term 'changes in pollination service…' is a little vague and would perhaps benefit from a rewrite to be more specific.

We have now changed this to 'How could pollinator community changes impact human nutrition and livelihoods?' In response to your suggestions above, and those of Reviewer 2, we have changed the structure of our model to enable us to estimate the effect of various different scenarios of pollinator community change on human nutrition and livelihoods, so we believe this is a more appropriate way to word this question.

L165. Delete 'in'

Done

L358. This statement may require strengthening with some reference or data. While I don't question the general notion that year to year diet is reasonably similar, I'm wondering whether some information of interannual crop rotation for smallholder farms in this region would be available to support this claim.

Following your advice, we sourced government agricultural statistics (from the Ministry of Agriculture and Livestock Development) for Jumla District (the location of our study) and extracted annual production data for the five most nutritionally important crops/crop groups in 2021 and 2022. Comparing the data between the two years shows that the total production of key crops remained highly consistent, varying by a mean of 3.4% (maximum 14%) between 2021 (when pollinator surveys were conducted) and 2022 (when dietary recall surveys were conducted). Given that diets and farming practices are highly culturally conserved in this region, and that similar quantities of key crops are produced from year to year, we are confident that diets in 2021 and 2022 were comparable. We have summarised this new data and show the comparison between years in Table S11 as well as referring to it in the Main Text (L429).

L384ff (and/or Appendix S7). It's not entirely clear whether pollen on insects were identified in any way and/or whether only pollen grains of the last visited plant species of the specimen were counted. Given that heterospecific pollen loads may be high and conspecific loads may be low in some species (e.g. generalist pollinators with low floral fidelity), some distinction about the type of pollen may be needed here. Please clarify in the text, perhaps just by emphasising that this measure of pollinator importance is not specific to plant species.

Apologies for not being clearer in our explanation of the pollen load analysis. We did not identify individual pollen grains to species but instead counted the total number of pollen grains (of all plant species) on replicate individuals of each insect taxon to give us a measure of the insect's pollen carrying capacity. This reason for this additional data collection was to enable us to take account of the substantial interspecific variation in pollen transport ability, rather than treating all insect taxa as equally good pollinators. For example, we did not want to equate a visitation event by a butterfly (with only 4 or 5 pollen grains on its body) with a visitation event by a bumblebee covered in hundreds, or thousands, of pollen grains. We have now clarified this point in the Methods (L464-467) and supplementary text S8 and emphasised that our measure of pollen carrying capacity is specific to each insect taxon, but not each plant species.

The new addition to the Methods is as follows: "*Pollen grains were not identified to species (impractical in a study of this scale), so our measure of pollen carrying capacity is limited to one value per insect taxon, rather than specific values for each plant-pollinator combination.*"

Responses to Referee #2

This is an impressive paper linking pollinator services to human income and nutrition. In general, the paper is very well-written, and all claims are backed up properly. I especially appreciate the detailed repository on GitHub, making all results reproducible. Adding a DOI (e.g. via Zenodo) to the repository would make it citable and permanent.

We thank the reviewer for these positive comments and their many excellent suggestions, which have undoubtedly made the paper much stronger and clearer. We will certainly add a DOI via Zenodo once the revisions are completed and the code is finalised.

Given the complexity of this very well crafted paper, I have some minor comments aimed at clarifying some decisions taken, or asking to add some caveats in the discussion. I include those as "in-line comments" below. The only major caveat I have when reading the paper is the comparison with the different pollinator trend scenarios. I understand that the "no pollinators" scenario is a straightforward comparison to make, but it is a scenario that can be not too informative. Despite the Chinese example cited (which is a more complex story than just losing pollinators), losing pollinators completely is an implausible scenario, especially in a heterogeneous landscape such as the one studied. In fact, the "pollinator reduction" scenario is also quite simplistic, and assumes a declining rate equal for all pollinators based on the recent decline in honey harvesting. This decline might be a good proxy (and I like how it is justified), but it is also a strong, untested assumption, especially as many species in the network are solitary. Finally, the "enhanced pollination" scenario also relies on complex assumptions (see specific comments below on that). I want to stress that all three scenarios make sense and are correct, and hence, if the authors want to keep them (while adding a few caveats to the text; see in-line comments), I am fine with this decision. However, I would prefer to focus on a continuous "pollinator rate of change" scenario and test different levels of increases/reduction (e.g.,

from +50% to -50%, which is justified by the literature). Ideally, this can include some response diversity across pollinators (e.g., the decline rate for each species is drawn from a normal distribution with some variability). As predicting pollinator changes in the next years is a daunting task, providing the numbers for a range of situations could be more informative. Again, I want to stress that this is only how I would do things, in case the authors find it interesting, but the 3 scenarios presented are valid and justifiable.

We thank the reviewer for this important and constructive comment regarding the framing of the pollinator change scenarios. We fully agree that the original three-scenario framework (i.e., no pollinators, business-as-usual decline, and enhanced pollination) represents a simplification of reality and involves assumptions that are difficult to validate, particularly regarding the homogeneity of pollinator responses across diverse taxa.

In response to the reviewer's suggestion, we have revised our model structure to allow for a continuous, probabilistic simulation of pollinator decline which incorporates response diversity among species. Specifically, we simulate overall declines in pollinator abundance in 10% increments, from 0% to 100% loss of pollination services. For each level of overall decline, we draw the decline rate for each individual species from a normal distribution with the target mean decline and a standard deviation of 0.2, thereby reflecting variation in species' sensitivity to environmental change. We run 100 simulations for each level of decline and report the mean and 95% confidence intervals of the resulting changes in nutrient intake. This new modelling approach allows us to present a continuous linear relationship showing how dietary intake of key nutrients is expected to change across a gradient of pollinator decline scenarios. We have re-written the relevant sections of the Main Text (L180-198) and Methods (L513-566) to explain this new modelling approach and have added a new Supporting Information figure (Fig. S11) showing the full response relationships for the six most pollinator-dependent nutrients.

While this new continuous decline framework provides a more flexible representation of pollinator loss, we believe it is still useful to retain the three original scenarios (complete loss, business-as-usual decline, and enhancement) in the main text because they provide clear, discrete reference points that are more tangible and communicable for policymakers, practitioners, and non-specialist audiences. They also allow us to visualise the distribution of impacts across individuals in a way that is difficult when looking at continuous rates of decline. However, we now clearly emphasise that these scenarios are theoretical rather than predictive and have added a number of caveats and justifications throughout the Main Text and in supplementary text S11 & S12 (also see our responses to some of the line-specific comments below).

<u>in-line comments:</u>

Line 65: It is unclear to me from the citations if pollinator decline risk is higher in areas with small-holders. Maybe you can explain why (e.g., in comparison with areas that suffer from agricultural intensification) or tone down this claim.

Apologies - our original wording was unclear. We did not intend to claim that the risk or probability of pollinator decline is higher in these farming systems. Instead, we were saying that the <u>impacts on humans</u> resulting from pollinator decline are expected to be greater as people in these farming systems are poorer, more reliant on their crops for nutrition and lack the flexibility to adapt. We have adapted the wording of this sentence to make our point more clearly (L62).

Line 80-83: In the main text, you do not mention that the Jumla pollinator trends mentioned are based on honey harvest statistics. I would use this info with caution, as honey production per hive does not imply fewer hives, nor is it extrapolable to other social and nonsocial species. Again, I would tone down what we know about pollinator declines in the area.

We have revised the sentence in the main text to moderate our level of confidence in these trends and clarify that they are based on just one pollinator species (L80). The study we cite (Kortsch *et al.* 2024) recorded both the number of occupied honeybee hives and per-hive honey production, and both showed a steep and closely correlated downward trend. We agree that these data should be interpreted with caution, as honeybee colony performance may not reflect the status of other social or solitary pollinators. However, there are no other datasets available to indicate pollinator trends in this region, and the honeybee data at least suggest that major stressors are operating, even if not all species are equally affected. Nevertheless, we have moderated the sentence to avoid overstatement of our confidence in these trends (L80).

Line 105: This is extensively answered below, but I was wondering here if these crops are locally consumed, and a hint here might help the reader.

We've added a few words to this sentence to clarify that these crops are locally consumed (L 104)

Figure 3: I wonder if it's worth combining these two plots. I spent some time linking both mentally, and if I understand correctly, the orange bars in a) can be integrated into the green bars in b). Just a suggestion if you think it enhances the visualization.

This is a good suggestion; we have now combined these two panels into a single figure which we believe improves the clarity of visualisation.

Line 157: Again, you don't know pollinator declines in your area, you can keep the reference, but explain the details a bit more to avoid misleading claims.

We have revised the sentence in the main text (L164) to clarify that the evidence for pollinator decline in our study region is based on data from native honeybee populations only (Kortsch *et al.* 2024). We now present this as indicative of potential pressures on pollinators rather than as direct evidence for broader declines, thereby moderating our wording to avoid misleading claims.

Line 166: Given the severe under-nutrition in the area, I would appreciate a quick statement on what other factors (other than pollinator) are at play. Even when you show that pollinators can alleviate the situation, even the best pollinator scenario shows nutritional problems for the population. This can be highlighted in conclusions, for example.

We have added a new paragraph to the Main text (L174-179) to acknowledge that undernutrition in our study population reflects a combination of food system and health-related challenges, including low agricultural productivity, limited access to inputs and extension services, reliance on local production without adequate market or storage infrastructure, and the increasing availability of cheap, nutrient-poor processed foods (Rasul *et al.* 2018). These pressures are compounded by suboptimal infant feeding practices, recurrent infection, high

energy demands from physically demanding livelihoods, and uneven access to health and nutrition services. Against this background, we now frame pollinator decline as an additional potential challenge that we aim to quantify in this study.

Line 171-173: Until reading the methods, how "decline" is extrapolated, and what "optimal" means, is unclear. Here is where a continuous scale of pollinator changes would help me understand this directly.

We have completely revised this section of the main text (L180-220) to align with our new modelling framework (based on continuous rates of decline), and in doing so have clarified both what we mean by "decline" (extrapolated rates based on local honeybee populations) and by "optimal" (each crop achieving its maximum pollinator-dependent yield). We also now frame the 'business-as-usual' case as an illustrative scenario rather than a precise prediction.

Line 187: "persists even when consuming below…". This part is confusing to me; maybe changing the wording can help (or maybe it's only me).

We have revised the sentence to simplify the wording, now stating that although many women in our study population already consume less than the estimated requirement, further reductions in intake are expected to further increase the risk of neural tube defects in children in utero (L212).

Line 205: Here, the % of "no pollinators" and "declining pollinators" are hard to compare as you need to move between paragraphs. I understand that from 44% for no pollinators it goes to 14% for declining pollinators, and for nutrients it drops from ~22% to ~6%. I find more relvant the second scenario, and would focus on this one.

We have revised the text to integrate the "no pollinators" and "declining pollinators" scenarios into the same paragraph (L196-205), making it easier to compare their relative impacts. We now clearly frame the complete pollinator loss as a worst-case benchmark and the 'business as usual' case as a more conservative but realistic estimate, based on a best-guess extrapolation from historic honeybee decline data. We also indicate that the latter provides a more plausible indication of likely impacts in the near-term.

Line 213: Here, one wonders what else is limiting crop production. Temperature, fertilizers, pests? Is pollen limitation a relevant issue in comparison with these other drivers?

It is certainly true that pollination is not the only factor limiting crop production in this region; impoverished soils, low winter temperatures, limited access to fertilisers and improved varieties, and pest pressures are also likely to be important constraints. In reality, yield gaps arise from a messy mix of these factors, of which pollination is only one part. Our calculations take this into account by only closing the portion of the yield gap estimated to be attributable to pollination. Specifically, we base our estimates on Garibaldi *et al.* (2016), who demonstrated across a wide range of crops and regions—including Nepal—that while much of the yield gap is driven by factors such as fertiliser and water availability, pollination deficits nonetheless account for an important and predictable share in many smallholder farming systems. These findings are supported by data from this region of the Himalayas (e.g. Partap *et al.* (2012), which show strong indications of pollen limitation in apple production. While many other constraints are

difficult or expensive for farmers to address (e.g. temperature, fertiliser), pollination is one factor that farmers can feasibly improve themselves, making it important to quantify. We have clarified this point in the revised text (L309-313).

Figure 4: I would not reuse colors from Figure 3 with a different meaning. I like how the gradient is presented here, in line with my main comment above. In contrast, Figure 5 capitalizes only in the no-pollinators scenario.

We have altered the colours of Figure 4 to avoid overlap with Figure 3. We have also replaced Figure 5 with a new figure to show the full spectrum of potential nutritional outcomes associated with changes in the pollination service. Instead of showing a single simulated outcome of pollinator decline, this figure now illustrates the full range of possible outcomes and the degree to which pollination can shift adequacy for key nutrients.

Looking at the figure, I wondered why adolescent men are not targeted. I wonder if the assumption is that they equal adult men regarding diets; if so, this can be stated in the methods. It is just a curiosity.

The assumption in public health research is that adolescent boys are less nutritionally vulnerable, and their dietary patterns are more similar to those of adult men. In contrast, adolescent girls are a subgroup of global policy and programmatic focus both due to concerns about gender-based discrimination, and because many adolescent girls will soon have their first child (and it is well established that pre-conception nutrition is a major determinant of maternal and child nutritional outcomes). We have now clarified this in the methods section (Line 355).

Line 248-241: Here, I would love to see if wild plants are visited before and after the crop (indicating clearly they support pollinators complementary to crops) or simultaneously to crops (where competition might (or might not) be occurring).

This was an excellent suggestion, and we now explicitly examine whether visits to wild plants occur before/after crop bloom or simultaneously. Using the two dominant crop pollinator groups as examples (*Apis* spp. and *Bombus* spp.; these are the only groups with a sufficient resolution of data), we analysed the phenological pattern of visitation to the three most nutritionally/economically important crops (apples, mustard and beans) and compared this with the visitation of wild plants. For both pollinator groups, wild plant visitation is elevated outside the main flowering periods of each crop (pre- and post-bloom), with only modest overlap at the shoulders of crop peaks. This pattern indicates that wild flora are providing complementary floral resources that support pollinators between crop blooms, consistent with facilitation rather than strong temporal competition. We allude to this new analysis in the Main Text (L299), Methods (L828-631) and have added a new figure to the supplementary information (Fig. S15).

Line 257: A piece that is missing is which is the plant contribution to pollinator fitness. This is (almost) impossible to get, so it is ok, but can be stated as a caveat. Here, the assumption is that it is proportional to visitation rates.

This is a good point and we have now added the following paragraph to the Methods (L625-628):

This approach assumes that a plant's value to pollinators is proportional to how often it is visited, without accounting for the nutritional quality of floral resources or how much is consumed during visits. Our estimates should therefore be interpreted as indicative rather than precise.

Line 160: I would be cautious in recommending increasing beekeeping, as it can cause collateral problems. In addition, what about the effects of weeds (which support pollinators) competing with crops, or sacrificing space needed to grow crops to plant ornamental plants? I think this sentence can be more balanced, showing pros and cons.

We agree that beekeeping can often cause problems for native ecosystems, so have moderated our wording to be more cautious in our recommendations. To draw a distinction between the small-scale beekeeping with the native honeybee *Apis cerana* that occurs in our study region, and the promotion of commercial *Apis mellifera* elsewhere, we have now clarified that we refer specifically to *small-scale* *native* beekeeping. We also highlight that any management actions (including beekeeping) must be assessed by farmers themselves to avoid negative ecological impacts (for example, disease transmission by honeybees) (L302-308).

With regard to wildflowers, weeds, and ornamental plants, we agree it is important to acknowledge potential trade-offs with crop production. In the revised text we now state explicitly that all such management strategies must be assessed by farmers themselves to minimise potential ecosystem disservices (e.g. competition with crops for space or resources) and to ensure they are appropriate to the local context (L302-308). At the same time, evidence from our study region indicates that farmers are willing and able to adopt these practices at minimal cost once they understand the value of pollination services – we now reference this in the main text (L307)

Line 264: Flower strips work for conservation, but do not always have economic benefits (e.g., work from David Kleijn group).

This is true - the economic benefits are not universal. We have removed this sentence to avoid making any inaccurate claims.

Line 339-340: But pollinators are also expected to enhance fruit quality (e.g., micronutrients), so your estimates from the literature are conservative.

This is a good point; we have now added the following sentence to the main text (Line 141):

Our estimates are likely conservative, as they only consider the contribution of pollinators to crop yield, and not the additional benefits to fruit and seed quality, including micronutrient content (Gazzea et al. 2023).

Line 343: I am impressed with the amount of careful information gathered. Were the participants remunerated? I am just curious about this (and if they were would be good to state this).

Indeed, it was a great deal of work and a large time contribution for the participants! Their time was remunerated with vouchers for mobile phone credit. This approach has been used in similar studies in Nepal, as it provides participants with a valued and widely usable resource

while being considered less coercive than direct cash payments. We have now mentioned this remuneration in the methods (line 366).

Line 379: By "visiting a flower," you refer to making contact with the plant's reproductive parts? If so, this would indicate potential pollination with a higher degree of confidence.

In our training programme, data collectors were taught the principles of pollination ecology, including floral reproductive structures and the mechanics of pollen transfer. They were instructed to record an interaction only if the insect was observed collecting pollen or nectar from the flower. Given the clear guidelines and extensive training, we are confident that the interactions recorded by our data collectors at least had the potential to result in pollination. We have added an additional note to the Methods to clarify this (Line 436).

Line 465: What about crops such as carrots? (which are pollinator-dependent for seeds only).

In our study system, farmers are rarely able to access or afford external seed sources, so crops are sown with their own seeds saved from the previous year. Thus, we consider crops as pollinator-dependent if their seed production relies on animal pollination, even if the edible portion of the crop does not (e.g. carrots and mustard greens). This is stated in Lines 492-494 of the methods and we have now added a new Supporting Table S10 which includes data showing where the study households obtained their seeds from (97% from their own production; 3% from neighbouring farms).

Line 472: As stated above, honey per hive does not necessarily relate to number of individuals, and other environmental variables might explain this. In addition, social dynamics are not comparable to solitary lives. In addition, while the linear assumption between visitation and production is straightforward, it is unlikely to be the best functional form, as yield should saturate with visitation (also line 488). Maybe this can be mentioned.

We have now completely revised this section of the manuscript to align with our new modelling framework, which incorporates response diversity among pollinators and estimates impacts across a continuous gradient of decline (L180-199). Within this framework, the "business-as-usual" case is retained only as an illustrative scenario, and we now state explicitly that it is derived from extrapolations of local honeybee hive numbers and honey yields and should be viewed as indicative rather than predictive. We also clarify that extrapolating honeybee trends to wild pollinators, particularly solitary species, involves major assumptions (L541-547).

Regarding the functional form of the visitation–yield relationship, we agree that a saturating rather than linear function is more realistic. In the revised text, we have now clarified that while we modelled yield responses to pollinator decline linearly, our estimates of potential yield increases from pollination management do not extrapolate this linear relationship. Instead, they assume that only a certain proportion of local yield gaps can be closed by enhancing pollination services, following the approach of Smith *et al.* (2022) - see Lines 550-566.

Line 485: But on the other side, you assume a common response across pollinators, while it is well known that there is response diversity across pollinators. In fact, your network approach is perfectly suited to explore the role of response diversity in buffering the effects (e.g. by testing declines with/without response diversity), but I understand if this is beyond the scope of the paper.

This was an excellent suggestion, and indeed we have revised our modelling framework to incorporate response diversity among pollinators, allowing species to decline at different rates (drawn randomly from a normal distribution around the mean rate of decline) rather than assuming a common response. This is explained in more detail in some of our previous responses and is now incorporated into the Main text (L183-185) and Methods (L528-531).

Line 498: This part needs details. Yield gap can be caused by many factors, and how do you decide which fraction is attributed to pollinators needs explanation. In supplementary material is also unclear (see below).

We have completely re-written this section of Methods (L550-566) and added further detail to the Supporting Information Appendix S12 to clarify how we estimated pollination-related yield gaps. Specifically, we now explain how current yield gaps were calculated within our study region, and that only the pollination-related share of these gaps (estimated at a median of 24% in smallholder systems; Garibaldi *et al.* 2016) was assumed to be recoverable through pollination. We clarify that the remainder of the yield gap, attributable to other agronomic constraints (e.g. fertiliser, water, soil quality), was left unchanged. We also emphasise that our estimates of pollination deficits align closely with a global assessment of pollination deficits (Turo *et al.* 2024), which provides confidence in the validity of this approach.

Line 505: Here is "mean yield", right? I would make it explicit.

This whole section of the methods has now been re-written for clarity.

Line 532: Version of R is more relevant than that of R Studio (which is just a graphical interface for R).

Good point - this is now changed to the correct version of R (L640).

Supplementary Text S8. Pollinator exclusion experiments: I am surprised that you have data on hand pollination too (which is not used). I wonder why not using this data to calculate "optimal pollination". This would simplify your assumptions, as the difference between hand and open pollination is clearly attributable to pollen limitation.

The initial purpose of these experiments was indeed to compare hand pollination with open pollination in order to estimate pollination deficits directly. Unfortunately, this aspect of the experiment was not implemented correctly. Covid-19 lockdowns coincided with the training and set-up period, preventing key staff from travelling to the site. The experiment was implemented regardless (because otherwise the growing season would have been missed) and as a result, the hand pollination procedures were not applied correctly. In many sites flowers were damaged during manipulation, leading to aborted flowers and no fruit development. Fortunately, the simpler pollinator exclusion treatments were carried out correctly and provided usable data, but we were unable to use the data from the hand pollination treatments. We should have initially clarified this when referring to these experiments, but we have now added some text to this section of Supplementary Text S8 to explain this issue.

Supplementary Text S11. Predicting yield increases from enhanced pollinator abundance: Here, I understand that you assume 24% of the yield gap is attributable to pollinators. This would need a bit more justification. It can also be reflected better in the main text.

We have revised the Methods (L550-566) and Supplementary Text S11 to provide additional justification for how we estimated the pollination-attributable share of yield gaps. We now explain that the 24% figure is drawn from Garibaldi et al. (2016) who quantified the contribution of pollinator abundance to yield gaps by relating yield variation to flower visitor density after accounting for other agronomic inputs. We also clarify that in our modelling we only reduce the portion of yield gaps attributable to insufficient pollination, while recognising that the larger portion caused by other management constraints (e.g. fertiliser, water) remains unchanged.

Responses to Referee #3:

In response to question 1, the paper shows how pollination deficit and adequacy of the diet are connected and quantifies the contribution of pollinators to dietary intakes. The strengths of the paper include primary data collection for four different populations with quantitative dietary assessment every month for a year, with indication whether each food was locally obtained. This allowed to quantify micronutrient intake coming directly from pollinator-dependent local or imported foods. Using a thorough survey of plant visitation in the same study area a year earlier (lack of overlap was due to COVID19 restrictions for household surveys), the authors also quantified the yield of these foods attributable to a range of local pollinators, allowing them to calculate the contribution of each pollinator to micronutrient intake. Findings from these complex primary data analyzed in an original way deserve to be disseminated, as the authors make a very concrete and convincing connection between pollination and healthiness of the diet.

I am less convinced by the interpretation of the simulations used to respond to questions 2 and 3 because they are based on multiple rather unrealistic assumptions of proportionality (e.g. that intake of any crop would vary proportionally to its production). These assumptions are very transparently acknowledged. Although this seems fine to use these assumptions to quantify which share of the observed income and nutrient intake is attributable to pollinators, the interpretation of these attributable fractions as reduction of nutrient intake or wealth which resulting from changes in pollinators effectiveness is less convincing. The message would be more convincing if it interpreted these results as the share of income, PA or micronutrient intake attributable to pollination in the observed scenario, rather than trying to model a change when the moving variables are unlikely to be independent from each other. Alternatively, if data allow, the modification of these assumptions to rely on more sophisticated elasticities may be more appropriate and more convincing.

We thank the reviewer for their positive and constructive comments which highlighted a number of important methodological points which required further clarification or justification. In our responses to your comments below, we explain how we addressed each of these points in the revised manuscript.

Regarding the point about proportionality, we agree with the reviewer that in many economic systems, particularly those with well-functioning markets, the assumption of proportionality between production and consumption is problematic. In such systems, we find weak linkages between production and consumption. Production changes may be absorbed by: 1) Multiple market channels (e.g., export and processing); 2) Consumer substitution of unavailable goods

with alternatives; and 3) Variable price responses, which in turn affect consumption differently between households with varying food budgets.

In those contexts, demand systems that estimate own- and cross-price elasticities are indeed appropriate to capture complex, interlinked relationships between prices and consumption of multiple goods. However, we believe these models are not well-suited to our study setting in Jumla, Nepal, for the following reasons (which we demonstrate through new analyses presented in the Supporting information):

1. Market channels are extremely limited in Jumla

In many settings, production surpluses may be absorbed by other channels, for example, export, food processing, or sale. However, like many other smallholder farming regions (Markelova *et al.* 2009), Jumla's geographic isolation, poor infrastructure (e.g. lack of storage, transport and processing facilities), and low purchasing power create a highly constrained food system that behaves very differently from textbook open-market systems. Very little food is imported or exported, as demonstrated by our new analysis of the household survey data which show that >85% of household crop production is directly consumed by the household members themselves, rather than being sold, gifted, traded or fed to animals (Figure S15). This makes it difficult to define market prices for many crops. Under this constrained market system, the proportionality assumption is more likely to reflect reality than price-based models of demand systems, which assume market reallocation of food surpluses.

2. Consumer substitution is not feasible due to market constraints

In more integrated systems, consumers may substitute unavailable or expensive foods with similar alternatives. In contrast, more remote regions with limited infrastructure (e.g. lack of reliable road networks and cold storage) have prohibitively high transaction costs. This drives very weak market integration, particularly for perishable, micronutrient-rich foods such as fruits, vegetables, leafy greens, and animal-source foods. Figure S6 demonstrates this, showing more than 90% of the total intake (by mass) of these five nutritionally important food groups comes from local production. Only non-perishable staples (primarily rice and cooking oil) are routinely imported into Jumla. These staples are nutritionally distinct and cannot substitute for the micronutrient-rich foods that are locally grown. Thus, when the availability of pollinator-dependent micronutrient-rich foods decline within villages of Jumla, there is no viable market-based replacement, making proportional changes in consumption much more plausible.

This point is emphasised by the substantial seasonal variation we observe in the intake of these food groups, and in micronutrient intake overall. Figure S7 shows dramatic seasonal fluctuations in the intake of leafy greens, vegetables, pulses, and fruits, which are produced and consumed locally. These fluctuations lead to up to five-fold variation in the intake of key micronutrients such as vitamin A, vitamin C, and folate. Such patterns are consistent with a food system in which consumption is strongly constrained by local availability. In particular, the consumption of key food groups (especially perishable, micronutrient-rich fruits and vegetables) increases rapidly after harvest time and then declines as local availability declines. This strongly suggests unmet demand for these foods and supports the assumption that declines in production lead to proportional declines in consumption. The only exception to this is cereals (primarily polished white rice) whose consumption remains constant throughout the year because it is one of the few items that is regularly imported.

3. Budget disparities are minimal

Conventional demand systems also account for the fact that price changes affect consumers differently according to their budgets. In Jumla however, households do not rely on local

markets to sell produce as a source of income (as indicated by the earlier statistics showing >85% of crop production is directly consumed by the household; Fig. S15). The only cash crops of any significance are apples and beans, which are sold externally into relatively stable regional markets. Local budgets are therefore not tightly linked to local production levels. Moreover, our study population has minimal wealth disparities which further reduces the likelihood that changes in yields would drive divergent dietary responses across income groups. In summary, demand variability driven by price elasticity is unlikely to be a major driver of food consumption patterns in this setting.

Given the points outlined above, we believe that introducing demand system elasticities would require strong, and likely untrue, assumptions about consumer substitution, price sensitivity, and market access that are not supported by the realities of Jumla. In this context, we believe a proportional approach more accurately reflects how changes in pollination-driven production translate into changes in dietary intake and income.

However, we understand the reviewer's caution, and to address this, we have followed their advice and reframed our interpretation (including in the abstract) to focus on the attributable share of nutrient intake and income derived from pollination services. We have retained the simulated scenarios because we believe it is important to illustrate the range of possible future scenarios and provide land managers and policymakers with a tangible sense of these outcomes. However, we have softened our wording throughout the manuscript (including in the abstract; L35-36) to frame the simulations as boundary estimates (rather than definitive forecasts) that illustrate what could happen under different trajectories of pollinator decline if no mitigating interventions are in place. While these scenarios inevitably involve assumptions, they are not improbable in the context of Jumla (as we explain in detail above). Importantly, by making these assumptions explicit, the simulations highlight where interventions could reduce risks, for example, by strengthening market access to improve the availability of micronutrient-rich foods, supporting the cultivation of alternative crops, or providing economic safety nets for farmers. We have provided a substantial amount of additional justification for our approach in the Methods (L568-587) and Supporting Information (Appendix S1, Figures S5-S7 & S15).

In addition to these overall comments, I provide detailed comments on the dietary intake methods, which I was specifically asked to review, and found appropriate overall. Data collection methods were strong. However, the calculation of nutrient intake would need to account for micronutrient retention factors if it did not, or the paper needs to describe that it did. Finally, the calculation of probabilities of nutrient adequacy was appropriate but needs to be described in the paper. More details are provided below.

My major concern related to diet data management methods is that the authors did not describe if micronutrient retention factors relevant to the cooking method were accounted for. When food is cooked (e.g. boiled, steamed, stewed, etc), loss of 5-25% of micronutrient content is common, and it can go up to 50% for certain micronutrients and certain foods. Published tables of these retention factors exist in the literature. Loss of nutrients due to cooking may significantly affect the intake of these nutrients. I don't know how the results presented in the manuscripts may be affected by this. I recommend the authors should account for nutrient retention factors and/or clarify that they did.

Thank you for raising this important point and we acknowledge we did not make this clear in the methods. We did account for nutrient retention factors in our dietary intake calculations. Specifically, we used the USDA Table of Nutrient Retention Factors, Release 6 (U.S. Department of Agriculture 2007). For each ingredient in our recipe database, the food composition value for each nutrient was multiplied by the appropriate retention factor

according to the cooking method used (e.g., boiling, frying, steaming etc). Ingredients cooked by different methods were treated as separate entries in the database (e.g., boiled potato and fried potato as distinct rows), allowing us to apply the relevant retention factors. We have now added some additional text to the Methods (L396) and Supporting Information (S4) to make this clear.

A second major concern is that the author did not describe at all how they calculated probabilities of dietary adequacy from observed nutrient intakes. The authors need to add a summary of the process in the main methods. I reviewed the R code to get the description and reviewed how this was doneI understood that the mean of the 12 records for each individual was assumed to approximate usual intakes for all nutrients presented in the paper. Due to large within to between-person variability in micronutrient intake, a large number of observation days is needed to adequately approximate usual intake by a simple mean. The exact number of days required for this varies ; the literature cite ranges between one and several dozens of days. The study dataset with a dozen observation days across a year for everyone is unique, and probably good enough to get a reasonably reliable estimate of usual intake with a simple mean. However, the authors may want to briefly justify and discuss this using relevant references. Then, the probability approach used to obtain probabilities of adequacy from usual intake is strong. However, it needs to be reported in the paper and the reference provided.

Also, I would like to know which dietary requirements were used and which levels of bioavailability were assumed for zinc and iron. Detailed supplemental text may be deemed useful to describe specificities such as iron adequacy calculation (skewed distribution of requirements was assumed), bioavailability for iron and zinc, and references for nutrient requirements considered (EAR and CV or SD).

We apologise for not providing greater clarity on our probability of adequacy (PA) calculations and bioavailability assumptions. We have now added a new section to the Methods (L409-420) and a detailed explanation in a new section of the Supplementary Information (Supplementary Text S6). Briefly, participants contributed up to 24 replicate recalls across a year, allowing us to estimate usual intake using simple means. Given the relatively large number of repeated measures, this approach should provide a reasonable approximation of usual intake, although some within-person variation may remain (Nyambose *et al.* 2002). PAs were then calculated using the standard probability approach, with iron treated separately due to its skewed requirement distribution. Estimated Average Requirement (EAR) values were applied according to age, sex, and physiological status at recall, with low bioavailability assumptions for iron and zinc, as recommended by FAO/WHO (2004). We have provided all this information in much greater detail in our new Supplementary Text file (S6).

Finally, though the title is punchy, I have concerns with it. First, the use of "health" in the title is misleading, it implies that some health indicators of individuals were measured and associated with pollination deficits, which is not the case. I suggest replacing health with "healthiness of the diet". Second, as explained above and below, the limitations due to pollen deficits result were simulated based on strong/simplistic assumptions. The title should either clarify that this is based on simulations, or turn it the other way around, stating that pollination contributes to healthiness of the diet and wealth of impoverished communities.

The reviewer is correct to point out that our original title could be misinterpreted, as the links between pollination deficits and health outcomes are based on simulations and not direct health measurements. In response, we have revised the title to highlight the positive contribution of pollinators rather than the consequences of their loss. We believe it is still appropriate to retain the term 'health' because our results demonstrate and quantify the critical role of pollinators in

supporting the production of key micronutrients that are essential for human health and insufficient in these communities. The negative health impacts of insufficient micronutrient intake is well known (particularly for our two focal nutrients, vitamin A and folate) and as such we feel it is justifiable to claim that pollinators are directly contributing to both the health and the livelihoods of people in these settings.

Our new proposed title is: *"How pollinators support the health and wealth of vulnerable communities"*, however we have deferred to the editor for the final decision.

We believe the '*how*' is important in this new title because an important aspect of the study's novelty lies in the mechanistic links we quantify between pollinators and human nutrient intake.

Specific comments on dietary methods and results:

Main text, line 334. As I don't see any mention of yield factors, I assume the proportional composition of each ingredient was based on raw weight. Please clarify in the sentence "calculated based on raw ingredient weight" (or refer to the use of yield factors if they were used).

We confirm that the proportional composition of each dish was derived from the weights of the raw ingredients used in recipe preparation, while accounting for the addition of water and inclusion of only the edible portions of foods (e.g. excluding stalks or seeds not used in the dish). Nutrient values were then adjusted for cooking-related changes using the USDA Table of Nutrient Retention Factors, Release 6 (U.S. Department of Agriculture 2007), to ensure that calculated intakes reflected the nutrient composition of foods as consumed. We have now clarified this in the main text (L390).

Main text, line 340. References for published Food Composition Tables are missing.

Apologies for this oversight. Our Nepal-specific food composition table (FTC) was compiled from a range of sources following a hierarchical decision-making process whereby values from a nearby region (for example India) were prioritised where available, and then more distant values (for example from USDA) were used when needed. We have added a new paragraph to Supplementary Text S4 to outline this selection process and cite all of the FCT sources we used. Additionally, we have included the specific FCT reference for each ingredient in the main dietary recall data file, which is freely available online: https://doi.org/10.5285/d7434d83-c30d-4186-aab0-9764821cd807

Main text, line 342: please describe how probability of adequacy was derived from nutrient intake across multiple days.

We have now added a new section to the Methods (L409-420) and an entirely new Supplementary Text file (S6) to explain in detail how probability of adequacy calculations were made. Please also see our replies to the earlier comments above.

Line 402: add reference of published food composition tables.

We have now referenced all of the FCT sources in a new section of Supplementary Text S4 as well as describing the process by which each FCT source was selected. Please also see our reply to the earlier comment above.

Lines 419-420: Do you have data or a reference showing the magnitude of the use of own seeds versus imported seeds?

Yes, this was included as a question in our farmer questionnaire. For key pollinator-dependent crops, we asked the farmers where they sourced their seeds. The results showed that 97% of farmers sourced their seeds from their own crop whilst the remaining 3% sourced them from other people's crops nearby. Out of 200 respondent households, not a single one reported purchasing seeds from outside their village. We have now clarified this in the Methods (L491-494) and added these statistics to a new summary table (Table S10).

Lines 448-460 and supplementary text S1, assumptions: This study has data that could support assumption 1, or refute this assumption. Study data could be used to show the variation in consumption of a crop depending on variation in yield of this crop in the population. I am not an expert in simulations, but wonder if it would be feasible for authors to use study-specific elasticities of crop intake depending on crop yield, or better, elasticity of micronutrient intake depending on a number of crops yield? This would allow to get rid of strong assumptions 1 and 3, which may not be realistic. My specific expertise does not allow me to assess the plausibility of other assumptions. However, these assumptions would appear more convincing if supported by more references or data. Assumptions of proportionality seem simplistic. Also, an assumption is not presented, the assumption that crop yield declines proportionally to the decline in pollinator*pollinator dependence value. This is explained at the end of supplementary text S10 but should be presented as an assumption.

Following the reviewer's earlier comments, we have revised the wording of our assumptions (L568-587) and made changes to our modelling framework to clarify and moderate our interpretation of the simulation results.

As the reviewer notes, ideally one would estimate study-specific elasticities linking crop yields to crop or micronutrient consumption. Unfortunately, our dataset does not include detailed agricultural production data at the household level, which precludes the direct estimation of such elasticities. However, we do have information on the seasonal timing of crop production/harvesting and the timing of consumption which we have added to the Supporting Information (Figure S7). These data reveal a strong seasonal synchrony between availability and intake of key pollinator-dependent foods (particularly fruits, vegetables, and leafy greens). This supports our interpretation of Jumla as a food-limited system in which production and consumption are tightly linked.

We also now provide a much clearer rationale for each assumption in the Supporting Text S1, and we have moderated our wording throughout the manuscript to avoid overstating precision. Rather than presenting the simulations as forecasts, we now frame them as boundary estimates that illustrate the potential scale of impacts if proportional relationships hold, and which can therefore highlight the possible consequences of pollinator loss. We also now explicitly present the assumption that crop yields decline proportionally to pollinator visitation × pollinator dependence so that all key assumptions are stated up front (L570).

Figure 2: it is a very nice figure visually. However, it is unclear to me what the size of a crop bar represents. Is this the sum of all contributions to micronutrient intakes for all micronutrients together? This is what it seems to indicate but I am not sure it makes sense. I also wonder which unit are used to figure micronutrients' flows, is this percent of total micronutrient X intake?

Apologies for not making this clearer, we have now added some additional text to the figure legend to spell out what the bar and flow widths represent. In short, the crop bars in the figure show the sum of the crop's proportional contributions to each key micronutrient. They therefore represent their relative importance in providing these six key pollinator-dependent micronutrients. And yes, the flows show the percentage of each of the micronutrients that is provided by that crop. For example, you can see that Jumli beans (brown flow) provide approximately half of all the folate in people's diets.

Figure 3: it is difficult to assess how much pollinator dependence is local or imported. Would it be useful to combine both panels into 1 figure with 4 categories?

This is a very good suggestion and one made by Reviewer 2 as well, so we have changed the figure accordingly.

Figure 4: vitamin E is presented everywhere else except on this figure.

Thanks for pointing this out – we have now added vitamin E to this figure.

Figure 5: Y-axis graduations are not harmonized. Are the differences significant and meaningful between original and pollinator loss (see e.g. Calcium and vitamin A)? In line with my comment right above, this figure shows the PA attributable to pollinators (Original PA minus PA with no pollinators).

In response to your important comments about our modelled scenarios, we have now replaced Figure 5 with a new figure to show the full spectrum of potential nutritional outcomes associated with changes in the pollination service. Rather than showing a single simulated outcome of pollinator decline, this figure now illustrates the full range of nutritional adequacy outcomes attributable to pollination and highlights the degree to which pollination could shift adequacy for key nutrients, if no mitigating actions are taken. It is true that for some nutrients (such as calcium and vitamin A), probability of adequacy is already so low that additional reductions in intake do not result in substantial changes the probability of adequacy. However, this does not mean there will be no significant health impacts because worsened nutrient status below the deficiency threshold can still lead to progressively more severe health consequences (Food & Nutrition Board 1998; Tanumihardjo *et al.* 2016). We have clarified this point in the Main Text (Line 209-214).

Supplementary text S3, section Dietary recall survey, first paragraph. Clarify who responded to the dietary survey for adolescents aged 11 and 12 years. If a caregiver responded, were the adolescents also present? Also, how often did the cook respond on behalf of an absent adult? This practice may allow to capture foods consumed at home during meals but is unlikely to correctly capture foods consumed as snack or away from home, so it must be shown that it was not frequent.

For adolescent girls younger than 13 years, the household cook provided responses in their presence. If the adolescent was absent, the dietary recall was not administered, thus the cook was never interviewed without the adolescent present. We have now clarified this in Supplementary text S3 (first paragraph of the *Dietary Recall* section).

Supplementary text S3, section Dietary recall survey, third paragraph. The use of number of items is described as a portion size estimation method here but not in the main text. I suggest to add this in the main text as well (lines 320-324). Also, the authors could add a sentence to clarify if the questionnaire handled leftovers. Finally, is consumption from the shared pot common in this population?

We have added a new sentence to the Methods (L380) to explain how discrete items (such as apples or packets of crisps) were quantified. Regarding leftovers, we reminded participants to only record what they consumed and not to include any leftover food when they demonstrated their portion size – we have now added a sentence to the Methods (L377) and Supplementary text S3 to clarify this. Regarding the question about consumption from a shared pot, most food is cooked for a group of people so people within a household eat food that is prepared in the same cooking vessel. However, due to cultural taboos associated with food hygiene, nobody eats from the cooking pot itself, and food is very rarely eaten off the same plate. We have added a sentence to Supplementary text S3 to make this point.

Supplementary table S1. Am I correct that the contribution of all insects to each micronutrient intake would be the sum of all insect-specific contributions? If yes, would be useful to present this sum in table S1 to figure the total contribution of pollinators to each micronutrient intake? Also, I suggest using "intake" instead of "supply". Finally, I am not sure I fully agree with the interpretation on what these numbers mean (last sentence of figure caption). To me, it would be more appropriate to conclude from the calculation that 5.12% of folate intake originated from pollination of various crops by A. cerana.

This is a good suggestion – we have now added an additional row to the table (now Table S3) which shows the total contribution of all insects to the intake of each key nutrient.

We have also changed the wording of the figure legend to 'intake' rather than 'supply' and revised the interpretation of the numbers to reflect your suggestion. This sentence now reads: "*For example, the PC score of Apis cerana for folate implies that 5.12% of folate intake is attributable to Apis cerana via its pollination (and resulting yield enhancement) of the crops that provide people's folate in this region.*"

Other specific comments:

Abstract, line 42. Which approach to which ecosystem services? I struggle to understand the sentence.

We have reworded this final sentence to explain what we mean by 'this approach'. It now reads: "Applied more widely, this approach of linking biodiversity to human health and livelihoods could reveal sustainable new pathways for enhancing the lives of billions of smallholders worldwide."

Lines 90-93: may not be needed, sounds like a repeat of lines 77-83.

Good point, we have removed a large section of this final sentence which overlapped with the sentence in the previous paragraph. It now reads:
"Our findings provide a crucial test case, offering an approach and insights relevant for improving food security and livelihoods on a global scale."

line 155: I suggest replacing “nutrition” with “diets”. I am not sure that I agree with the use of the word “impact”.

**Following your suggestions, we have changed this subheading to the following:
“*Effect of pollinator decline on diets and livelihoods*”**

Line 197: which health outcomes? Micronutrient adequacy should not be considered a health outcome.

Thank you, we have changed this sentence and no longer use the word health outcomes in this context.

Lines 203-204: which specific interventions are the authors referring to?

This section of the text has been completely removed and we now incorporate a new piece of analysis (suggested by Reviewer 1) which investigates the structural characteristics of the plant-pollinator network that supports human diets.

Line 302 : clarify list of eligible households was “obtained by census”

We’ve changed the wording (L349) to say: *“Based on a full census of each village, we identified…”*

Line 305: not sure the word “representative” is appropriate here. I suggest replacing with “diverse”.

Good point, change made.

Line 468: income that would be lost… but the absence of pollinators was not observed. Would it rather be the proportion of income attributable to pollination/pollinators in the observed scenario? As for my other suggestions below, I would not assume about too much about what would happen with change (assumptions seem very strong), but I find it interesting to present the proportion of what is observed which is attributable to pollinators.

In line with the reviewer’s suggestion, we have moderated our wording throughout the MS and now place greater emphasis on quantifying the observed contribution of pollinators to current diets and incomes. Instead of framing our scenarios as precise forecasts, we instead present them as boundary estimates that illustrate what could happen under different trajectories of pollinator decline if no mitigating interventions are in place. While these scenarios inevitably involve assumptions, they are not improbable in the context of Jumla (as we explain in detail above). Importantly, by making these assumptions explicit, the simulations highlight where interventions could reduce risks, for example, by strengthening market access to improve the availability of micronutrient-rich foods, supporting the cultivation of alternative crops, or providing economic safety nets for farmers.

Line 520: Genuine question: how does Vip depends on actual presence and concentration of the wild plant on the plot?

Good question – The Vip metric (visitation frequency of insect i to plant p) does not explicitly control for plant density; it simply reflects how frequently a given pollinator taxon was observed visiting a particular plant species. Plant abundance therefore influences Vip indirectly – plants which occurred in higher densities in our field sites tended to attract more visits, but this was not always the case. For example, some highly abundant species received very few visits and scored low on Vip, whereas some less common species (particularly certain shrubs) received disproportionally high visitation. Our aim was to quantify each plant species' overall contribution to supporting pollinators through nectar and pollen, and abundance is one important dimension of that contribution. For this reason, we did not scale visitation rates by plant density, as doing so would down-weight abundant plants and obscure their role in sustaining pollinator communities.

Supplementary text S3, section Selection of villages. Clarify in the first sentence that study villages were purposively selected. On 5th line, the word "representative" is confusing in a paragraph on selection. To avoid confusion with representativeness from random selection, I suggest to replace with a synonym such as "typical" or "similar to".

Thanks for the suggestions – we have made these recommended changes.

Table S5. Details on Z-score and anthropometric indicators calculation are not explained anywhere. They could be added (with relevant references) to supplementary text S5.

We have added a paragraph to the end of Supplementary text S5 to explain how these calculations were made and specify the references they were based on.

References cited in response to reviewers:

FAO/WHO (2004). *Vitamin and mineral requirements in human nutrition*. World Health Organization & Food and Agriculture Organization of the United Nations, Rome, Italy.

Food & Nutrition Board (1998). *Dietary reference intakes for thiamin, riboflavin, niacin, vitamin B6, folate, vitamin B12, pantothenic acid, biotin, and choline*. National Academies Press, Washington DC.

Garibaldi, L.A., Carvalheiro, L.G., Vaissiere, B.E., Gemmill-Herren, B., Hipolito, J., Freitas, B.M. *et al.* (2016). Mutually beneficial pollinator diversity and crop yield outcomes in small and large farms. *Science*, 351, 388-391.

Kortsch, S., Timberlake, T.P., Cirtwill, A.R., Sapkota, S., Rokoya, M., Devkota, K. *et al.* (2024). Decline in Honeybees and Its Consequences for Beekeepers and Crop Pollination in Western Nepal. *Insects*, 15, 281.

Markelova, H., Meinzen-Dick, R., Hellin, J. & Dohrn, S. (2009). Collective action for smallholder market access. *Food Policy*, 34, 1-7.

Nyambose, J., Tucker, K.L. & Koski, K.G. (2002). High intra/interindividual variance ratios for energy and nutrient intakes of pregnant women in rural Malawi show that many days are required to estimate usual intake. *The Journal of nutrition*, 132, 1313-1318.

Partap, U., Partap, T., Sharma, H.K., Phartiyal, P., Marma, A., Tamang, N.B. *et al.* (2012). *Value of insect pollinators to Himalayan agricultural economies*. (ed. ICIMOD). ICIMOD Kathmandu, Nepal.

Rasul, G., Hussain, A., Mahapatra, B. & Dangol, N. (2018). Food and nutrition security in the Hindu Kush Himalayan region. *Journal of the Science of Food and Agriculture*, 98, 429-438.

Smith, M.R., Mueller, N.D., Springmann, M., Sulser, T.B., Garibaldi, L.A., Gerber, J. *et al.* (2022). Pollinator Deficits, Food Consumption, and Consequences for Human Health: A Modeling Study. *Environ Health Perspect*, 130, 127003.

Tanumihardjo, S.A., Russell, R.M., Stephensen, C.B., Gannon, B.M., Craft, N.E., Haskell, M.J. *et al.* (2016). Biomarkers of Nutrition for Development (BOND)—vitamin A review. *The Journal of nutrition*, 146, 1816S-1848S.

Turo, K.J., Reilly, J.R., Fijen, T.P.M., Magrach, A. & Winfree, R. (2024). Insufficient pollinator visitation often limits yield in crop systems worldwide. *Nature Ecology & Evolution*.

U.S. Department of Agriculture, A.R.S. (2007). *USDA Table of Nutrient Retention Factors, Release 6*. U.S. Department of Agriculture, Beltsville, MD.

<u>Response to reviewer comments (Manuscript: 2025-03-06493A)</u>

We would like to express our immense gratitude to the editor and three reviewers for once again providing such helpful and positive comments on our manuscript. In response to these comments, we have implemented several further refinements that we believe strengthen the clarity and focus of the study.

Most importantly, we have made the following changes:

<u>1) Refinement of network analysis (suggested by Reviewer 2):</u> To make the new network analysis less exploratory, we now use a reduced set of species-level network metrics, chosen because they represent distinct ecological mechanisms through which pollinators could theoretically influence crop pollination and nutritional outcomes. We also incorporate pollinator abundance into the models, following the reviewer's suggestion.

<u>2) Stronger focus on locally-grown crops (suggested by Reviewer 3):</u> We have further clarified and refined our pollinator decline modelling framework, and now explicitly state that pollinator-driven yield changes are applied only to <u>locally grown</u> crops, as this makes minimal assumptions about what happens to imported crops. We show through a sensitivity analysis that this assumption has minimal influence on the results.

Regarding the title, we agree with your suggestion that "How" does not add much to the message and have adopted your suggestion of: '*Pollinators support the health and wealth of vulnerable communities*'.

All requested editorial changes have been made, though we retain the figures in-text and the combined Supplementary Information file to aid interpretation in any further reviews. The revised manuscript is submitted with tracked changes and as a clean version, as recommended. We have also responded to each of the reviewers' comment in bold below (line numbers refer to the clean version of the main text).

We thank you again for your thoughtful guidance throughout the revision process.

Referee #1 (Remarks to the Author):

I had the pleasure to read and comment on the previous submission of this manuscript. After carefully reading and considering all changes made to the revised manuscript I'd like to commend the authors to this greatly improved manuscript of an already impressive piece of work. The detail and implementations of the revisions are exemplary, further substantially strengthening the overall fundamental and applied usefulness of the study. With specific reference to my previous comments I would like to thank the authors for carrying out the network analysis, which revealed the importance of certain keystone pollinators for maintaining/increasing human nutrition in the study region. Identifying their positions in the network provides evidence for link between network structure and function, which is clearly highlighted in the manuscript. I'd like to thank the authors for make this valuable contribution to the field, which puts a spotlight on the importance of pollinators for human health.

We thank the reviewer for their positive comments and are pleased that the revised manuscript appears strengthened. We are grateful for the reviewer's initial suggestion to incorporate the network analysis, which added an important new dimension to the study. After implementing this analysis, Reviewer 2 provided constructive guidance on refining its focus and grounding the species-level metrics in clear ecological hypotheses. In response, we revised the analysis to take a more targeted approach, reducing the original set of 20 network indices to a smaller number of more meaningful species-level metrics. Reviewer 2 also recommended accounting for pollinator abundance, which can strongly influence many network metrics, so we incorporated this into our modelling framework. These refinements preserved the core intention of the analysis recommended by Reviewer 1, though they do alter the overall result and interpretation of the analysis. We believe this change was important, however, to improve the clarity and robustness of the study.

Referee #2 (Remarks to the Author):

I want to thank the authors for the detailed revision. All my comments are addressed appropriately, and I think this is a strong paper showing pollinators contribution to people. That said, in response to another reviewer, some extra analysis were added to the ms, and I have some extra questions about those.

Sorry to add noise by thinking differently that other reviewer, but I am skeptical that network structure can be easily linked to total nutrient intake. For example, there are no good hypothesis (in text, or that I can came with) relating specific plant-pollinator network metrics to total nutrient intake. In fact, as expected, the analysis shows no specific associations. I am ok leaving this exploratory analysis in text if the authors think is useful, but some details are needed.

We thank the reviewer for providing this useful feedback on our new network analysis and encouraging us to strengthen the theoretical and analytical basis for it.

For the village-level network analysis, we acknowledge that we did not have a strong theoretical basis for expecting specific network metrics to predict village-level nutrient intake, and the limited variation amongst villages made this part of the analysis inconclusive and potentially distracting. In line with the reviewer's suggestion, we have therefore removed this part of the network analysis (i.e. the village network-level analysis) and focused instead on the species-level network analysis which we agree is more interesting and informative.

Following the Reviewer's suggestion, we have refined the species-level analysis to make the underlying theory much more explicit and moved from a broad exploratory approach to a more focused set of network metrics that relate to distinct ecological mechanisms. We have also taken pollinator abundance into account, as suggested (see below for more detailed responses).

line 654: This is the network composed of the crop plants and pollinators, right? Wild plants are not included here. I'd make it explicit.

We now explicitly state that the network used for our species-level analysis is the crop–pollinator network only, and that wild plants are not included when computing the species-level network metrics (L226 and L724).

line 657: Make explicit which indexes are used. All this indexes can be calculated in many different ways (with different interpretations!). Most importantly, I could not find these network level results either in tables or figures in the supplementary.

As noted above, we have now removed the network-level analysis (and its associated indices) because it was exploratory and did not add anything important to the paper. For the species-level analysis, we now clearly specify the exact indices used, why they were chosen, and how they were interpreted (further details below).

I am more positive on the use of species level metrics, and relating network roles with its contribution to nutrient intake. I think here there are plausible hypothesis to test. However, here the analysis is done are again in a very exploratory way to my taste, and with few hypothesis on why some indexes can explain or not the outcomes.

We appreciate the reviewer's positive assessment of the species-level analysis, though we acknowledge that the original version was overly exploratory. In response, we have revised this analysis to make it more targeted and hypothesis-driven. All of these new approaches are clearly outlined in the revised Main Text (L225-239) and Methods (L714-740) and

Rather than testing the original set of 20 indices, we now focus on three species-level network metrics, each calculated from the crop–pollinator visitation network and selected *a priori* following the rationale of Coux *et al.* (2016) because they represent distinct ecological mechanisms through which pollinators could theoretically influence crop pollination and nutritional outcomes. These are: (1) *degree*, capturing the breadth of crop species visited; (2) *specialisation* (d'), reflecting the selectivity of partner use relative to availability; and (3) *pollinator species strength*, quantifying the extent to which crops depend on that particular pollinator. Together, these indices capture a pollinator's breadth of interaction partners, its relative preferences among them, and its functional importance within the crop community, and they correlate strongly with many other commonly used species-level descriptors (Coux *et al.* 2016).

We now also explicitly examine correlations among metrics and with abundance (Fig. S15), acknowledging that certain generalism-related metrics will be correlated with pollinator abundance. To avoid multicollinearity, we fit separate linear models for each metric, always including abundance as a covariate. This approach allows us to test whether each species-level metric provides additional explanatory power beyond abundance (results presented in Table S8).

321: Here I wonder if degree, nestedness rank, and weighted closeness are correlated? If so, it would be good to state this. And more importantly, I wonder if they are correlated with pollinator abundance. If so, it might be an easier measure to take than network indices, and more informative.

The reviewer is correct to point out that many species-level indices can be correlated with one another and with abundance. And indeed that abundance is a much simpler measure to record, and therefore potentially more useful. In the revised analysis, we therefore restricted the metrics to a smaller set, that represent distinct ecological mechanisms and are consequently expected to be less strongly correlated. Following the reviewer's advice, we now provide a correlation matrix (Supplementary Information Fig. S15) showing the relationships among all selected metrics and abundance. As the Reviewer correctly anticipated, some metrics (particularly degree) are closely correlated with abundance, and we now explain in the Main Text (L229) that these effects cannot be fully separated in this system.

662: By removing one species at a time, the effect of abundance might be much more important than the effect of their interaction patterns (which are likely also influenced by abundance). Acknowledging this will be important and maybe would be good to account for abundance, specially if its correlated with both, network indices and nutrient uptake.

We agree with the reviewer that abundance is likely to play a dominant role in determining nutritional impacts and that interaction patterns can themselves be shaped by abundance. Their intuition that abundance can overshadow finer-grained interaction patterns was exactly right, and it prompted an important revision to our analysis. In the updated models, abundance is now included as a predictor in all species-level analyses, and each network metric is assessed only for its additional explanatory power beyond abundance. The new correlation matrix (Supplementary Information Fig. S15) confirms the reviewer's expectation that certain metrics - particularly degree - covary strongly with abundance. We now clearly state in the Main Text (L228) and demonstrate in a new supporting figure (Fig. S14) that abundance explains most of the variation in nutritional importance, with network roles providing no additional explanatory power once abundance is accounted for.

668: Univariate analysis of 20 variables seems a bit of a fishing expedition to me. It's hard to change this now, as knowing the results, the choice of a narrower set of indices will be biased. In any case, I'd suggest showing the correlation among those indices, as many will reflect similar situations (i.e. be highly correlated). One can argue that the same species that have a high degree (and probably abundance) are also central and contribute to Nestedness, so you can not disentangle the effects. This is ok, but should be explicitly stated (if this is the case as I suspect).

We acknowledge that the original univariate analysis of 20 indices was too exploratory. Following the rationale of Coux *et al.* (2016), the revised analysis uses a reduced set of three mechanistically distinct metrics (thereby avoiding bias), and we now present the correlation structure among them and abundance (Fig. S15), as requested. We state clearly in the revised text that some effects cannot be disentangled (L229), and we focus our interpretation on whether any metric improves prediction beyond abundance, rather than on individual univariate associations.

671: Maybe I am confused here. Did you perform 20 models (one per variable) using all species across all villages? If so, the village should be in the random structure, right?

For the species-level analysis, we calculate nutritional importance and network metrics using the full crop–pollinator metaweb (all villages combined), because in many villages the sample size for individual pollinator species was too low to allow reliable estimation of species-level network metrics. As nutritional importance is therefore defined once per species across the combined network, species-level models do not require a village random effect. This model structure is now described more clearly in the Methods (L733-740).

Table S8 clarifies that you used a single predictor per model… in this case, R2 and AIC tell you the same information, and I would use only R2 for clarity. I still think many indices will be correlated and a clearer picture can be obtained to answer this question by showing a more in depth analysis.

Following the reviewer's advice, we have now conducted a more in-depth analysis where we include abundance in all models. In short, each model compares abundance-only to abundance + one network metric, or abundance + all 3 metrics together. In this new analysis where we compare models with different numbers of parameters, we believe R^2 and AIC will tell us slightly different things, so have retained both measures of model fit (Table S8). As the reviewer recommended, we now provide a comprehensive correlation matrix (Fig. S15), which clarifies the degree of overlap among predictors and supports their interpretation that abundance captures most of the relevant variation.

Finally, two small cosmetic edits:
Line 204 and 601: I would use instead of "a worst-case outcome" something more neutral such as "an extreme scenario"

We have changed "worst-case" to "extreme" throughout the manuscript, when referring to this scenario.

Line 264: Giving the lack of strong associations I would change "but also reveals" to "but also potentially reveals".

Change made

I re-state that the rest of the paper has gained in quality and should be published, once this new analysis is clarified.
Many thanks for these additional comments which have further improved the quality and theoretical rigour of our manuscript.

Referee #3 (Remarks to the Author):

I have reviewed the authors' responses to my comments and questions, as well as the related changes in the manuscript. I would like to commend the authors for their revisions which make the manuscript stronger. Overall, the manuscript is clear, well written, and provides evidence for a clear mechanistic link between pollinators and people's micronutrient adequacy and wealth, through pollinator-dependent crops yield and intake.
The re-framing of the interpretation toward the contribution of pollination to diet and wealth,

with the various scenarios presented as “boundaries”, addressed well my concern while supporting as strongly as previously the same take-away findings (if not more strongly). I like the new title and I am fine with keeping the word “health”, now associated to “support” in this new title. Indeed, I agree that “supporting health” is an appropriate attribute for micronutrient adequacy. I also appreciated the additional text (S1) and presentation of additional data to support the assumptions (e.g. figure S7, table S10). The authors also added the appropriate level of detail on diet data management and analysis in the main manuscript and supplementary file and provided references where relevant.

We are pleased to hear that our revisions have addressed most of the questions and concerns that were previously raised and we emphasise our gratitude for the original suggestions which greatly strengthened the manuscript.

Nevertheless, the revisions brought two additional comments for the reviewers.

First, the revisions brought one question related to the first simulation (scenario 1). The authors state on line 185, 204, 538 and 601 that they model complete loss of *local* pollinators. However, most descriptions in methods and results do not mention “local” and refer to “complete pollinator loss” or “pollinator-dependent share of each crop’s yield”. Could the author clarify if the relative proportional decline in yields was assumed only for local pollinator-dependent crops or if this was also applied to the imported pollinator-dependent crops? I am asking because the effect on PA of vitamin E presented on Figure 5 appears rather large, in spite of a small proportion of vitamin E intake provided by local pollinator-dependent crops as shown in new Fig 3. On line 201, the reported decline for vitamin E intake is 18%, which is consistent with Figure 4 (which shows impacts of pollinator loss), but inconsistent with table S7 also referred to (which shows a 3.9% decline associated with complete *local* pollinator loss). In table S8 of the previous version of the manuscript, it was clear that considering replacement of local crops only (i.e. due to complete “local” pollinators loss) resulted in very different results for vitamin E compared to replacement of local & imported crops (i.e. due to complete pollinators loss both locally and for imported foods). This was less true for other micronutrients. The current version of the manuscript is confusing, because it has kept only one of these two options for the new analysis (resulting in Figure S11), but it is unclear which one, and pieces of both of these options seem to be reported throughout methods and results.

We are grateful to the reviewer for raising this important point. During the previous revision, we updated our model so that pollinator declines affected both local and imported pollinator-dependent foods (with the rationale that pollinator declines are also happening globally). However, after reflecting on your comment, we now believe it is more appropriate to retain our original approach and model declines only for locally produced pollinator-dependent crops. We believe that this better reflects the central objective of the study: to quantify the direct, tractable links between local pollinators, local crop production, and human nutrition – *<u>the links over which farmers actually have some agency</u>*. Moreover, local pollinator trends can be measured with some confidence, whereas the magnitude of global pollinator trends are far more uncertain and varied.

Importantly, reverting to this initial assumption has minimal effect on our findings. As before, we conducted a full sensitivity analysis in which we repeated all simulations under the alternate assumption that imported pollinator-dependent foods decline in the same way as local crops. As shown in Table S7, this produces very similar results for the intake

of all nutrients except vitamin E, which is primarily sourced from imported soybean and sunflower oil. In Figure 4, we have now explained why vitamin E intake hardly changes, despite its high pollinator importance. This emphasises the dominant role of local pollination services in shaping nutrient intake in this setting. We have now revised the manuscript throughout to clarify this modelling decision and removed any references to the interim approach.

Other specific points related to scenario 1:
on Figure 4, please clarify what indicators are plotted for micronutrients. From the text, I assume it is intake and not PA. It should also be clarified in the figure or figure caption.

Yes that is correct - we have now added 'intake' after each of the micronutrient labels on Figure 4 to clarify that it is changes in intake rather than PA that are being shown here.

Lines 233-234: Where is the "additional 18% of the population at-risk of deficiency in at least one micronutrient" shown on graph 5? It shows additional population at-risk for each micronutrient, however, it is unclear where the 18% was found there.
Line 280: same question for "9% of the population into sufficiency in at least one micronutrient".

Apologies – these references to Figure 5 were positioned in misleading places within the sentences you mention. Figure 5 simply shows the change in Probability of Adequacy (mean across the population) that could occur under our modelled scenario of pollinator decline/enhancement. The calculations of the percentage of additional people within the population who would become deficient/sufficient in at least one nutrient was done separately and is not shown in Figure 5. We have now changed the sentences where Figure 5 is referenced, to make it clear which part of the sentence refers to Figure 5, so that it does not lead people to expect this statistic to be shown in Figure 5 (L185).

My second follow-up comment is about the nutrient requirements used by the authors to assess the probabilities of adequacy, which are now clearly referenced.
FAO/WHO. Vitamin and mineral requirements in human nutrition. (World Health 896 Organization & Food and Agriculture Organization of the United Nations, 2004).
I recognize that these requirements are the latest published by FAO and WHO, and therefore may be deemed an obvious option for use in countries where there are no published vitamin and mineral requirements. One drawback of this set of requirements is that it is very old and perhaps outdated. In 2020, Allen, Carriquiry and Murphy reviewed the most recent evidence and published a proposed set of harmonized requirements for global use, picking into IOM and EFSA requirements. Some values are rather different from the FAO/WHO values. I thought I would bring this reference to the attention of the authors, who might want to consider using this more recent set of requirements based on more recent evidence.
Reference: Allen LH, Carriquiry AL, Murphy SP. Perspective: Proposed Harmonized Nutrient Reference Values for Populations. Adv Nutr. 2020 May 1;11(3):469-483. doi: 10.1093/advances/nmz096. PMID: 31701998; PMCID: PMC7231601.

We thank the reviewer for raising this point and providing this reference to what is undoubtedly a more up-to-date set of nutrient requirements. Our decision not to use the

harmonized average requirements (H-ARs) proposed by Allen et al. (2020) is primarily methodological. The probability-of-adequacy approach that we employ requires both the estimated average requirement (EAR) and its associated standard deviation (SD). While Allen et al. provide harmonized EARs, they do not publish corresponding SD values for all nutrients. Without these SDs, it is not possible to apply the full probability method in a statistically consistent way across nutrients. Until harmonized SDs become available, our view is that it is more appropriate to rely on a single source (i.e. FAO/WHO 2004) that provides both the EAR and the distributional parameters needed for adequacy estimation.

We also note that, for the nutrients included in our analysis, the differences between the FAO/WHO EARs and the proposed harmonized EARs are relatively small in practice. Therefore, using the FAO/WHO values is unlikely to materially affect the conclusions drawn from our results.

Another minor point on requirements: in supplemental text S7, under "Nutrient requirements", it would be useful to add which sources were used for vitamin A and vitamin E requirements.

Apologies for our omission of these two nutrients. Nutrient requirement values for both vitamin A and vitamin E were also derived from FAO/WHO 2004 and this has now been stated in supplemental text S7.

Besides the changes related to my comments, I reviewed the full manuscript, including the changes resulting from other reviewers' comments. I agree these changes improved the manuscript, and I do not have further comments or suggestions on these other changes.

Many thanks once again for all of your constructive and supportive comments which have greatly improved the clarity and methodological rigour of our study.

References cited in response to reviewers:

Allen LH, Carriquiry AL, Murphy SP. Perspective: Proposed Harmonized Nutrient Reference Values for Populations. *Adv Nutr*. 2020 May 1;11(3):469-483.

Coux, C., Rader, R., Bartomeus, I. & Tylianakis, J.M. (2016). Linking species functional roles to their network roles. *Ecol Lett*, 19, 762-770.

<u>Response to reviewer comments (Manuscript: 2025-03-06493B)</u>

We thank the editor and reviewers for their continued careful assessment of our manuscript and for the constructive comments provided in this round of review. In response, we have made a small number of further revisions summarised below.

The most substantial changes relate to the species-level network analysis. In response to Referee #1, we have strengthened the theoretical framing and hypotheses underpinning the retained network metrics, expanded the metric set to include two additional descriptors of species roles, and standardised all metrics using abundance-constrained null models to explicitly separate interaction structure from abundance and sampling effects. We have also clarified the calculation and of all metrics in the Methods. These refinements confirm that pollinator abundance remains the dominant predictor of nutritional importance, with network structure adding limited additional explanatory power.

In response to Referee #3, we have improved the reporting of uncertainty in dietary adequacy metrics by adding a new supplementary table which summarises means and standard deviations across all pollination scenarios and clarified box-plot elements in all relevant figure legends.

All editorial and formatting requests have been addressed, including a small change to the manuscript title to specify 'nutritional health' instead of 'health', and 'economic status' instead of 'wealth' to be more precise in our terminology. The revised manuscript is submitted with tracked changes and as a clean version, and detailed, point-by-point responses to all reviewer comments are provided below (with line numbers referring to the clean manuscript).

We thank the editor and reviewers again for their thoughtful input throughout the review process.

Referee #1 (Remarks to the Author):

I have been asked to comment specifically on the revised network analysis after receiving further comments from Reviewer 2 on this previously added set of analyses. While I generally agree with the reviewer's comments of grounding the network analysis in a hypothetical framework that clearly outlines the expected outcomes, I have doubts whether the revision satisfactorily addresses these concerns. I'll attempt to explain my rationale below:

The authors argue that to minimise the exploratory nature of their network analysis they remove all network-level analysis from the revised paper. While I sympathise with the authors' approach, I'd like to emphasise that there is nevertheless a strong basis for using network-level metrics to support a hypothesis-driven exploration of the plant-pollinator interaction data (as done in many influential networks studies to date - but see for a more critical reflection: Dormann 2023 The rise, and possible fall, of network ecology. In: Defining Agroecology. – A Festschrift for Teja Tscharntke. Eds.: Dormann, C.F., Batáry, P., Grass, I., Klein, A.-M.,Loos, J., Scherber, C., Steffan-Dewenter, I. & Wanger, T.C. Tredition, Hamburg, pages 143-159). As the network-level analysis (using the selected metrics) did not reveal any obvious patterns, however, I agree with the argument that a more extensive analytical framework would be beyond the scope of the paper and the (other) key messages conveyed therein.

The argument of using species-level metrics to refine and direct the analytical approach is understandable and transparent. What I have difficulties with, however, is the choice of metrics, the paper(s) that support the metric selection, and the analytical approach. Equally, I find the formulation of the hypotheses relatively weak and unsupported (Line 721-723), lacking detail and references. This lack of clarity translates in the choice of species-level metrics. It is well known that abundance (and to some degree species richness) of pollinators and flowers can explain a large proportion of network patterns (see Blüthgen & Staab 2024 AREES for a recent review). The study cited by the authors to support their rationale of choice of species-level metrics (Coux et al. 2016) uses normalised degree specifically to account for network size when comparing degree between networks. It's not clear whether the authors used normalised degree (or the fully quantitative measure of proportional generality) here - please clarify. Degree in itself is also a unweighted metric, which does not incorporate some of the information benefits of weighted metrics, and comes with a risk of misinterpretation as a result of its relatively weak interpretation strength (see again Blüthgen & Staab 2024 for a more detailed reflection and references on weighted vs unweighted metrics). I can see the authors' attempt to separate unweighted (degree) from weighted (interaction strength) metrics (see also Dormann 2020 bipartite manual), but as shown in Fig. S15, they are both highly correlated with abundance. While abundance is a critical and intrinsic ecological condition of communities, network analysis can go beyond abundance patterns and reveal functional consequences (as attempted here), e.g., whether a more generalised network contributes to a higher functional performance or community stability (the authors allude to the latter but do not actually test this with their analysis).

There are, next to d', other species-level metrics that shed light on the structural roles of species, such as centrality metrics, and z-scores or null models can be used to enable comparisons across networks standardised by sampling effect or variation in abundance. Finally, beta-diversity metrics that reveal the importance of species turn-over or rewiring can provide important context on the stability of the nutritional patterns associated to plant-pollinator interactions across communities.

I recognise that these aspects were not explored in the first set of revisions, but it was my understanding that a more comprehensive (across network-level and species-level) exploratory analysis was an appropriate step towards showing the potential of the data for further in-depth network analysis by future studies. After stripping down the analysis to three species-level metrics, the message has changed somewhat, and the shortcomings listed above have become more obvious. To present the benefits of a hypothesis-driven network analysis I'd suggest that the authors keep the focus on species-level metrics but strengthen the justification and hypotheses for each individual metric used, and support the rationale of their arguments (e.g., role of individual species, stability of patterns and their impact on nutrition) with appropriate analysis (e.g. testing against null models or use z-scores to compare variation in abundance and species richness across networks). Which approach to select will depend on (the ecological relevance of) differences in sampling intensities across networks.

Finally, I'd like a clarify the authors' choice to log-transform d'. As theoretically d' can contain values of zero, a log+1 transformation is advisable. If the authors transform with log+1, there is very little/no differences between d' and (log+1)d'. Can the authors please provide a justification of the transformation to support the choice of analysis.

Response to Referee #1

We thank the Referee for their careful evaluation of the revised species-level network analysis, and for emphasising the need for: (a) a clearer theoretical basis and explicit hypotheses for our metric choice, and (b) an analytical framework that separates abundance and sampling effects from interaction structure.

In response, we have: (i) strengthened the hypotheses and justification for each retained metric, (ii) expanded the set of species-level metrics to include centrality and 'crop focus' (explained below), (iii) explicitly standardised all metrics using abundance-constrained null models, and (iv) clarified the calculation and transformation of all metrics in the Methods.

Before detailing these revisions, we would like to reiterate the primary aim and intended audience of this particular study. We hope to convince a broad audience in the agricultural and nutritional sectors that interactions between plants and pollinators are important for human nutrition. Any exploration of the details of pollination network structure (while of considerable interest to pollination network ecologists) is secondary to this overall goal and must be framed in terms that are comprehensible to a general audience. While we agree that network-level and species-level metrics can offer useful insights into the functional roles of an ecological community, an exhaustive exploration of network structure (e.g. stability, rewiring versus turnover, or full classification of species roles) would be beyond the intended scope of this manuscript and we hope that the Referee will understand our desire to explore them fully in a subsequent network theory focused publication where they are likely to be read by a different audience. Nevertheless, given that we identify which insects contribute most strongly to nutrient provision, we see clear value in briefly examining which network properties of these insects might be associated with this contribution. And we fully agree that any network analysis we retain here should be rigorous, transparent, and supported by clear hypotheses, so we have revised both the framing and the analysis accordingly.

From an applied perspective, particularly for development or agricultural agencies seeking practical guidance, metrics that are conceptually accessible will be more relevant than those that are mathematically optimal but difficult to estimate or communicate outside specialist contexts. For this reason (and despite some shortcomings), we retain simple descriptors including unweighted degree, pollinator abundance, and an added metric we term 'crop focus' (% of total visits made to crop plants), while supplementing them with more refined metrics of interest to network ecologists (see below). Importantly, all retained metrics are now evaluated relative to abundance-constrained null expectations (see further details below), directly addressing the concern that raw network metrics may largely reflect abundance or sampling effects.

Guided by the practical recommendations in Blüthgen & Staab (2024), which you kindly pointed us to and which we found extremely helpful, we now organise our species-level predictors into a small number of complementary "dimensions" of network complexity, and restrict the analysis to metrics that are straightforward to interpret and for which we can reasonably hypothesise a link to nutritional outcomes; these are outlined below (and in the revised methods; L731-774).

<u>**Species-level metrics and plausible links to nutritional outcomes**</u>

Pollinator abundance (quantified as total visitation frequency across all plants) is treated as a baseline predictor in all models, as it represents the most direct proxy for pollination activity and a fundamental ecological driver of both function and observed network structure (Chacoff *et al.* 2018;

Blüthgen & Staab 2024). We hypothesise that more abundant pollinators will, on average, contribute more strongly to crop pollination and nutrient provision.

To capture how pollinator activity is distributed across plants, we include **(1) interaction breadth (degree)** and **(2) interaction evenness (Shannon diversity),** both calculated on the full plant–pollinator metaweb (including crops and wild plants). These metrics represent how widely and how evenly pollinators interact across plant partners (Bersier *et al.* 2002; Blüthgen *et al.* 2007) and therefore the range of crops and nutrients they could potentially support. Degree is interpreted strictly as a measure of partner range, while Shannon diversity captures how visits are distributed among partners. Because all analyses relating network roles to species properties are performed on the pooled metaweb across all villages (rather than comparing across networks of different sizes), we do not use normalised degree. We use the metaweb in order to include as much sampling effort as possible for each insect, although we do hope to explore variation between villages in a separate study.

We also quantify **(3) interaction selectivity using Blüthgen's d′**, which measures deviations between observed interaction strengths and those expected given overall plant visitation rates (Blüthgen *et al.* 2006). This metric allows us to test whether a pollinator's interaction preferences (beyond abundance and partner number alone), are associated with nutritional importance.

In response to the Referee's suggestion to consider additional structural role metrics, we now include **(4) closeness centrality** (weighted closeness), calculated on the full network. Closeness centrality reflects a species' average distance to all others and is commonly interpreted as a proxy for its potential to influence network-wide dynamics (Delmas *et al.* 2019). In this context, we hypothesise that centrally positioned pollinators may affect pollination across multiple crops and nutrients. We selected closeness centrality over other centrality measures (e.g. PageRank or eigenvector centrality) because of its relatively intuitive interpretation, which we consider important for maintaining relevance to readers from agricultural and nutritional backgrounds.

Finally, to explicitly capture how pollinator activity is allocated between crop and wild plants, we introduce a metric we term **(5) "crop focus",** defined as the proportion of a pollinator's total visits that are directed to crop species. This metric distinguishes pollinators that concentrate their activity on crops from those that primarily visit wild plants, independent of overall activity level, and directly links interaction patterns to nutritional outcomes.

Null models / z-scores to address abundance and sampling effects

Following the Referee's suggestion, we now base our analysis on null-standardised z-scores rather than raw metric values. Specifically, we generate quantitative null networks that preserve each pollinator's total number of visits (column sums) while randomising the distribution of visits across plants (method 'c0_ind' in the commsim function in vegan). This approach holds pollinator abundance constant while randomising interaction structure, allowing us to test whether observed network properties are more extreme than expected based on abundance alone.

While abundance was fixed in the null models, z-scores for several network properties remained correlated with abundance across species. Abundant species tended to have higher closeness centrality and lower degree and interaction diversity than expected purely based on their abundances (z-scores of crop focus and d' were weakly and negatively correlated with abundance) suggesting that insects' network roles are not fully determined by their abundance, although the network measures we considered do not add much to our understanding of crop pollination services. All statistical models

include abundance as a baseline predictor, and each z-standardised network metric is evaluated only for its additional explanatory power beyond abundance. Despite the expanded set of metrics and null-standardisation, these network descriptors explained little additional variation in nutritional importance beyond the abundance-only model, consistent with the results presented in the revised main text.

Overall, we hope that these revisions provide a clearer rationale for our selection of metrics and a more rigorous analysis for isolating structural signals from abundance/sampling effects, while avoiding becoming too heavily focused on network analysis and detracting from the main message of the manuscript.

Referee #2 (Remarks to the Author):

Thanks for revisiting the network analysis. I am very happy with the current implementation, and I think that despite the less shiny results regarding network importance, the result showing abundance is driving most of the responses is very relevant.

I have nothing else to add. Congratulations for this nice piece of work.

We thank the referee for their positive evaluation of the revised network analysis and for their original encouragement to strengthen its theoretical grounding and clarity. Following additional comments from Referee #1, we have made further refinements to this section (see our responses to Referee #1 above). Importantly, the overall results and interpretation of the analysis remain unchanged, and pollinator abundance is still the strongest predictor of nutritional importance. The additional revisions do not introduce new analytical directions; rather they reinforce the theoretical justification and methodological rigour of our existing analysis - most notably through the use of null models to explicitly account for abundance and sampling effects. We hope these clarifications strengthen the manuscript while remaining fully consistent with the revised framework that the Referee initially suggested.

Referee #3 (Remarks to the Author):

I reviewed the third version of the manuscript “Pollinators support the health and wealth of vulnerable communities”. I confirm that my concerns were adequately addressed.
I appreciate the clarified focus on locally produced pollinator-dependent crops, with a sensitivity analysis showing the effect of including non-local crops into the scenarios.
I understand that the authors preferred not to switch vitamin and minerals requirements. Indeed, calculating probabilities of adequacy with the Allen and al. harmonized requirements would imply going back to the detailed documentation of each of the EFSA and IOM selected requirements and extract the coefficients of variation. As the methods are clear on the use of the WHO requirements, I am fine with the author’s decision to not switch requirements.
Overall, the paper makes an important contribution to the field, showing the magnitude of the contribution of pollination and specific pollinators to dietary adequacy and income in an exemplary context, and supports a call to facilitate management of local pollination services. I learned a lot from reviewing this manuscript.

In addition, as requested, I paid particular attention to the statistics and error bars. The variability of PAs is not presented on Figure 5, although adding it would make the figure very difficult to read. One option (to be confirmed if acceptable by the editorial team) might be to clarify in the figure legend that SDs for the current pollination scenario are presented in Table S6. I don't seem to see anywhere indications on the distribution of PAs estimates with the "no pollination" and "optimum pollination" scenarios, does it need to be added? Where relevant, error bars are appropriately used and what they represent is defined in the corresponding figure legends. It seems to me that the Journal is expecting a full definition of all box-plot elements ; this would not be currently provided in Figure S17. One last point: There seems to be a small mismatch between Figure 3 and Table S6 in the mean for adult women's PA of vitamin A and calcium (current scenario).

We thank the reviewer for their re-evaluation of the manuscript and for their positive assessment of the revised work. We are pleased that the previous concerns have been adequately addressed and appreciate the reviewer's constructive feedback.

In response to the remaining points, we have added a new supplementary table (Table S8) summarising the mean and standard deviation of probabilities of adequacy (PA), including mean probability of adequacy (MPA), for all population subgroups across the current, no-pollination, and optimum pollination scenarios. This provides a clear summary of the distribution of PA estimates across all scenarios.

We have also clarified the definition of all box-plot elements in the relevant figure legends, including Figure S17, in line with the journal's requirements. All figures and tables have been regenerated using the final version of the analysis to ensure full consistency across the manuscript, resolving the previously noted minor mismatch between Figure 3 and Table S6 for adult women's vitamin A and calcium PA under the current pollination scenario.

References cited:

1.
Bersier, L.-F., Banašek-Richter, C. & Cattin, M.-F. (2002). Quantitative descriptors of food-web matrices. *Ecology*, 83, 2394-2407.

2.
Blüthgen, N., Menzel, F. & Blüthgen, N. (2006). Measuring specialization in species interaction networks. *BMC Ecology*, 6, 9.

3.
Blüthgen, N., Menzel, F., Hovestadt, T., Fiala, B. & Blüthgen, N. (2007). Specialization, Constraints, and Conflicting Interests in Mutualistic Networks. *Curr Biol*, 17, 341-346.

4.
Blüthgen, N. & Staab, M. (2024). A critical evaluation of network approaches for studying species interactions. *Annual Review of Ecology, Evolution, and Systematics*, 55.

5.
Chacoff, N.P., Resasco, J. & Vázquez, D.P. (2018). Interaction frequency, network position, and the temporal persistence of interactions in a plant–pollinator network. *Ecology*, 99, 21-28.

6.
Delmas, E., Besson, M., Brice, M.H., Burkle, L.A., Dalla Riva, G.V., Fortin, M.J. *et al.* (2019). Analysing ecological networks of species interactions. *Biological Reviews*, 94, 16-36.
